# Size increase without genetic divergence in the Eurasian water shrew *Neomys fodiens*

**DOI:** 10.1038/s41598-019-53891-y

**Published:** 2019-11-22

**Authors:** Alfonso Balmori-de la Puente, Carlos Nores, Jacinto Román, Angel Fernández-González, Pere Aymerich, Joaquim Gosálbez, Lídia Escoda, Jose Castresana

**Affiliations:** 1 Institute of Evolutionary Biology (CSIC-Universitat Pompeu Fabra), Passeig Marítim de la Barceloneta 37, 08003 Barcelona, Spain; 2 Indurot, Universidad de Oviedo, Campus de Mieres, 33600 Mieres, Asturias Spain; 3 Department of Conservation Biology, Doñana Biological Station, CSIC, Calle Americo Vespucio 26, 41092 Sevilla, Spain; 4 Biosfera Consultoría Medioambiental S.L., Calle Candamo 5, 33012 Oviedo, Spain; 5 Calle Barcelona 29, 08600 Berga, Barcelona Spain; 6 Department of Evolutionary Biology, Ecology and Environmental Sciences, University of Barcelona, Avinguda Diagonal 645, 08028 Barcelona, Spain

**Keywords:** Evolutionary ecology, Evolutionary genetics, Phylogenetics, Population genetics

## Abstract

When a population shows a marked morphological change, it is important to know whether that population is genetically distinct; if it is not, the novel trait could correspond to an adaptation that might be of great ecological interest. Here, we studied a subspecies of water shrew, *Neomys fodiens niethammeri*, which is found in a narrow strip of the northern Iberian Peninsula. This subspecies presents an abrupt increase in skull size when compared to the rest of the Eurasian population, which has led to the suggestion that it is actually a different species. Skulls obtained from owl pellets collected over the last 50 years allowed us to perform a morphometric analysis in addition to an extensive multilocus analysis based on short intron fragments successfully amplified from these degraded samples. Interestingly, no genetic divergence was detected using either mitochondrial or nuclear data. Additionally, an allele frequency analysis revealed no significant genetic differentiation. The absence of genetic divergence and differentiation revealed here indicate that the large form of *N. fodiens* does not correspond to a different species and instead represents an extreme case of size increase, of possible adaptive value, which deserves further investigation.

## Introduction

Phylogeographic studies allow researchers to understand not only the geographical distribution of genetic diversity, but also the evolution of phenotypic differences that might have adaptive potential^1,2^. Changes in body size are among the most important sources of phenotypic variation in endothermic vertebrates^3,4^. They can occur across a clinal variation as a consequence of the changing environment^5^, in islands due to ecological release^6^, or be restricted to a limited region within a species’ range due to local adaptation^7^. In some of these cases, populations with different body sizes are recognized as subspecies. Other instances of body-size modification in specific areas do not result in taxonomic changes as they are likely to correspond to ecotypes that can exploit new resources, more accessible to individuals with either smaller or larger body sizes^8–10^. Understanding the origin and evolution of such body size changes and whether these arose through a process of isolation or a rapid ecological adaptation requires a thorough phylogeographic and population genetics analysis, something that many studies on this topic lack.

The Eurasian water shrew (*Neomys fodiens*) is a good example of where the relationship between body size variation and phylogeography can be studied. This species has a Eurasian distribution, ranging from the northern Iberian Peninsula to eastern Asia^11^. It is a semi-aquatic species that inhabits water courses and other wetland habitats with abundant invertebrate prey^11,12^. Among the four species described in the genus^13^, *N. fodiens* is considered the most aquatic due to its excellent diving ability, although it sometimes demonstrates terrestrial behavior^12,14^.

Several subspecies have been proposed for *N. fodiens*, but none has been adequately assessed, with the exception of *N. f. niethammeri*^15^. This taxon is present in the north-central Iberian Peninsula, from the Cantabrian Range to the western end of the Pyrenees (Supplementary Fig. S1)^16–19^, and is characterized by the largest skull size observed throughout the range of *N. fodiens*^19^. This size increase in a specific population is striking, as the morphology of this species is fairly stable across the rest of its European range^20^. *N. f. niethammeri* lives sympatrically with *N. anomalus*^21^, which was recently separated from the European species *N. milleri*^13,22^. A population of the nominal subspecies *N. f. fodiens* is found beyond the range of *N. f. niethammeri*^12^, making that the population of *N. f. niethammeri* is flanked by populations of *N. f. fodiens* (Supplementary Fig. S1). Coronoid height has been widely used to differentiate *Neomys* species in paleontological and neontological studies^17,23–25^, and is also the main character to distinguish *N. f. niethammeri*^16–19^. Due to the much larger size of the latter, it has been suggested that it is a different species in numerous studies, including the most important reference works on mammals^11,15,18,19^.

Improved laboratory techniques, many adopted from ancient DNA studies that target low quality and degraded DNA, have enabled the use of non-invasive and minimally invasive samples as well as different types of post mortem remains in phylogeographic studies^26,27^. This has made it possible to genetically analyze elusive and threatened species, as larger sample sets that do not require the manipulation or capture of specimens become available for study^22,28,29^. For example, skull bones obtained from owl pellets containing undigested material from small mammal prey have been used in various phylogeographic studies^30–32^. This material is very interesting as it can be used for both morphological and genetic work. However, despite this potential, few studies have taken advantage of skull bones from owl pellets for simultaneous genetic and morphometric analyses and, in particular, to recover nuclear data.

Mitochondrial DNA is more abundant than nuclear DNA in animal cells and can be more easily amplified from degraded samples. Nevertheless, mitochondrial introgression, which is a common phenomenon in mammals, may lead to erroneous conclusions if only mitochondrial genes are considered^33,34^. The development of highly variable nuclear markers avoids the problems of using solely mitochondrial sequences and allows the use of coalescent-based methods^35,36^, thus leading to robust conclusions on the origin and evolution of biodiversity^13,33,37,38^. It is therefore important to develop nuclear markers that can be amplified from degraded samples, in which the DNA fragment size is shorter. In order to amplify these short fragments, primers need to be designed to reduce the size of the PCR product so that more DNA becomes available for amplification, thus exploiting the full potential of multilocus information.

The main objective of this work was to understand whether the size increase observed in *N. f. niethammeri* is linked to genetic distinctiveness. To discern this, we first designed novel nuclear introns that could be amplified using skull bones extracted from barn owl pellets collected over recent decades. From this material we then amplified mitochondrial and nuclear markers, and performed a morphological analysis as well as a multilocus study of genetic divergence, differentiation, and diversity. The results showed that the relationship between phenotypic novelty and phylogeography is not always easily predictable.

## Results

### Morphometric analyses of *Neomys* mandibles

**Figure 1:**
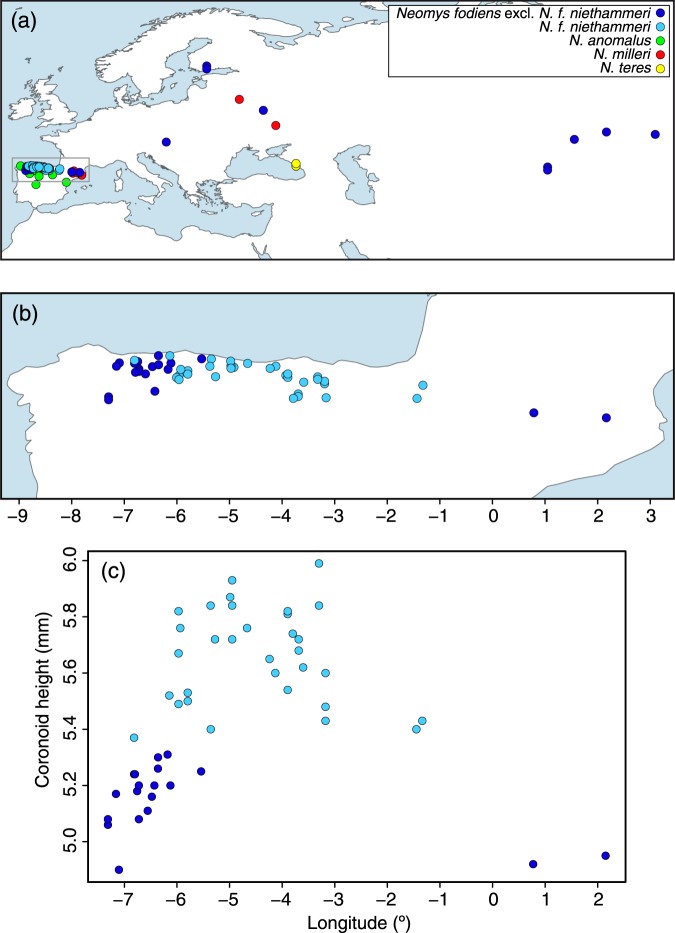
Distribution of the samples used in this study. (**a**) Map of all *Neomys* samples with the main study area highlighted, (**b**) enlargement of the northern Iberian Peninsula showing only *N. fodiens* specimens, and (**c**) plot across longitude in the Iberian Peninsula showing differences in skull size as measured using the coronoid height for *N. fodiens*. Samples from Igea *et al*.^13^ are included but sequences from databases are not. Note that the samples of *N. fodiens* not corresponding to *N. f. niethammeri* may include several subspecies: the European specimens most likely correspond to *N. f. fodiens* but those from Central Asia may belong to other subspecies whose ranges are not clearly delimited.

**Figure 2:**
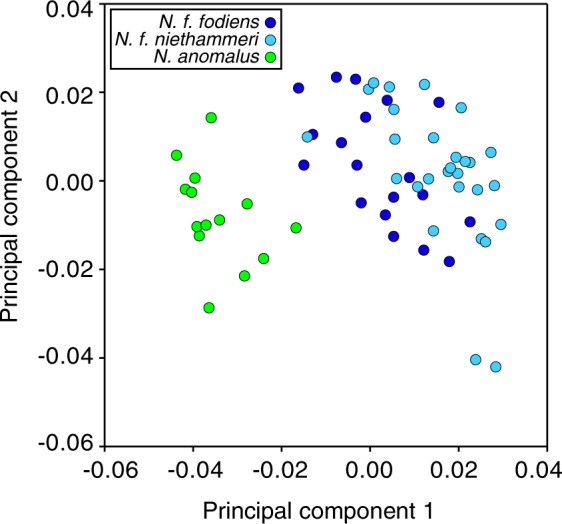
Principal components analysis of the mandible landmarks of Iberian *N. fodiens* and *N. anomalus* individuals.

Skull samples from 67 *Neomys* individuals (Supplementary Table S1) were obtained, primarily, from barn owl pellets collected over the last 50 years in the northern Iberian Peninsula. The samples came from approximately longitudes −7° to 2°, where two subspecies of *N. fodiens*, as well as *N. anomalus*, are present (Fig. 1). Landmarks were taken from each mandible (Supplementary Appendix S1) to measure the coronoid height and perform a geometric morphometric analysis. According to the coronoid height (Supplementary Fig. S2), 32 samples were classified as *N. f. niethammeri*, 19 as *N. f. fodiens*, and 16 as *N. anomalus* (Supplementary Table S2). Plotting these measurements against longitude, we confirmed an abrupt size increase in *N. f. niethammeri* in the north-central part of the Iberian Peninsula, approximately between longitudes −6.25° and −1° (Fig. 1c), corroborating previous work^17,18^. We found individuals of both sizes in the same locality, indicating a certain overlap in the distribution of the two groups. The skulls of the sympatric species *N. anomalus* were always smaller than those of *N. fodiens* (Supplementary Table S2). The centroid size of the mandibles provided similar results (Supplementary Fig. S3 and Supplementary Table S2). A principal components analysis of the landmarks allowed *N. anomalus* to be distinguished from *N. fodiens*, but not *N. f. niethammeri* from *N. f. fodiens* (Fig. 2), indicating that the two *N. fodiens* subspecies were similar in shape.

### Mitochondrial phylogeny of *Neomys*

**Figure 3:**
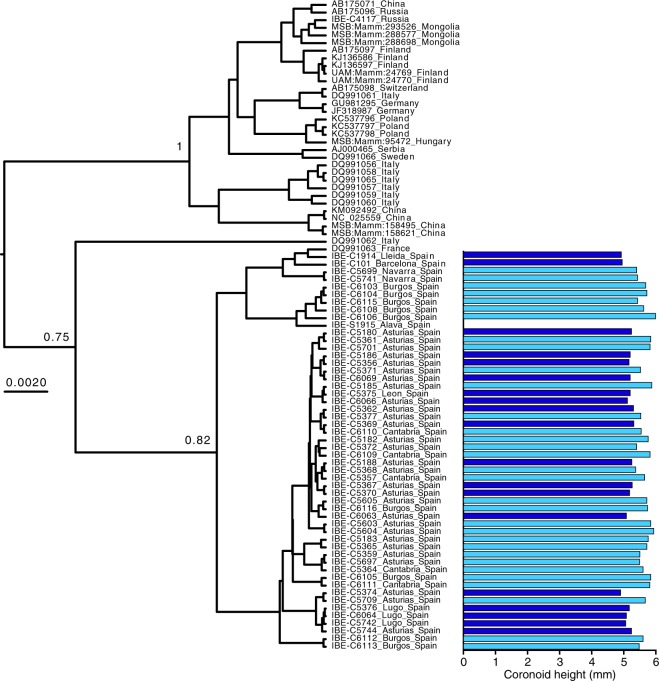
Bayesian tree of *N. fodiens* cytochrome *b* sequences. Samples with coronoid height measurement are represented with color-coded bars showing their skull size: dark blue for *N. f. fodiens* and light blue for *N. f. niethammeri*. The scale is in substitutions per position and posterior probabilities are indicated for the clades mentioned in the text.

Both complete and partial mitochondrial cytochrome *b* sequences were obtained from 85 samples of *N. fodiens*, including all the mandibles used in the morphometric analysis plus additional tissue samples from Eurasia (Fig. 1 and Supplementary Table S1). Some primers were newly designed (Supplementary Table S3) so that sequences could be obtained from the majority of samples, including the oldest (Supplementary Table S1). The Bayesian mitochondrial tree reconstructed with the *N. fodiens* mitochondrial sequences can be subdivided into three main clades separated by relatively long branches and moderate or high support (Fig. 3): one includes the Iberian samples and a sample from a nearby locality in France; the second consists of a single sample from southern Italy; and the third comprises samples from the remaining Eurasian range of the species. Coronoid height measurements mapped into this tree indicated that individuals classified as *N. f. niethammeri* appeared randomly across the Iberian clade, reflecting the fact that the two subspecies were indistinguishable at the mitochondrial level. When additional sequences from other *Neomys* species were included in the Bayesian phylogenetic analysis to configure a dataset of 136 sequences, the tree perfectly separated the four species in the genus, but *N. f. fodiens* and *N. f. niethammeri* were once again intermixed in the tree (Supplementary Fig. S4). Similar results were obtained with a maximum-likelihood method (Supplementary Fig. S5).

### Development of nuclear markers for degraded *Neomys* samples

We developed a set of six short intron markers (ASB6 intron 2, CSF2 intron 2, GDAP1 intron 1, JMJD intron 2, MYCBPAP intron 11, and TRAIP intron 8) that could be amplified using DNA extracted from the mandibles, despite the high degradation levels of some of them (Supplementary Table S4). For some introns, several rounds of primer design were performed to shorten the PCR product and allow the amplification of the most degraded samples (Supplementary Table S4). In this way, we obtained nuclear information from most of the recent samples, as well as from a good proportion of the older samples, including those collected during the 1970s (Supplementary Table S1). For some introns, allele-specific primers were designed to separate heterozygous sequences (Supplementary Table S5). A total of 58 samples with a minimum of four sequenced introns were used in further analyses, including 37 mandibles and 11 tissues, together with sequences from 10 samples taken from a previous work^13^ (Supplementary Table S1). Considering all the introns together, 172 sequences were used from *N. f. niethammeri*, 250 from the other *N. fodiens* specimens, 158 from *N. anomalus*, 48 from *N. milleri*, and 24 from *N. teres*, totaling 123,556 bp of nuclear sequence information after alignment cleaning (Supplementary Appendix S2).

### Multilocus phylogenetic analysis of *Neomys*

**Figure 4:**
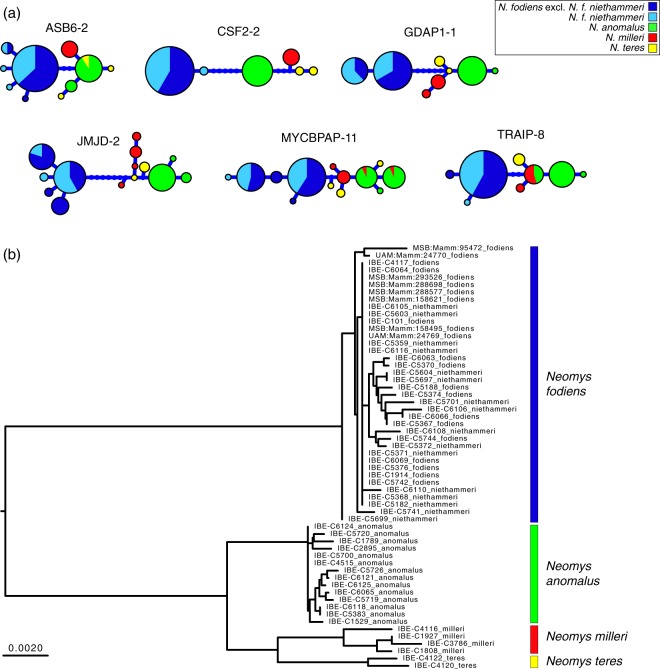
Phylogenetic information derived from the 6 introns amplified in the *Neomys* samples. (**a**) Haplotype genealogies where the size of the circles is proportional to the number of alleles detected. (**b**) Distance tree for the concatenated introns with the scale in substitutions per position.

Haplotype genealogies derived from the maximum-likelihood trees of the nuclear sequences showed a low degree of allele sharing between the four *Neomys* species (Fig. 4a). On the other hand, *N. f. niethammeri* and the rest of *N. fodiens* shared the most frequent alleles (largest circles in Fig. 4a), although most minor alleles were exclusive to one group or the other (smaller circles in Fig. 4a). Since the mutational differences between the alleles were minimal, both groups were completely intermixed in the phylogenetic tree reconstructed using the concatenated intron sequences (Fig. 4b). In fact, no clades within *N. fodiens* can be distinguished in the nuclear tree.

### Genetic differentiation

To study the possibility that differentiated groups existed within *N. fodiens*, we next converted the six intron alignments into a multi-allelic data format. There were 22 different alleles and therefore the average number of alleles per locus was 3.7. Using this information, we calculated the F_ST_ distance between *N. f. niethammeri*, *N. f. fodiens* from the northern Iberian Peninsula, and *N. fodiens* from the rest of the range. The value obtained between *N. f. niethammeri* and Iberian *N. f. fodiens* was 0.040 and it was not significant (95% confidence interval: −0.005, 0.085). F_ST_ values between these and the other population were also non-significant.

### Nuclear genetic diversity of *N. fodiens* populations

**Figure 5:**
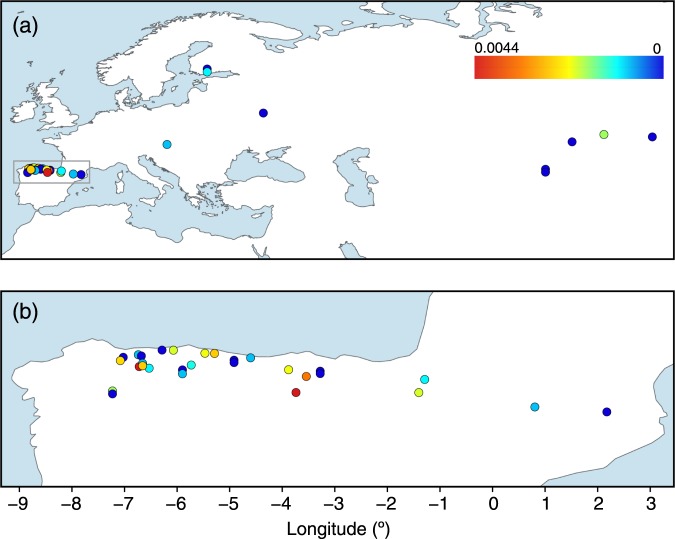
Map of color-coded individual heterozygosity rates in *N. fodiens*. (**a**) Map of all samples with the main study area highlighted, and (**b**) enlargement of the northern Iberian Peninsula. The scale is in number of heterozygous positions per base.

Individual heterozygosity estimated from the nuclear introns of *N. fodiens* specimens showed that the most heterozygous samples were from the northern Iberian Peninsula (Fig. 5). Thus, we found an average of 0.0013 heterozygous positions in the Iberian samples (including both *N. f. fodiens* and *N. f. niethammeri*) versus 0.0004 in the rest of the sampled range. When the nuclear data was analyzed at the population level, the average θ values were 0.0021 and 0.0014 for these two groups, respectively (Supplementary Table S6), showing the same trend. The same was found with the π parameter (Supplementary Table S6).

## Discussion

The multilocus dataset used in this study of the Eurasian water shrew *Neomys fodiens* was based on the mitochondrial cytochrome *b* gene and six small intron fragments. The lengths of the newly designed introns (153–229 bp) were smaller than those of previously proposed intron markers^13,37^, in order to facilitate the amplification of degraded DNA obtained from skulls from owl pellet material. Using the novel primers, we amplified between 4 and 6 introns from 37 mandible samples of *Neomys*, many of which had been collected during the 1970s (Supplementary Table S1). Despite their relatively short lengths, the intron markers were variable enough to detect mutational differences between species. They also enabled the reconstruction of a nuclear phylogenetic tree, which was compared with the mitochondrial tree and used to detect any possible occurrence of mito-nuclear discordance in *Neomys*. Finally, once these intron markers were converted to allele frequency data, and despite the small number of markers used, they allowed us to undertake a differentiation analysis of the main populations of *N. fodiens*. Therefore, thanks to the various genetic analyses performed, these novel markers were highly useful in unraveling the evolutionary history of *N. f. niethammeri* and, specifically, assessing whether the morphological differences observed (large skull size) arose through a long period of isolation and genetic differentiation. Furthermore, to our knowledge, this is one of the first studies where multilocus genetic data as well as morphometric information of skulls obtained from owl pellets has been fully exploited, showing the enormous potential of this type of non-invasive sampling for biodiversity studies.

The mitochondrial sequences of *N. f. fodiens* and *N. f. niethammeri* were intermixed in the phylogenetic tree, meaning that there was no evidence to suggest genetic divergence (Figs 3 and S4). This was a highly unexpected result for two populations with important skull size differences. Without further data, a possible explanation could be that, in fact, these two forms were more divergent at the nuclear level but, due to some recent unidirectional introgression event, *N. f. niethammeri* acquired the mitochondria of *N. f. fodiens*, a phenomenon that has been observed in many other species^33,34^. With *N. anomalus* living sympatrically with *N. fodiens* in the northern Iberian Peninsula, additional scenarios involving inter-specific introgression with this other species could not be discarded, demonstrating the need to carry out a nuclear analysis. The phylogenetic tree we obtained using nuclear data also revealed a lack of genetic divergence between the two *N. fodiens* subspecies (Fig. 4b). A first conclusion from this work is, therefore, that our mitochondrial and nuclear data are consistent in highlighting a lack of support for genetic divergence between *N. f. fodiens* and *N. f. niethammeri*.

The mitochondrial and nuclear phylogenies showed that the four *Neomys* species were reciprocally monophyletic in both trees (Figs 4b and S4). This suggests that there has been no recent mitochondrial introgression, not only between *N. fodiens* groups, but also during the evolution of the *Neomys* species. Incidentally, the topological relationships between the four *Neomys* species were not coincident in the mitochondrial and nuclear trees, since *N. milleri* and *N. anomalus* group in the former, whereas *N. milleri* and *N. teres* appear as sister taxa in the latter. However, this could be a consequence of methodological difficulties in resolving old divergences in the tree or incomplete lineage sorting^39^, and it does not affect the conclusion that there has been no recent introgression in *Neomys*. This reasonable mito-nuclear agreement enables the use of cytochrome *b* for species identification from non-invasive samples in further ecological studies of this genus^22^.

Populations that have been isolated for a short period of time may not have accumulated enough mutations to reflect phylogenetic separation, but, if the gene flow between them is low, differences in allele frequencies may appear by genetic drift^40,41^. To test the possibility that *N. fodiens* populations showed some degree of differentiation, we computed F_ST_ statistics. No significant genetic differentiation was found. Thus, the increase in skull size of *N. f. niethammeri* (Fig. 1c) corresponded to neither a difference in shape (Fig. 2) nor mitochondrial (Fig. 3) or nuclear genetic divergence (Fig. 4b), nor genetic differentiation of nuclear allele frequencies. In this respect, it is interesting to note that fossil data of both forms have been found at ~40 kyr^24,25^. This would suggest that a large amount of gene flow must have occurred between the two forms to prevent genetic differentiation during all this time, indicating the lack of reproductive barriers between them.

The populations of *N. fodiens* of the northern Iberian Peninsula are at the edge of the Palearctic range of the species (Supplementary Fig. S1), which could lead to the hypothesis that this area was recently colonized from the multiple European glacial refugia that have been described for various taxa^42,43^. We found, however, that the nuclear genetic diversity was higher in the Iberian samples than in the rest of the Palearctic samples analyzed here (Fig. 5), something that is not consistent with the recent colonization of the Iberian Peninsula. The existence in this area of fossil *N. fodiens* dated at ~40 kyr^24,25,44^ also supports the idea that the species was present in the Iberian Peninsula long before the Last Glacial Maximum and, consequently, that these populations are not the product of recent colonization. Instead, refugia in the Iberian Peninsula or nearby areas were likely to have been the source for the recolonization of at least some parts of the western Palearctic^45,46^.

With regard to the taxonomic debate surrounding *N. f. niethammeri*, the phylogenetic analyses performed here, using mitochondrial and intron sequences, indicate that *N. f. niethammeri* has not accumulated measurable genetic divergence with respect to *N. f. fodiens*. There is also no significant genetic differentiation between them, meaning that also allele frequencies are similar. Taking all this information into account, it is clear that *N. f. niethammeri* cannot be considered an independent species, contradicting previous studies where it was suggested that *N. f. niethammeri* might warrant species status^11,15,18,19^. An alternative suggestion is that *N. f. niethammeri* is an ecotype, although further work would be required to corroborate this point.

Indeed, the lack of genetic divergence and differentiation revealed here suggests that the large skull of *N. f. niethammeri* possibly arose as an adaptation to the environment. Two main hypotheses can be used to explain this. Firstly, a previous hypothesis proposed that more calcareous substrates present in the central part of the Cantabrian Range could have resulted in rivers richer in nutrients and a consequent selection of individuals with better-developed mandibles to capture larger prey^17,18^. However, *N. fodiens* lives in other areas with calcareous substrates where it displays no change in body size, so this is unlikely to have been the sole driver. Alternatively, the size increase of *N. f. niethammeri* could have resulted from ecological displacement due to competition with the Iberian endemic *N. anomalus*, similar to that observed in other mammals^47,48^. An increase in the size of *N. f. niethammeri* could have favored access to new resources, for example larger prey, thus limiting competition with *N. anomalus*. In principle, both species occupy the same aquatic habitat in the northern Iberian Peninsula and most of the range of *N. f. niethammeri* overlaps with that of *N. anomalus*^12,21^, making competition between the two species possible. The interaction of *N. fodiens* with *N. milleri* has been studied in Europe, where both species live sympatrically^49^. However, the interaction of *N. fodiens* with *N. anomalus* when they live sympatrically has never before been studied, and this may be totally different to that which occurs when it coincides with *N. milleri*, so we do not know if and how this interaction could have stimulated a size increase in *N. f. niethammeri*. We therefore suggest that future studies be directed at understanding the micro-habitat and inter-specific interactions of these *Neomys* species. We hope that a combined genetic and ecological approach will help unravel why *N. f. niethammeri* experienced an abrupt increase in skull size across a narrow strip in the Iberian Peninsula, and reveal the evolutionary advantages and possible ecological consequences of this phenotypic novelty.

## Methods

### Sample collection

A total of 79 samples from the Palearctic range of the genus *Neomys*, with special emphasis on *N. f. niethammeri* and *N. f. fodiens* from the Iberian Peninsula, were analyzed (Fig. 1 and Supplementary Table S1). These included 68 skull samples from barn owl pellets collected in the field, of which 65 could be used for genetics and morphometry, and 3 could only be used for genetic analysis. Some of these samples had been analyzed in a previous morphological study of *N. f. niethammeri*^17^. In addition, 9 tissue samples were loaned from museums and colleagues, and 2 tissue samples were taken from our own collection.

To complete the phylogenetic analysis, cytochrome *b* and nuclear sequences of 18 *Neomys* samples were taken from Igea *et al*.^13^ and 39 cytochrome *b* sequences were downloaded from GenBank^50^. Two of the *N. fodiens* skull samples available from Igea *et al*.^13^ were used for morphometry.

### Ethics statement

No live animals were collected for this study and therefore no ethics permit was necessary.

### Measurements and geometric morphometric analysis

After cleaning the skulls extracted from the owl pellets, we used only one lower mandible per skull and the rest was stored. Images of the 67 mandibles, together with a scale, were taken with a Canon 100D camera and a Canon EF-S 60 mm f/2.8 macro objective. The ImageJ 1.50i^51^ program was then utilized to take 16 landmarks from each sample (Supplementary Fig. S2 and Supplementary Table S2). Many of the mandibles were partially broken or without teeth due to the digestion process so that no landmarks were taken in teeth. Landmarks 1 and 7 were used to calculate the coronoid height (for six partial mandibles, only these two landmarks could be obtained). According to previous works, the coronoid height ranges considered to discriminate the different taxa were: ≤4.70 mm for *N. anomalus*; 4.80–5.35 mm for *N. f. fodiens*; and >5.35 mm for *N. f. niethammeri*^19,23^. With the coordinates of the 16 landmarks, and using MorphoJ version 1.06d^52^, we made a Procrustes fit, calculated the centroid size, and performed a principal components analysis from the covariance matrix. Landmark coordinates of the mandibles used in this study are available in TPS format in Supplementary Appendix 1.

### DNA extraction, PCR amplification and sequencing

The photographed mandibles were then used for DNA extraction. They were first powdered using liquid nitrogen and a mortar after which genomic DNA was extracted with a QIAamp DNA Micro kit. In the case of tissues, DNA was extracted with a DNeasy Blood & Tissue Kit (QIAGEN) following the recommended protocol. Extractions of all degraded samples were performed in a separate room with UV irradiation to avoid contamination. Extraction blanks were always present in order to detect any possible contamination at each step. Additionally, pre-PCR procedures were developed in a dedicated UV-hood, in a controlled, sterile room.

Complete cytochrome *b* sequences (1140 bp) from *N. fodiens* samples were amplified in three fragments using already published primers^13^ with slight modifications to improve amplification (Supplementary Table S3). In addition, primers for a smaller fragment of 226 bp were developed with the purpose of amplifying DNA from the most degraded samples (Supplementary Table S3). The length amplified in each sample is shown in Supplementary Table S1.

For the nuclear markers, a set of six new primer pairs were designed starting from intron markers previously developed for *Neomys*^13^. The primers were placed in conserved intronic regions or exons that spanned small fragments (between 153 and 229 bp) and flanked the highest possible number of polymorphic sites. Some markers were amplified with more than one primer pair in various samples, as we reduced the amplified intron length during the work to enable the amplification of degraded samples that failed with the initial primers (Supplementary Table S4). PCR reactions were performed in a final volume of 25 µl with 2–6 µl genomic DNA, 0.15 µl of Promega GoTaq DNA polymerase, and 1 µM of each primer. The cycling conditions included an initial denaturation step of 30 s at 95 °C, followed by 40 cycles of denaturation (30 s at 95 °C), annealing (60 s at 54 °C for cytochrome *b* and 65 °C for introns), and extension (60 s at 72 °C), as well as a final extension of 5 min at 72 °C. For the most degraded skull samples, an alternative protocol using the QIAGEN Multiplex PCR was performed in a final volume of 50 µl with 4 µl of genomic DNA, 0.3 µM of each primer, and 25 µl of PCR Master Mix. In this case, there was an initial heat activation step of 15 min at 95 °C, the denaturation step was at 94 °C, the annealing temperature was lowered to 63 °C for the introns, and the annealing time was extended to 90 s. PCR products were purified using ExoSAP-It (Affymetrix) and sequenced at Macrogen Inc. Sequences were assembled using Geneious Pro 5.1.7 (https://www.geneious.com).

In introns where two or more variable sites were present in a sample, PHASE version 2.1.1^53^ with a threshold of 0.9 was used to phase the alleles. When the program did not produce results or length-heterozygous alleles were present, allele-specific primers were used to independently amplify the two alleles. Allele-specific primers consist of two primers that are identical to one another except for the last nucleotide, which is situated over a polymorphic position of the sequence to be phased. The use of this type of primers was suggested for the sequencing reaction^54^, although in this work we used them both for amplifying and sequencing. To design them, a polymorphic position was selected and two 19–20 nucleotide primers were synthetized, each of which had one of the two possible nucleotides placed at the 3′ end. Then, two independent PCR reactions were performed, one for each allele-specific primer, and using the opposite original primer at the other side of the polymorphic region for amplification. In this way, two PCR products, corresponding to the two alleles, were obtained. Finally, each PCR product was sequenced with the corresponding allele-specific primer to obtain the resolved partial allele, which was then assembled with the original PCR sequence to obtain the complete allele^54^. The primers used for PCR allele-specific resolution are shown in Supplementary Table S5.

### Phylogenetic analyses of cytochrome *b*

Since some cytochrome *b* sequences were completely amplified whereas others were only partial (Supplementary Table S1), they were aligned using MAFFT version 7.130^55^ with the *maxiterate* option enabled.

A Bayesian tree of the cytochrome *b* sequences was built using BEAST version 2.5.0^56^. To select a statistically appropriate model, a Bayes factors approach based on path sampling^57^ was used with 96 complete sequences. Markov chain Monte Carlo analyses were based on 100 steps of 10,000,000 generations sampled each 1,000 generations, 10% preburn-in, 10% burn-in, and an alpha parameter of the Beta distribution to divide steps of 0.3. The convergence of each tree was checked in Tracer v1.7.1, from the same software package. Different priors were tested for each parameter category, and a more complex model was selected only if it converged and improved the log marginal likelihood by more than 5, as recommended^57^. Using these criteria, the selected model was the following: HKY substitution model with estimated base frequencies and Gamma site heterogeneity for each partition (unlinked substitution models); a strict clock model for all partitions (linked clock model); and a coalescent constant size tree model. Once the best model had been selected, it was applied to the sequences of either all *Neomys* species or only *N. fodiens*. In these cases, 50,000,000 generations were run. Consensus trees were calculated using the TreeAnnotator program (BEAST2 package) with median heights.

For comparison, a maximum-likelihood tree of all sequences was reconstructed with RAxML version 8.0.19^58^ using a GTR substitution model (as recommended in the manual of this program), rate heterogeneity modeled with a Gamma distribution, and 100 randomized maximum-parsimony starting trees. Mid-point rooting was used to represent the tree.

### Multilocus analyses

The *Neomys* alleles showed some differences in length due to small indels. They were aligned with MAFFT version 7.130^55^ with the *maxiterate* option enabled. Then, a few gap positions as well as a few positions with unknown nucleotides were removed from each alignment. The six final alignments are available in FASTA format in Supplementary Appendix S2.

A maximum-likelihood tree was reconstructed with RAxML^58^ from each alignment as before. For each of these trees, haplotype genealogies were constructed using Haploviewer^59^.

In addition, we reconstructed a phylogenetic distance tree from the intron alignments, as in Igea *et al*.^13^. First, pairwise distances were calculated in such a way that the two alleles of each sequence were taken into account by making all possible comparisons between alleles^60^, and correcting for multiple substitutions using the Jukes-Cantor formula. The pairwise distance matrix was then utilized to construct a tree with the Fitch program of the Phylip software package^61^. Mid-point rooting was used to represent the tree.

### Genetic differentiation

Intron alignments were converted to a diploid multi-allelic data format by assigning a number to each allele. Then, pairwise F_ST_ for all populations was calculated with the R package *hierfstat* version 0.04–22^62^ using the *genet.dist* function with the WC84 method. Significance of the observed F_ST_ values was estimated by bootstrapping over loci using the *boot.ppfst* function of the same package with 100,000 replications and calculating the 95% confidence intervals. Significance was inferred if the confidence interval did not overlap zero.

### Genetic diversity

Nuclear genetic diversity (π and θ) was calculated using the Bioperl library PopGen version 1.6924^63^. The number of heterozygous positions in each sample were counted across the different nuclear markers and divided by their respective lengths. A map of heterozygosity values was then represented with QGIS^64^.

## Supplementary information

Supplementary Information: Figures and Tables

Appendix S1

Appendix S2

## Data Availability

A total of 611 new sequences were deposited in EMBL/GenBank with Accession Numbers LR585354-LR585964, using the European Nucleotide Archive. For introns amplified using various primer pairs, only the shortest sequence was deposited for all samples.
