# Publisher Correction: Size increase without genetic divergence in the Eurasian water shrew *Neomys fodiens*

**DOI:** 10.1038/s41598-020-58289-9

**Published:** 2020-02-05

**Authors:** Alfonso Balmori-de la Puente, Carlos Nores, Jacinto Román, Angel Fernández-González, Pere Aymerich, Joaquim Gosálbez, Lídia Escoda, Jose Castresana

**Affiliations:** 1 Institute of Evolutionary Biology (CSIC-Universitat Pompeu Fabra), Passeig Marítim de la Barceloneta 37, 08003 Barcelona, Spain; 2 Indurot, Universidad de Oviedo, Campus de Mieres, 33600 Mieres, Asturias Spain; 3 Department of Conservation Biology, Doñana Biological Station, CSIC, Calle Americo Vespucio 26, 41092 Sevilla, Spain; 4 Biosfera Consultoría Medioambiental S.L., Calle Candamo 5, 33012 Oviedo, Spain; 5 Calle Barcelona 29, 08600 Berga Barcelona, Spain; 6 Department of Evolutionary Biology, Ecology and Environmental Sciences, University of Barcelona, Avinguda Diagonal 645, 08028 Barcelona, Spain

Correction to: *Scientific Reports* 10.1038/s41598-019-53891-y, published online 22 November 2019

Two supplementary files, Appendix S1 and Appendix S2, were omitted from the original version of this Article. This has been corrected in the HTML version of this article; the PDF version was correct at time of publication.

