# Supplementary material for: Size increase without genetic divergence in the Eurasian water shrew Neomys fodiens
Source: Sci Rep. 2019 Nov 22;9:17375. doi: 10.1038/s41598-019-53891-y (PMC6874603; doi:10.1038/s41598-019-53891-y)
Supplement: Supplementary file 1 — Supplementary Information: Figures and Tables [file 41598_2019_53891_MOESM1_ESM.pdf]

Supplementary Information: Figures and Tables

Size increase without genetic divergence in the Eurasian water shrew *Neomys fodiens*

Alfonso Balmori-de la Puente [1], Carlos Nores [2], Jacinto Román [3], Angel Fernández-González [4], Pere Aymerich [5], Joaquim Gosálbez [6], Lídia Escoda [1], Jose Castresana [1]

[1] Institute of Evolutionary Biology (CSIC-Universitat Pompeu Fabra), Passeig Marítim de la Barceloneta 37, 08003 Barcelona, Spain

[2] Indurot, Universidad de Oviedo, Campus de Mieres, 33600 Mieres, Asturias, Spain

[3] Department of Conservation Biology, Doñana Biological Station, CSIC, Calle Americo Vespucio 26, 41092 Sevilla, Spain

[4] Biosfera Consultoría Medioambiental S.L., Calle Candamo 5, 33012 Oviedo, Spain

[5] Calle Barcelona 29, 08600 Berga, Barcelona, Spain

[6] Department of Evolutionary Biology, Ecology and Environmental Sciences, University of Barcelona, Avinguda Diagonal 645, 08028 Barcelona, Spain

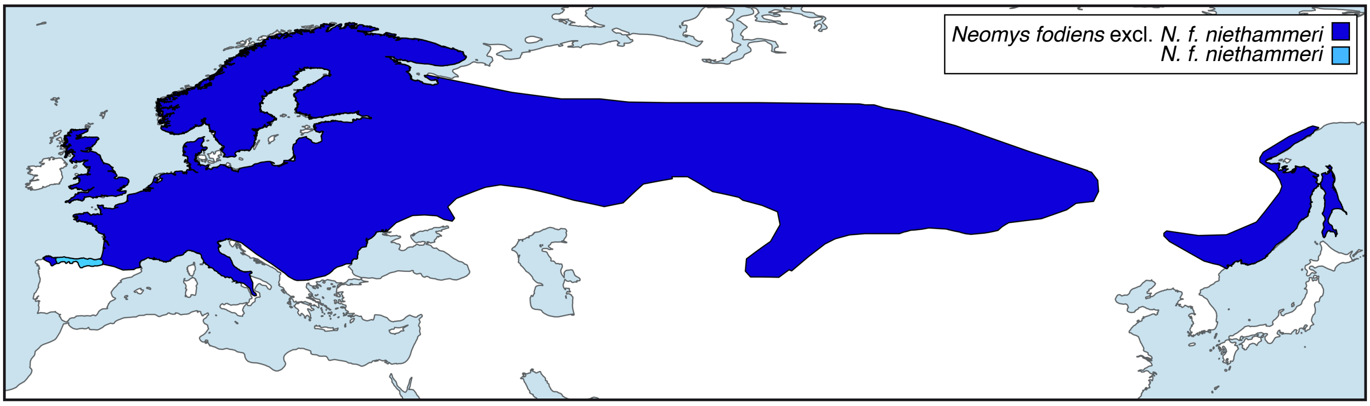

Figure S1. Map showing the range of *Neomys fodiens niethammeri* (light blue) within the range of *N. fodiens*. The range of *N. fodiens* not corresponding to *N. f. niethammeri* may include several subspecies whose ranges are not clearly delimited.

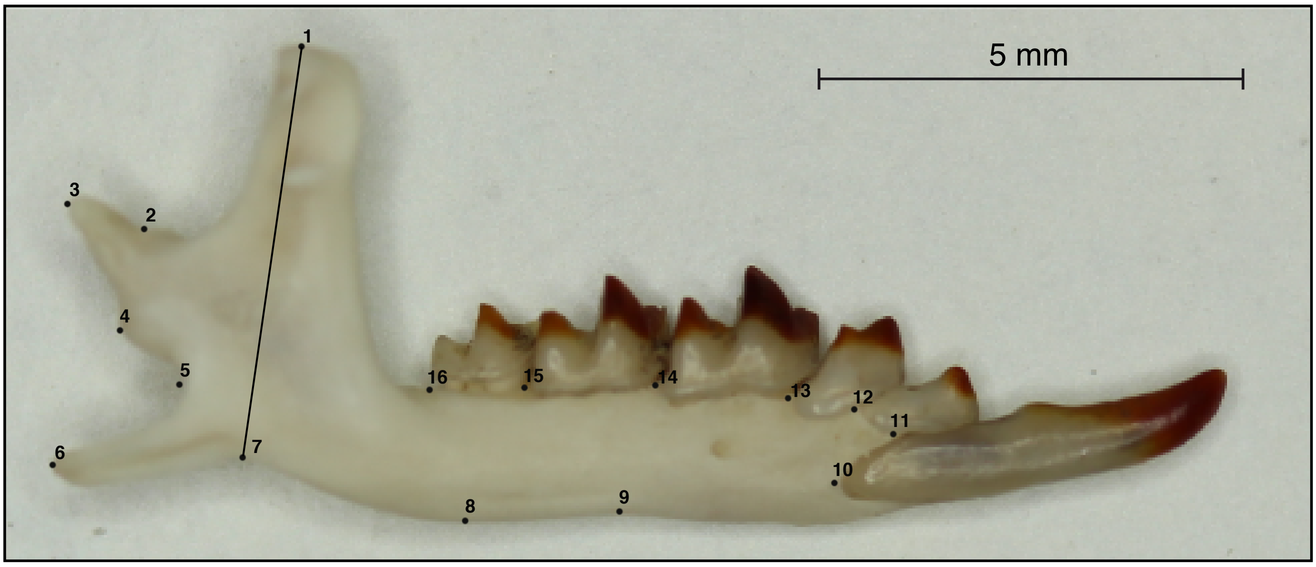

Figure S2. Landmarks selected in this study in order to estimate the centroid size and perform principal components analysis. Landmarks 1 and 7 were used to calculate the height of the coronoid process, as shown with the line.

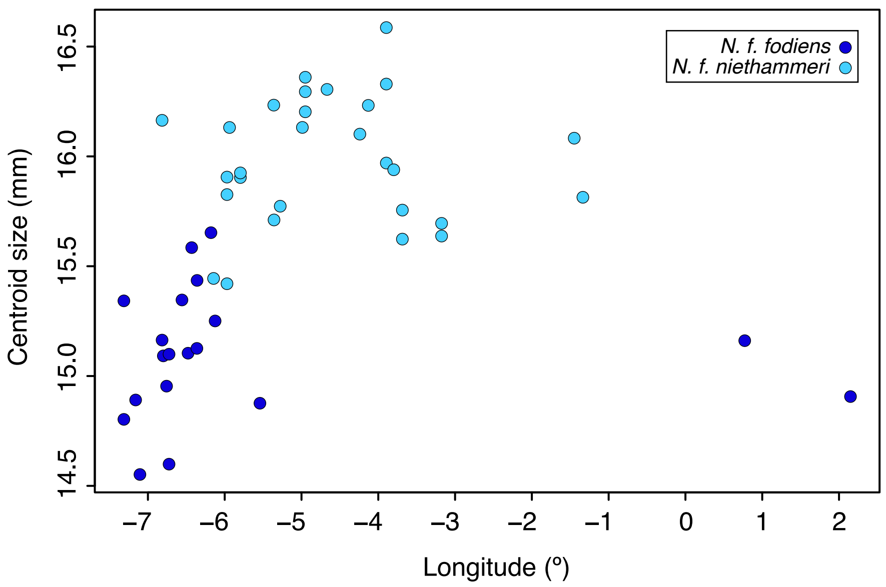

Figure S3. Plot across longitude showing differences in skull size of *N. fodiens* subspecies found in the Iberian Peninsula, as measured with the centroid size of the 16 landmarks selected.

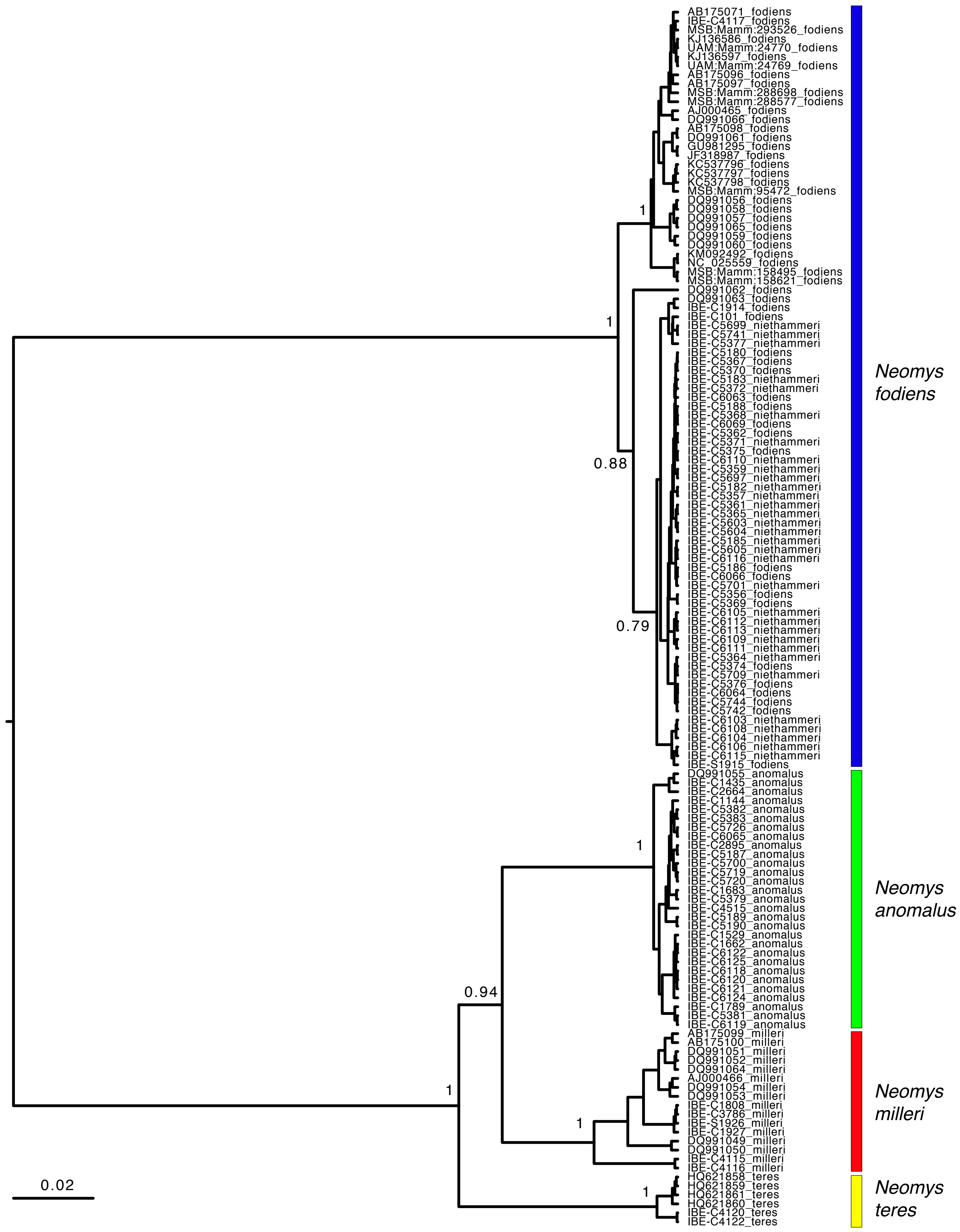

Figure S4. Bayesian phylogenetic tree of sequences obtained in this work plus sequences from Igea et al. (2015) and sequences downloaded from GenBank. The scale is in substitutions per position. Posterior probabilities are indicated for the species and the clades mentioned in the text.

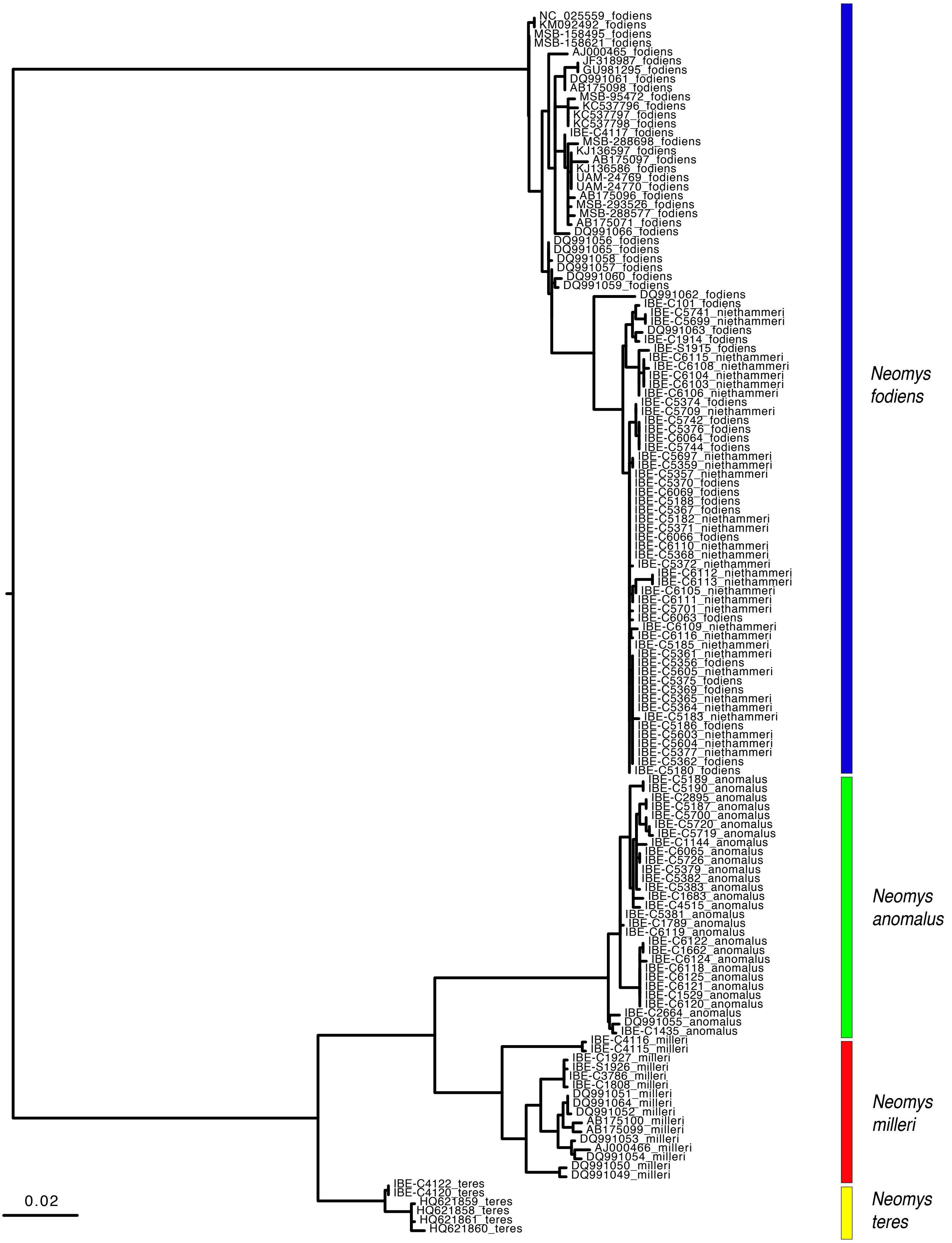

Figure S5. RAxML phylogenetic tree of sequences obtained in this work plus sequences from Igea et al. (2015) and sequences downloaded from GenBank, performed with 100 starting trees. The scale is in substitutions per position.

Table S1. Samples used in this study with information about sample type, collection year, locality data, morphological data (marked with XX for geometric morphometrics and X for coronoid height), cytochrome *b* length (being 226 bp the length of the Neo 1.2 fragment; 569 the length of the Neo 1.2 - Neo 2 combined fragments, which have an overlapping region; and 1140 the length of the three overlapping fragments), and number of sequenced introns in samples with nuclear information. Samples from the collection of the Department of Organisms and Systems Biology of the University of Oviedo are indicated with (UO). Samples from the Museum of Southwestern Biology (MSB) and the University of Alaska Museum (UAM) are indicated with their respective codes. The sample IBE-C4117 (2200-Briansk) is from the collection of A. Bannikova. Samples of Igea et al. (2015) are also included and indicated with (1).

Specimen Code	Species	Sample type	Year	Lat.	Lon.	Locality	Administrative Division / Country	Morphology	Cyt *b* length (bp)	Nº of introns
IBE-C5180 (UO)	*N. fodiens*	Skull	1979	43.4	-6.8	Boal	Asturias	XX	226	-
IBE-C5182 (UO)	*N. fodiens*	Skull	1970	43.4	-4.7	Vidiago	Asturias	XX	1140	6
IBE-C5183 (UO)	*N. fodiens*	Skull	1980	43.3	-5.9	Peñerudes	Asturias	XX	569	-
IBE-C5185 (UO)	*N. fodiens*	Skull	1970	43.5	-5	Cuerres	Asturias	XX	569	-
IBE-C5186 (UO)	*N. fodiens*	Skull	1979	43.4	-6.1	Rebolleda	Asturias	XX	569	-
IBE-C5188 (UO)	*N. fodiens*	Skull	1981	43.5	-5.5	Peón	Asturias	XX	1140	5
IBE-C5356 (UO)	*N. fodiens*	Skull	1980	43.3	-6.5	Obona	Asturias	XX	226	-
IBE-C5357 (UO)	*N. fodiens*	Skull	1980	43.3	-4.2	Cabezón de la Sal	Cantabria	XX	1140	-
IBE-C5359 (UO)	*N. fodiens*	Skull	1980	43.2	-5.8	Valdecuna	Asturias	XX	1140	4
IBE-C5361 (UO)	*N. fodiens*	Skull	1978	43.4	-5.4	Infiesto	Asturias	XX	226	-
IBE-C5362 (UO)	*N. fodiens*	Skull	1980	43.4	-6.4	La Millariega	Asturias	XX	226	-
IBE-C5364 (UO)	*N. fodiens*	Skull	1980	43.4	-4.1	Quijas	Cantabria	XX	226	-
IBE-C5365 (UO)	*N. fodiens*	Skull	1981	43.2	-5.3	La Foz	Asturias	XX	226	-
IBE-C5367 (UO)	*N. fodiens*	Skull	1979	43.6	-6.4	Ribón	Asturias	XX	1140	6
IBE-C5368 (UO)	*N. fodiens*	Skull	1988	43.4	-6.8	Boal	Asturias	XX	1140	6
IBE-C5369 (UO)	*N. fodiens*	Skull	1986	43.3	-6.2	Restiello	Asturias	XX	226	-
IBE-C5370 (UO)	*N. fodiens*	Skull	1983	43.4	-6.8	La Felechosa	Asturias	XX	1140	6
IBE-C5371 (UO)	*N. fodiens*	Skull	1980	43.6	-6.2	Piñera	Asturias	XX	1140	5
IBE-C5372 (UO)	*N. fodiens*	Skull	1982	43.5	-5.4	Priesca	Asturias	XX	1140	6
IBE-C5374 (UO)	*N. fodiens*	Skull	1979	43.4	-7.1	Pueblonuevo	Asturias	XX	1140	6
IBE-C5375 (UO)	*N. fodiens*	Skull	1982	42.9	-6.4	Palacios del Sil	León	XX	226	-
IBE-C5376 (UO)	*N. fodiens*	Skull	1978	43.4	-7.2	Conforto	Lugo	XX	1140	4
IBE-C5377 (UO)	*N. fodiens*	Skull	1980	43.2	-5.8	Valdecuna	Asturias	XX	226	-
IBE-C5603 (UO)	*N. fodiens*	Skull	2015	43.3	-5	Avín	Asturias	XX	1140	6
IBE-C5604 (UO)	*N. fodiens*	Skull	2015	43.3	-5	Avín	Asturias	XX	1140	6
IBE-C5605 (UO)	*N. fodiens*	Skull	2015	43.3	-5	Avín	Asturias	XX	226	-
IBE-C5697	*N. fodiens*	Skull	2017	43.1	-6	San Vicente de Nimbra	Asturias	XX	1140	6
IBE-C5699	*N. fodiens*	Skull	2017	42.7	-1.5	Iglesia Zuazu	Navarra	XX	1140	5
IBE-C5701	*N. fodiens*	Skull	2017	43.1	-6	San Vicente de Nimbra	Asturias	XX	1140	6
IBE-C5709	*N. fodiens*	Skull	2017	43.1	-6	San Vicente de Nimbra	Asturias	XX	1140	-
IBE-C5741	*N. fodiens*	Skull	2017	43	-1.3	Iglesia Burguete	Navarra	XX	1140	5
IBE-C5742	*N. fodiens*	Skull	2017	42.7	-7.3	San Martiño	Lugo	XX	1140	6
IBE-C5744	*N. fodiens*	Skull	2017	43.2	-6.8	La Figuerina	Asturias	XX	1140	6
IBE-C6063	*N. fodiens*	Skull	2017	43.3	-6.7	San Pedro de Lago	Asturias	XX	1140	6
IBE-C6064	*N. fodiens*	Skull	2017	42.7	-7.3	San Martiño	Lugo	XX	1140	4
IBE-C6066	*N. fodiens*	Skull	2017	43.2	-6.6	Santa Marina de Obanca	Asturias	XX	1140	5
IBE-C6069	*N. fodiens*	Skull	2017	43.3	-6.7	San Pedro de Lago	Asturias	XX	1140	6
IBE-C6103	*N. fodiens*	Skull	1984	42.8	-3.7	Cubillo de Butrón	Burgos	XX	569	-
IBE-C6104	*N. fodiens*	Skull	1984	42.8	-3.7	Cubillo de Butrón	Burgos	XX	569	-
IBE-C6105	*N. fodiens*	Skull	1985	43.1	-3.3	Caniego	Burgos	X	1140	5
IBE-C6106	*N. fodiens*	Skull	1985	43.1	-3.3	Caniego	Burgos	X	1140	5
IBE-C6108	*N. fodiens*	Skull	1985	43.1	-3.6	Hornillalatorre	Burgos	X	1140	5
IBE-C6109	*N. fodiens*	Skull	1986	43.2	-3.9	Entrambasmestas	Cantabria	XX	569	-
IBE-C6110	*N. fodiens*	Skull	1986	43.2	-3.9	Entrambasmestas	Cantabria	XX	1140	5
IBE-C6111	*N. fodiens*	Skull	1986	43.2	-3.9	Entrambasmestas	Cantabria	XX	1140	-
IBE-C6112	*N. fodiens*	Skull	1986	43	-3.2	Encima Angulo	Burgos	XX	226	-
IBE-C6113	*N. fodiens*	Skull	1986	43	-3.2	Encima Angulo	Burgos	XX	226	-
IBE-C6115	*N. fodiens*	Skull	1987	42.8	-3.2	Orbañanos	Burgos	X	226	-
IBE-C6116	*N. fodiens*	Skull	1993	42.7	-3.8	Covanera	Burgos	XX	1140	5
IBE-C4117 (2200-Briansk)	*N. fodiens*	Tissue		53.3	34.4	Briansk	Russia		1140	6
MSB:Mamm:95472	*N. fodiens*	Tissue	1997	47.7	17.4	Feher-to	Hungary		1140	6
MSB:Mamm:158495	*N. fodiens*	Tissue	2006	43.2	84.3	Narati	China		1140	6
MSB:Mamm:158621	*N. fodiens*	Tissue	2006	43.2	84.3	Narati	China		1140	6
MSB:Mamm:288577	*N. fodiens*	Tissue	2015	48.2	89	Songinot Gol	Mongolia		1140	6
MSB:Mamm:288698	*N. fodiens*	Tissue	2015	49.5	94.7	Han Huhnii Mountain	Mongolia		1140	6
MSB:Mamm:293526	*N. fodiens*	Tissue	2016	49	103.2	Tsachirt River	Mongolia		1140	6

UAM:Mamm:24769	*N. fodiens*	Tissue	1986	60.8	24.5	Loppi	Finland		1140	6
UAM:Mamm:24770	*N. fodiens*	Tissue	1986	60.8	24.5	Loppi	Finland		1140	5
IBE-C5187	*N. anomalus*	Skull	1980	43.2	-5.5	Rioseco	Asturias	XX	569	-
IBE-C5189	*N. anomalus*	Skull	1979	43.5	-6.1	Corias	Asturias	XX	226	-
IBE-C5190	*N. anomalus*	Skull	1980	43.5	-6.4	Muñás	Asturias	XX	1140	-
IBE-C5379	*N. anomalus*	Skull	1978	43.4	-5.4	Infiesto	Asturias	X	226	-
IBE-C5381	*N. anomalus*	Skull	1980	42.8	-3.9	Valderredible	Cantabria	XX	226	-
IBE-C5382	*N. anomalus*	Skull	1983	42.6	-6.2	Manzanal del Puerto	León	XX	226	-
IBE-C5383	*N. anomalus*	Skull	1984	43.5	-8.2	Ferrol	La Coruña	X	1140	6
IBE-C5700	*N. anomalus*	Skull	2017	43.1	-6	San Vicente de Nimbra	Asturias	XX	1140	6
IBE-C5719	*N. anomalus*	Skull	2017	43.2	-6.6	Santa Marina de Obanca	Asturias	XX	1140	6
IBE-C5720	*N. anomalus*	Skull	2017	43.2	-6.6	Santa Marina de Obanca	Asturias	XX	1140	6
IBE-C5726	*N. anomalus*	Skull	2017	43.2	-6.8	Convento de San Bruno	Asturias	XX	1140	6
IBE-C6065	*N. anomalus*	Skull	2017	43.2	-6.8	Convento de San Bruno	Asturias	XX	1140	6
IBE-C6118	*N. anomalus*	Skull	1986	42.8	-3.2	Herrán	Burgos		1140	5
IBE-C6119	*N. anomalus*	Skull	1984	42.8	-3.7	Cubillo de Butrón	Burgos	XX	226	-
IBE-C6120	*N. anomalus*	Skull	1987	43	-3.9	Cilleruelo de Bezana	Burgos	XX	569	-
IBE-C6121	*N. anomalus*	Skull	1991	43	-4.2	Barrio	Cantabria	XX	1140	5
IBE-C6122	*N. anomalus*	Skull	1986	43	-3.2	Encima Angulo	Burgos		569	-
IBE-C6124	*N. anomalus*	Skull	1986	43.2	-3.9	Entrambasmestas	Cantabria	XX	1140	5
IBE-C6125	*N. anomalus*	Skull	2015	43.1	-3.3	Caniego	Burgos		1140	4
IBE-C4515	*N. anomalus*	Tissue	2015	43.4	-6.4	La Millariega	Asturias		1140	6
IBE-C1927	*N. milleri*	Tissue	2012	42.6	1.1	Escalarre	Lleida		1140	6
IBE-C101	*N. fodiens*	(1)	2007	42.4	2.2	Queralbs	Barcelona	XX	1140	6
IBE-C1914	*N. fodiens*	(1)	2010	42.5	0.8	Coll	Lleida	XX	1140	6
IBE-S1915	*N. fodiens*	(1)	2009	42.9	-2.4	Zalduondo	Alava		1140	-
IBE-C1144	*N. anomalus*	(1)	2010	43.1	-6.7	Vega de Horreo	Asturias		1140	-
IBE-C1435	*N. anomalus*	(1)	2010	41.7	-5	Penaflor de Hornija	Valladolid		1140	-
IBE-C1529	*N. anomalus*	(1)	2010	43.3	-4.8	Tielve	Asturias		1140	6
IBE-C1662	*N. anomalus*	(1)	2010	42	-2.6	Molinos de Razón	Soria		1140	-
IBE-C1683	*N. anomalus*	(1)	2010	42.2	-6.7	Trefacio	Zamora		1140	-
IBE-C1789	*N. anomalus*	(1)	2008	40.3	-5.5	Navalguijo	Avila		1140	6
IBE-C2664	*N. anomalus*	(1)	2011	40.7	-0.2	Bergantes	Castellón		1140	-
IBE-C2895	*N. anomalus*	(1)		43.2	-4.9	Picos de Europa	León		1140	6
IBE-C1808	*N. milleri*	(1)	2010	42.3	1	La Pobla de Segur	Lleida		1140	6
IBE-C3786	*N. milleri*	(1)	2012	42.3	1.9	Guardiola de Bergueda	Barcelona		1140	6
IBE-C4115	*N. milleri*	(1)	2000	55.2	30.2	Vitebsk	Belarus		1140	-
IBE-C4116	*N. milleri*	(1)	2007	50.6	36.6	Belgorod	Russia		1140	6
IBE-S1926	*N. milleri*	(1)	2009	41.9	2.5	Osor	Girona		1140	-
IBE-C4120	*N. teres*	(1)	1999	43.9	40.1	North Caucasus	Russia		1140	6
IBE-C4122	*N. teres*	(1)	1999	43.9	40.1	North Caucasus	Russia		1140	6

Table S2. Measurements of coronoid height and centroid size of the mandibles. For broken mandibles, only coronoid height could be measured.

Specimen code	Taxon	Coronoid height (mm)	Centroid size (mm)
IBE-C5180	*N. f. fodiens*	5.24	15.2
IBE-C5182	*N. f. niethammeri*	5.76	16.3
IBE-C5183	*N. f. niethammeri*	5.76	16.1
IBE-C5185	*N. f. niethammeri*	5.87	16.1
IBE-C5186	*N. f. fodiens*	5.2	15.3
IBE-C5188	*N. f. fodiens*	5.25	14.9
IBE-C5356	*N. f. fodiens*	5.16	15.1
IBE-C5357	*N. f. niethammeri*	5.65	16.1
IBE-C5359	*N. f. niethammeri*	5.5	15.9
IBE-C5361	*N. f. niethammeri*	5.84	16.2
IBE-C5362	*N. f. fodiens*	5.3	15.4
IBE-C5364	*N. f. niethammeri*	5.6	16.2
IBE-C5365	*N. f. niethammeri*	5.72	15.8
IBE-C5367	*N. f. fodiens*	5.26	15.1
IBE-C5368	*N. f. niethammeri*	5.37	16.2
IBE-C5369	*N. f. fodiens*	5.31	15.7
IBE-C5370	*N. f. fodiens*	5.18	15.0
IBE-C5371	*N. f. niethammeri*	5.52	15.4
IBE-C5372	*N. f. niethammeri*	5.4	15.7
IBE-C5374	*N. f. fodiens*	4.9	14.6
IBE-C5375	*N. f. fodiens*	5.2	15.6
IBE-C5376	*N. f. fodiens*	5.17	14.9
IBE-C5377	*N. f. niethammeri*	5.53	15.9
IBE-C5603	*N. f. niethammeri*	5.84	16.2
IBE-C5604	*N. f. niethammeri*	5.93	16.3
IBE-C5605	*N. f. niethammeri*	5.72	16.4
IBE-C5697	*N. f. niethammeri*	5.49	15.9
IBE-C5699	*N. f. niethammeri*	5.4	16.1
IBE-C5701	*N. f. niethammeri*	5.82	15.8
IBE-C5709	*N. f. niethammeri*	5.67	15.4
IBE-C5741	*N. f. niethammeri*	5.43	15.8
IBE-C5742	*N. f. fodiens*	5.06	15.3
IBE-C5744	*N. f. fodiens*	5.24	15.1
IBE-C6063	*N. f. fodiens*	5.08	15.1
IBE-C6064	*N. f. fodiens*	5.08	14.8
IBE-C6066	*N. f. fodiens*	5.11	15.3
IBE-C6069	*N. f. fodiens*	5.2	14.6
IBE-C6103	*N. f. niethammeri*	5.68	15.6
IBE-C6104	*N. f. niethammeri*	5.72	15.8
IBE-C6105	*N. f. niethammeri*	5.84	
IBE-C6106	*N. f. niethammeri*	5.99	
IBE-C6108	*N. f. niethammeri*	5.62	
IBE-C6109	*N. f. niethammeri*	5.82	16.3
IBE-C6110	*N. f. niethammeri*	5.54	16.0
IBE-C6111	*N. f. niethammeri*	5.81	16.6

IBE-C6112	*N. f. niethammeri*	5.6	15.7
IBE-C6113	*N. f. niethammeri*	5.48	15.6
IBE-C6115	*N. f. niethammeri*	5.43	
IBE-C6116	*N. f. niethammeri*	5.74	15.9
IBE-C5187	*N. anomalus*	4.28	13.9
IBE-C5189	*N. anomalus*	4.66	14.5
IBE-C5190	*N. anomalus*	4.36	14.1
IBE-C5379	*N. anomalus*	4.27	
IBE-C5381	*N. anomalus*	4.55	15.0
IBE-C5382	*N. anomalus*	4.52	14.5
IBE-C5383	*N. anomalus*	4.62	
IBE-C5700	*N. anomalus*	4.31	14.3
IBE-C5719	*N. anomalus*	4.36	13.9
IBE-C5720	*N. anomalus*	4.22	14.1
IBE-C5726	*N. anomalus*	4.3	14.4
IBE-C6065	*N. anomalus*	4.29	14.2
IBE-C6119	*N. anomalus*	4.44	14.7
IBE-C6120	*N. anomalus*	4.48	14.4
IBE-C6121	*N. anomalus*	4.37	14.3
IBE-C6124	*N. anomalus*	4.53	14.5
IBE-C101	*N. f. fodiens*	4.95	14.9
IBE-C1914	*N. f. fodiens*	4.92	15.2

Table S3. Primers used in this work for the amplification of the different overlapping fragments of the cytochrome *b* and the special fragment employed in highly degraded samples (Neo 1.2). Fragment Neo 1.2 allowed the amplification of 26 % of the mandibles used in this study, whereas only 14 % of the available mandibles tested remained undetermined. Primer temperature (T) and length of the amplified sequences without counting primers are also shown. Newly designed primers or modified from a previous published work (Igea et al., 2015) are marked with an asterisk. For fresh tissue or recent skull samples the whole cytochrome *b* sequence could be obtained in a single PCR reaction using flanking primers (1).

Fragment	Name	Primer sequence	T (ºC)	Length (bp)
Neo1	Neomys_tRNAGlu (1)	ATCGTTGTTATTCAACTATAAGAAC	64	404
	Neomys_cytb _R1.2 *	CCYCARAATGATATTTGYCCTCA	65	
Neo2	Neomys_cytb _F2.3 *	CAGTTATAGCYACTGCCTTTATA	63	358
	Neomys_cytb _R2.1 *	AATTRTCYGGGTCTCCGAGTA	62	
Neo3	Neomys_cytb_614F	TWTTCCTYCATGAAACAGGATC	61	544
	Neomys_tRNAThr (1)	TTTTGGTTTACAAGACCAGTGTAT	64	
Neo1.2	Neomys_cytb_F1.1 *	TAGCAATACATTAYACTTCMGACAC	68	226
	Neomys_cytb_403R	YCCYCARAATGATATTTGYCCTCA	68	

Table S4. Primer pairs used to sequence intron fragments in *Neomys* species. For some introns, more than one pair was designed and the final established set is shown with an asterisk. Amplification of degraded samples with shorter fragments allowed a gain of 12 % of amplified loci. Primer temperature (T), amplified length without counting primer sequence, and number of samples sequenced with each pair are shown. Primer design tried to homogenize hybridization temperature of the different markers, which was set to 65º in conventional PCR reactions and 63º when using Multiplex, as higher temperatures were not recommended in this protocol.

Nuclear marker	Name	Primer sequence	T (ºC)	Length (bp)	Samples sequenced
ASB6-2	ASB6-2-F2	TCCTGGGTGATAACAAAGGCCT *	66	215	54
	ASB6-2-R2	CAGGTGCGTCAGTGCCTGCT *	66		
CSF2-2	CSF2-2-F2	GGCACTGGTGGTAGTCAGTGA *	66	229	57
	CSF2-2-R2	TGGACATGAGTCTGAATCTTGCT *	66		
GDAP1-1	GDAP1-F2	CAGCACCAGGACAGCTCCCA	66	229	8
	GDAP1-R2	ATGCTAAAGGAGGTAACTGTGGA	66		
	GDAP1-F3	ACCAGGACAGCTCCCAGGGT *	66	198	44
	GDAP1-R3	GCGTTTCTCTCTGCATCTCTCT *	66		
JMJD-2	JMJD2-F2	TGCGGTCAGCGGCCATCTCA	66	225	10
	JMJD2-R2	CAAGGTTCCTGCTGGCCAGCT	68		
	JMJD2-F3	CTCCACCCTGGYAGAACCCT *	65	153	41
	JMJD2-R3	TCACAGCACCTGCTCTGTGCT *	66		
MYCBPAP-11	MYCBPAP-F2	CAGTTTTATTTTGACAACCGGGAA	66	344	8
	MYCBPAP-R2	CGTYTCTTCAGGCAGAATCACA	65		
	MYCBPAP-F3	CCCAGGGAGCAGGAGCATTCT	68	200	6
	MYCBPAP-R2	CGTYTCTTCAGGCAGAATCACA	65		
	MYCBPAP-F5	ATTCTAGGGGTTYAGCTGCTTCT *	67	182	43
	MYCBPAP-R2	CGTYTCTTCAGGCAGAATCACA *	65		
TRAIP-8	TRAIP-F3	GCTCCTGGCTCCTAGACATCT *	66	198	55
	TRAIP-R3	CCCAGGCTGAACTCTCCACAA *	66		

Table S5. Allele-specific primers used for amplification and sequencing of nuclear introns. Primer temperature (T) and polymorphic position of the sequence (SNP position) are also shown. PCRs were performed with an annealing temperature of 65º or 63º with conventional or Multiplex reactions, respectively. 14 % of samples were phased for MYCBPAP-11 introns using these primers.

Primer name	Primer sequence	T (ºC)	SNP position
MYCBPAP-F5_A	TGGGCCCGGGGCTCCCTTA	66	123
MYCBPAP-F5_T	TGGGCCCGGGGCTCCCTTT	66	123
MYCBPAP-R2_C	GGCAGAAGCCAGCTGAGGCC	68	134
MYCBPAP-R2_G	GGCAGAAGCCAGCTGAGGCG	68	134

Table S6. Genetic diversity parameters (π and θ) of the different introns estimated for all *N. fodiens* samples and the two different populations considered.

	All		Iberian		Eurasian	
	π	θ	π	θ	π	θ
ASB6-2	0.0009	0.0034	0.0010	0.0027	0.0005	0.0011
CSF2-2	0.0002	0.0009	0.0003	0.0009	0.0000	0.0000
GDAP1-1	0.0023	0.0010	0.0024	0.0010	0.0021	0.0013
JMJD-2	0.0054	0.0050	0.0036	0.0040	0.0069	0.0032
MYCBPAP-11	0.0055	0.0040	0.0058	0.0032	0.0006	0.0014
TRAIP-8	0.0004	0.0019	0.0002	0.0010	0.0010	0.0012
Nuclear average	0.0025	0.0027	0.0022	0.0021	0.0019	0.0014
