# Supplementary material for: Size increase without genetic divergence in the Eurasian water shrew Neomys fodiens
Source: Sci Rep. 2019 Nov 22;9:17375. doi: 10.1038/s41598-019-53891-y (PMC6874603; doi:10.1038/s41598-019-53891-y)
Supplement: Supplementary file 2 — Appendix S1 [file 41598_2019_53891_MOESM2_ESM.pdf]

Appendix S1. Landmark coordinates of the mandibles used in this study in TPS format.

Size increase without genetic divergence in the Eurasian water shrew *Neomys fodiens*

Alfonso Balmori-de la Puente [1], Carlos Nores [2], Jacinto Román [3], Angel Fernández-González [4], Pere Aymerich [5], Joaquim Gosálbez [6], Lídia Escoda [1], Jose Castresana [1]

[1] Institute of Evolutionary Biology (CSIC-Universitat Pompeu Fabra), Passeig Marítim de la Barceloneta 37, 08003 Barcelona, Spain

[2] Indurot, Universidad de Oviedo, Campus de Mieres, 33600 Mieres, Asturias, Spain

[3] Department of Conservation Biology, Doñana Biological Station, CSIC, Calle Americo Vespucio 26, 41092 Sevilla, Spain

[4] Biosfera Consultoría Medioambiental S.L., Calle Candamo 5, 33012 Oviedo, Spain

[5] Calle Barcelona 29, 08600 Berga, Barcelona, Spain

[6] Department of Evolutionary Biology, Ecology and Environmental Sciences, University of Barcelona, Avinguda Diagonal 645, 08028 Barcelona, Spain

LM= 16
5.329 11.342
3.097 9.166
2.195 9.495
2.834 7.869
3.642 7.173
2.091 6.083
4.140 6.243
6.706 5.378
8.529 5.406
11.227 5.764
12.157 6.450
11.800 6.647
10.851 7.051
9.234 7.211
7.721 7.183
6.565 7.079
ID= C5180_fodiens

LM= 16
4.212 12.627
2.208 10.266
1.202 10.680
1.493 8.986
2.500 8.055
0.684 7.124
2.970 7.001
6.028 5.901
8.154 5.929
10.571 6.446
11.879 7.284
11.061 7.632
10.026 7.754
8.210 7.905
6.639 7.829
5.492 7.905
ID= C5182_niethammeri

LM= 16
4.774 12.114
3.041 9.327
2.006 9.619
2.646 8.018
3.682 7.293
2.194 5.956
4.379 6.370
7.269 5.796
9.557 6.050
11.949 6.822
12.797 7.651
12.185 7.858
11.193 7.860
9.520 7.924
8.037 7.740
6.827 7.566
ID= C5183_niethammeri

LM= 16
4.650 11.419
2.610 8.712
1.652 9.173
2.197 7.397
3.080 6.729
1.389 5.470
3.550 5.658
6.689 4.718
8.494 4.944
11.398 5.498
12.112 6.344
11.586 6.560
10.637 6.767
8.992 6.861
7.357 6.682
6.266 6.720
ID= C5185_niethammeri

LM= 16
4.098 11.964
2.209 9.756
1.250 10.169
1.711 8.440
2.519 7.801
0.771 6.842
3.224 6.833
6.109 6.006
7.820 6.147
10.432 6.664
11.062 7.265
10.667 7.556
9.859 7.857
8.233 7.970
6.748 7.923
5.630 7.848
ID= C5186_fodiens

LM= 16
4.218 11.633
2.422 9.171
1.473 9.528
2.009 8.015
2.845 7.357
1.229 6.238
3.428 6.445
6.257 5.618
7.892 5.684
10.458 6.135
11.219 6.887
10.731 7.169
9.838 7.347
8.268 7.441
6.605 7.413
5.656 7.357
ID= C5188_fodiens

LM= 16
3.912 13.065
2.305 10.806
1.361 11.156
1.809 9.540
2.821 8.829
1.088 7.612
3.552 7.914
6.278 7.125
8.040 7.203
10.475 7.612
11.205 8.303
10.689 8.537
9.832 8.683
8.235 8.800
6.755 8.761
5.723 8.829
ID= C5356_fodiens

LM= 16
4.104 12.313
1.939 9.711
1.085 9.954
1.677 8.303
2.609 7.624
1.007 6.517
3.201 6.731
6.405 6.206
8.133 6.323
10.900 7.012
11.930 7.828
11.124 8.012
10.143 8.362
8.648 8.187
7.095 8.216
5.900 8.119
ID= C5357_niethammeri

LM= 16
3.661 12.247
1.669 9.614
0.765 9.886
1.416 8.331
2.456 7.592
0.668 6.280
3.273 6.757
5.595 6.203
8.005 6.475
10.474 7.155
11.348 7.981
10.940 8.195
9.949 8.399
8.200 8.302
6.606 8.039
5.411 7.884
ID= C5359_niethammeri

LM= 16
3.660 12.452
2.362 9.729
1.228 10.107
1.887 8.411
3.060 7.674
1.170 6.269
3.796 6.618
6.441 6.085
8.728 6.579
11.344 7.209
11.994 8.052
11.354 8.246
10.521 8.440
8.738 8.304
7.255 8.188
5.996 8.014
ID= C5361_niethammeri

LM= 16
4.801 13.065
2.978 10.686
1.853 11.268
2.270 9.571
3.055 8.902
1.222 7.932
3.666 7.893
6.450 6.855
8.632 6.991
10.815 7.262
11.891 7.893
11.241 8.135
10.359 8.407
8.739 8.533
7.090 8.591
5.955 8.591
ID= C5362_fodiens

LM= 16
4.074 13.589
2.432 11.060
1.394 11.409
1.928 9.721
2.927 8.974
1.181 7.820
3.586 8.014
6.642 7.034
8.397 7.073
11.113 7.510
11.928 8.305
11.385 8.538
10.609 8.761
8.795 8.780
7.165 8.809
5.934 8.819
ID= C5364_niethammeri

LM= 16
4.835 13.187
2.796 10.604
1.748 10.973
2.398 9.303
3.262 8.556
1.621 7.459
3.801 7.566
6.699 6.672
8.922 6.925
11.194 7.420
12.097 8.197
11.631 8.459
10.728 8.604
8.845 8.653
7.437 8.546
6.427 8.381
ID= C5365_niethammeri

LM= 16
5.407 13.220
3.642 10.834
2.585 11.261
3.099 9.690
4.040 8.914
2.265 7.779
4.573 8.031
7.299 7.197
9.035 7.391
11.673 7.779
12.556 8.565
12.032 8.768
11.169 8.982
9.481 9.011
7.949 9.030
6.872 8.923
ID= C5367_fodiens

LM= 16
4.434 13.207
2.334 10.888
1.320 11.307
1.915 9.561
2.861 8.859
1.027 7.668
3.583 7.902
6.139 7.200
8.207 7.405
11.232 7.776
12.159 8.498
11.476 8.810
10.471 8.995
8.812 8.898
7.202 8.956
6.002 8.829
ID= C5368_niethammeri

LM= 16
4.016 12.270
1.904 9.799
0.984 10.090
1.633 8.384
2.582 7.793
1.022 6.747
3.396 6.999
5.761 6.475
8.222 6.630
10.693 7.241
11.381 7.997
11.051 8.278
10.073 8.491
8.319 8.626
6.768 8.249
5.635 8.200
ID= C5369_fodiens

LM= 16
4.723 13.827
2.702 11.584
1.895 11.934
2.400 10.408
3.275 9.728
1.691 8.533
3.955 8.707
6.540 7.949
8.348 8.017
10.982 8.426
11.603 9.184
11.166 9.534
10.301 9.524
8.834 9.767
7.376 9.747
6.210 9.553
ID= C5370_fodiens

LM= 16
5.107 13.661
2.878 11.450
1.860 11.945
2.258 10.238
3.111 9.452
1.297 8.453
3.577 8.356
6.894 7.328
8.591 7.464
10.744 7.803
11.899 8.434
11.152 8.676
10.298 8.889
8.698 9.064
7.127 9.151
6.021 9.161
ID= C5371_niethammeri

LM= 16
3.915 13.500
2.061 10.850
1.216 11.189
1.925 9.646
2.867 8.966
1.362 7.684
3.750 8.102
6.439 7.539
8.585 7.723
10.954 8.092
11.692 8.820
11.051 9.053
10.245 9.160
8.692 9.296
7.226 9.218
6.109 9.121
ID= C5372_niethammeri

LM= 16
4.641 11.926
3.091 9.823
1.996 10.104
2.665 8.544
3.440 8.089
1.793 6.945
4.012 7.062
6.521 6.344
8.585 6.470
10.921 6.848
11.609 7.420
11.124 7.643
10.223 7.856
8.818 8.118
7.539 8.040
6.415 7.905
ID= C5374_fodiens

LM= 16
4.479 11.850
2.548 9.474
1.617 9.765
2.345 8.242
3.198 7.476
1.491 6.370
3.916 6.680
6.554 6.098
8.572 6.292
11.200 6.748
12.151 7.612
11.501 7.767
10.580 7.961
8.872 8.048
7.398 7.980
6.176 7.699
ID= C5375_fodiens

LM= 16
4.687 13.554
3.186 11.267
2.120 11.645
2.701 10.017
3.544 9.348
1.926 8.302
4.155 8.408
6.412 7.517
8.767 7.672
11.005 7.953
11.955 8.680
11.296 8.903
10.482 9.164
8.941 9.436
7.449 9.358
6.344 9.290
ID= C5376_fodiens

LM= 16
4.215 13.200
2.165 10.683
1.144 11.013
1.689 9.400
2.719 8.632
0.746 7.485
3.350 7.738
5.955 7.019
8.034 7.155
10.668 7.612
11.620 8.428
11.232 8.690
10.231 8.914
8.491 8.885
6.897 8.817
5.741 8.720
ID= C5377_niethammeri

LM= 16
5.340 12.568
3.459 9.851
2.427 10.231
3.068 8.416
4.090 7.742
2.459 6.611
4.698 6.764
7.437 6.024
9.785 6.296
12.242 7.035
13.242 7.840
12.481 7.927
11.742 8.296
10.014 8.264
8.470 8.079
7.253 7.992
ID= C5603_niethammeri

LM= 16
3.899 13.117
2.193 10.269
0.986 10.628
1.541 8.965
2.562 8.247
0.791 7.258
3.280 7.215
5.965 6.367
8.062 6.421
10.758 6.671
11.758 7.552
11.041 7.736
10.171 7.986
8.410 8.204
6.791 8.247
5.660 8.280
ID= C5604_niethammeri

LM= 16
4.089 13.093
2.271 10.536
1.260 10.851
1.782 9.241
2.685 8.566
0.879 7.348
3.175 7.446
6.178 6.434
8.234 6.553
11.009 6.847
11.923 7.859
11.303 7.979
10.324 8.294
8.550 8.403
6.896 8.479
5.775 8.425
ID= C5605_niethammeri

LM= 16
3.782 13.061
1.705 10.503
0.667 10.778
1.366 9.168
2.394 8.543
0.657 7.452
2.987 7.632
5.307 6.975
8.421 7.251
10.519 7.759
11.144 8.437
10.762 8.702
9.862 8.787
8.156 8.829
6.620 8.818
5.349 8.713
ID= C5697_niethammeri

LM= 16
5.005 12.962
3.401 10.465
2.253 10.688
3.007 9.190
3.836 8.499
2.232 7.447
4.601 7.574
7.099 6.788
9.543 6.979
12.179 7.447
13.156 8.169
12.338 8.371
11.413 8.637
9.787 8.754
8.215 8.701
7.173 8.531
ID= C5699_niethammeri

LM= 16
5.248 13.224
3.081 10.681
2.056 11.072
2.701 9.223
3.610 8.514
1.697 7.563
4.265 7.489
6.971 6.823
8.810 6.971
11.664 7.426
12.489 8.218
11.770 8.388
11.030 8.610
9.445 8.726
7.817 8.652
6.559 8.599
ID= C5701_niethammeri

LM= 16
3.997 13.947
1.987 11.344
0.907 11.852
1.373 10.074
2.368 9.344
0.505 8.222
3.225 8.328
6.336 7.661
7.817 7.894
10.283 8.275
10.875 8.963
10.378 9.270
9.584 9.407
7.987 9.492
6.463 9.439
5.373 9.513
ID= C5709_niethammeri

LM= 16
4.721 13.347
2.743 10.848
1.669 11.358
2.148 9.731
3.147 8.892
1.191 7.988
3.796 7.998
6.582 7.010
8.378 7.073
10.983 7.201
11.866 7.850
11.536 8.211
10.558 8.487
8.836 8.636
7.273 8.594
6.061 8.732
ID= C5741_niethammeri

LM= 16
3.309 11.923
1.489 9.689
0.489 10.040
1.138 8.433
1.968 7.816
0.351 6.668
2.660 6.902
5.202 6.242
7.436 6.572
10.053 7.104
10.691 7.721
10.213 7.976
9.287 8.199
7.638 8.370
6.074 8.168
4.979 7.902
ID= C5742_fodiens

LM= 16
4.798 11.806
3.064 9.465
2.117 9.816
2.649 8.146
3.457 7.593
1.830 6.391
4.234 6.593
6.415 6.061
8.425 6.242
11.308 6.604
12.213 7.423
11.489 7.529
10.670 7.678
9.032 7.763
7.596 7.731
6.404 7.646
ID= C5744_fodiens

LM= 16
4.398 12.838
2.386 10.676
1.534 11.081
2.023 9.473
2.769 8.813
0.980 7.811
3.365 7.865
5.857 7.215
8.094 7.311
10.788 7.598
11.374 8.142
10.820 8.440
9.862 8.695
8.253 8.834
6.762 8.855
5.612 8.727
ID= C6063_fodiens

LM= 16
4.378 12.072
2.763 9.734
1.594 10.106
2.104 8.608
2.880 7.938
1.371 6.738
3.581 7.056
6.387 6.440
8.236 6.695
10.539 7.194
11.336 7.938
10.943 8.140
10.061 8.353
8.658 8.268
7.107 8.119
5.970 7.970
ID= C6064_fodiens

LM= 16
3.765 12.246
2.139 9.957
1.198 10.203
1.957 8.545
2.759 8.011
1.305 6.738
3.540 7.144
6.043 6.642
8.086 6.845
10.695 7.390
11.818 8.203
11.005 8.481
9.904 8.663
8.192 8.513
6.759 8.396
5.658 8.246
ID= C6066_fodiens

LM= 16
4.963 12.742
2.965 10.361
1.881 10.765
2.455 9.224
3.358 8.618
1.690 7.524
3.911 7.651
6.504 7.024
8.353 7.194
10.956 7.545
11.520 8.247
11.041 8.576
10.244 8.682
8.661 8.757
7.205 8.693
6.111 8.597
ID= C6069_fodiens

LM= 16
3.510 11.830
1.560 9.067
0.466 9.479
1.062 7.919
2.080 7.280
0.531 6.002
2.795 6.197
5.503 5.687
7.562 5.904
9.988 6.532
11.017 7.507
10.357 7.605
9.425 7.659
7.908 7.583
6.197 7.464
4.994 7.302
ID= C6103_niethammeri

LM= 16
3.457 12.800
1.440 10.088
0.409 10.490
1.038 8.874
1.982 8.191
0.323 6.835
2.513 7.160
5.084 6.423
7.187 6.575
10.040 7.150
11.027 8.039
10.159 8.212
9.259 8.397
7.686 8.548
6.157 8.408
4.953 8.180
ID= C6104_niethammeri

LM= 2
3.398 13.949
2.634 8.162
ID= C6105_niethammeri

LM= 2
3.905 13.402
3.403 7.438
ID= C6106_niethammeri

LM= 2
5.040 12.552
3.675 7.105
ID= C6108_niethammeri

LM= 16
3.913 12.667
2.198 9.711
1.033 10.067
1.928 8.438
2.964 7.726
1.087 6.453
3.805 6.852
6.416 6.324
9.069 6.820
11.205 7.510
12.036 8.535
11.421 8.589
10.504 8.632
8.821 8.514
7.149 8.287
5.909 8.104
ID= C6109_niethammeri

LM= 16
5.133 12.673
3.042 9.921
2.272 10.138
3.020 8.578
4.201 7.895
2.652 6.465
5.133 7.137
7.635 6.584
9.661 7.050
12.315 7.819
13.041 8.719
12.630 8.979
11.568 8.968
9.791 8.697
8.209 8.426
7.245 8.339
ID= C6110_niethammeri

LM= 16
5.276 14.563
3.521 11.866
2.459 12.180
3.033 10.468
3.434 9.731
2.102 8.735
4.594 8.789
7.345 8.052
9.675 8.431
12.459 9.103
13.228 9.970
12.708 10.219
11.809 10.306
10.184 10.360
8.429 10.067
7.204 9.991
ID= C6111_niethammeri

LM= 16
6.174 12.567
4.434 10.062
3.157 10.267
3.807 8.622
4.683 8.049
2.887 6.988
5.289 7.042
8.179 6.177
10.332 6.350
12.681 6.902
13.503 7.813
12.886 7.943
12.161 8.041
10.386 8.052
8.752 7.943
7.389 8.019
ID= C6112_niethammeri

LM= 16
3.420 12.516
1.844 10.043
0.818 10.410
1.272 8.822
2.135 8.056
0.624 6.976
2.686 7.084
5.569 6.263
7.632 6.393
10.267 6.900
11.001 7.537
10.429 7.861
9.619 8.045
7.783 8.077
6.228 7.991
5.062 7.980
ID= C6113_niethammeri

LM= 2
3.719 14.013
3.344 8.597
ID= C6115_niethammeri

LM= 16
5.146 13.138
3.417 10.357
2.346 10.627
3.049 9.080
3.969 8.311
2.346 7.186
4.705 7.413
7.519 6.796
9.553 7.056
12.194 7.640
12.973 8.420
12.486 8.701
11.415 8.939
9.629 8.885
8.157 8.690
6.891 8.604
ID= C6116_niethammeri

LM= 16
3.759 11.116
2.209 9.330
1.212 9.734
1.767 8.240
2.538 7.639
1.034 6.595
3.167 6.877
5.564 6.266
7.425 6.492
9.897 6.962
10.526 7.573
9.972 7.714
9.229 7.845
7.773 7.921
6.241 7.864
5.075 7.686
ID= C5187_anomalus

LM= 16
4.212 10.572
2.345 8.715
1.238 9.240
1.839 7.570
2.636 6.961
0.891 5.844
3.133 6.041
5.713 5.225
7.552 5.272
10.113 5.591
10.779 6.191
10.366 6.379
9.437 6.632
7.880 6.782
6.463 6.754
5.450 6.698
ID= C5189_anomalus

LM= 16
4.234 11.578
2.410 9.820
1.380 10.293
1.957 8.743
2.779 8.043
1.125 7.079
3.374 7.306
5.992 6.664
7.779 6.730
10.142 7.032
10.794 7.599
10.236 7.713
9.414 7.911
8.015 8.176
6.569 8.232
5.501 8.081
ID= C5190_anomalus

LM= 2
4.713 11.387
3.897 7.199
ID= C5379_anomalus

LM= 16
3.951 11.512
2.243 9.667
1.175 10.114
1.757 8.512
2.534 7.900
0.854 6.833
3.010 7.056
5.495 6.347
7.728 6.328
10.340 6.629
11.223 7.269
10.553 7.454
9.612 7.823
8.155 7.871
6.777 7.930
5.602 7.784
ID= C5381_anomalus

LM= 16
4.193 11.070
2.597 9.202
1.284 9.572
2.023 7.950
2.889 7.425
1.002 6.277
3.512 6.598
5.905 6.072
7.879 6.306
10.545 6.792
11.021 7.386
10.554 7.502
9.737 7.512
8.200 7.707
6.683 7.609
5.671 7.444
ID= C5382_anomalus

LM= 2
4.774 12.723
3.942 8.182
ID= C5383_anomalus

LM= 16
4.159 11.312
2.370 9.598
1.407 9.904
2.243 8.296
2.973 7.778
1.429 6.582
3.587 7.037
6.095 6.497
8.063 6.751
10.635 7.312
11.079 7.852
10.582 7.936
9.714 8.137
8.370 8.296
6.878 8.201
5.820 7.979
ID= C5700_anomalus

LM= 16
4.503 12.019
2.738 10.211
1.861 10.596
2.449 9.067
3.112 8.532
1.476 7.602
3.604 7.751
6.235 7.099
7.604 7.163
10.428 7.430
11.230 7.987
10.321 8.008
9.700 8.329
8.374 8.618
6.979 8.575
5.840 8.438
ID= C5719_anomalus

LM= 16
3.525 11.978
1.597 10.381
0.682 10.892
1.140 9.305
1.938 8.655
0.213 7.633
2.524 7.878
4.899 7.111
6.699 7.069
9.212 7.207
9.851 7.697
9.425 7.793
8.647 8.048
7.220 8.336
5.751 8.485
4.526 8.474
ID= C5720_anomalus

LM= 16
4.403 12.124
2.633 10.450
1.620 10.781
2.345 9.246
3.124 8.574
1.461 7.583
3.827 7.860
6.023 7.220
8.038 7.401
10.768 7.838
11.333 8.318
10.853 8.446
9.968 8.617
8.475 8.766
7.090 8.691
5.991 8.595
ID= C5726_anomalus

LM= 16
4.123 11.342
2.412 9.705
1.265 9.971
2.030 8.472
2.880 7.803
1.148 6.698
3.518 7.091
5.940 6.506
7.726 6.698
10.308 7.208
11.094 7.728
10.372 7.813
9.490 7.983
8.066 8.111
6.684 8.026
5.707 7.920
ID= C6065_anomalus

LM= 16
2.953 11.136
1.183 8.846
0.239 9.171
1.129 7.836
1.911 7.315
0.358 5.871
2.899 6.696
5.092 6.349
7.404 6.772
9.728 7.521
10.401 8.303
9.684 8.194
8.794 8.151
7.437 8.107
5.982 7.803
4.918 7.608
ID= C6119_anomalus

LM= 16
3.156 11.818
1.312 9.988
0.217 10.173
0.868 8.774
1.594 8.275
0.174 7.028
2.451 7.396
5.021 6.800
7.006 7.006
9.273 7.548
9.945 8.123
9.392 8.329
8.470 8.405
7.093 8.481
5.596 8.470
4.436 8.210
ID= C6120_anomalus

LM= 16
3.501 11.350
1.582 9.616
0.497 9.963
1.137 8.411
1.962 7.879
0.138 6.663
2.527 7.086
5.306 6.478
6.793 6.609
9.410 6.945
10.300 7.607
9.507 7.651
8.671 7.803
7.130 7.922
5.740 7.944
4.828 7.857
ID= C6121_anomalus

LM= 16
7.272 11.633
5.476 9.923
4.448 10.399
4.837 8.841
5.670 8.181
3.907 7.142
6.157 7.240
8.646 6.547
10.475 6.677
13.408 6.926
14.014 7.510
13.397 7.618
12.553 7.813
11.103 8.181
9.642 8.181
8.419 8.084
ID= C6124_anomalus

LM=16
3.918 12.102
1.991 9.949
1.076 10.283
1.679 8.743
2.433 8.140
0.926 7.075
3.262 7.193
5.791 6.472
7.739 6.569
10.194 6.946
10.883 7.484
10.409 7.807
9.645 7.968
8.052 8.087
6.502 8.076
5.393 8.044
ID= C101_fodiens

LM=16
3.924 12.464
1.880 10.454
1.043 10.627
1.772 9.117
2.489 8.497
0.826 7.312
3.141 7.606
5.587 6.845
7.652 7.051
10.293 7.345
11.206 8.117
10.554 8.378
9.685 8.704
8.130 8.910
6.685 8.867
5.456 8.584
ID= C1914_fodiens
