# Supplementary material for: Size increase without genetic divergence in the Eurasian water shrew Neomys fodiens
Source: Sci Rep. 2019 Nov 22;9:17375. doi: 10.1038/s41598-019-53891-y (PMC6874603; doi:10.1038/s41598-019-53891-y)
Supplement: Supplementary file 3 — Appendix S2 [file 41598_2019_53891_MOESM3_ESM.pdf]

Appendix S2. Final alignments of the introns used in this study in FASTA format.

Size increase without genetic divergence in the Eurasian water shrew *Neomys fodiens*

Alfonso Balmori-de la Puente [1], Carlos Nores [2], Jacinto Román [3], Angel Fernández-González [4], Pere Aymerich [5], Joaquim Gosálbez [6], Lídia Escoda [1], Jose Castresana [1]

[1] Institute of Evolutionary Biology (CSIC-Universitat Pompeu Fabra), Passeig Marítim de la Barceloneta 37, 08003 Barcelona, Spain

[2] Indurot, Universidad de Oviedo, Campus de Mieres, 33600 Mieres, Asturias, Spain

[3] Department of Conservation Biology, Doñana Biological Station, CSIC, Calle Americo Vespucio 26, 41092 Sevilla, Spain

[4] Biosfera Consultoría Medioambiental S.L., Calle Candamo 5, 33012 Oviedo, Spain

[5] Calle Barcelona 29, 08600 Berga, Barcelona, Spain

[6] Department of Evolutionary Biology, Ecology and Environmental Sciences, University of Barcelona, Avinguda Diagonal 645, 08028 Barcelona, Spain

ASB6-intron-2-oneseq.fasta
>IBE-C1529_anomalus_A
tgcagagcccggcggcagaaggccgtgtgggggaaggtcacccgcctgtcccgagccatgcggctactgggttccgaaggggctgttgccccggggcaccagcttcag
gaaggggcggcagtgctgggtgcgaggaacccccagtcggtgagggagctgagcccagtaggttaggtcacgcccccagttgtggcaggcggcgcgcagggccc
>IBE-C1529_anomalus_B
tgcagagcccggcggcagaaggccgtgtgggggaaggtcacccgcctgtcccgagccatgcggctactgggttccgaaggggctgttgccccggggcaccagcttcag
gaaggggcggcagtgctgggtgcgaggaacccccagtcggtgagggagctgagcccagtaggttaggtcacgcccccagttgtggcaggcggcgcgcagggccc
>IBE-C1789_anomalus_A
tgcagagcccggcggcagaaggccgtgtgggggaaggtcacccgcctgtcccgagccatgcggctactgggttccgaaggggctgttgccccggggcaccagcttcag
gaaggggcggcagtgctgggtgcgaggaacccccagtcggtgagggagctgagcccagtaggttaggtcacgcccccagttgtggcaggcggcgcgcagggccc
>IBE-C1789_anomalus_B
tgcagagcccggcggcagaaggccgtgtgggggaaggtcacccgcctgtcccgagccatgcggctactgggttccgaaggggctgttgccccggggcaccagcttcag
gaaggggcggcagtgctgggtgcgaggaacccccagtcggtgagggagctgagcccagtaggttaggtcacgcccccagttgtggcaggcggcgcgcagggccc
>IBE-C1808_milleri_A
tgcagagcccggcggcagaaggccgtgtgggggaaggtcacccgcctgtcccgagccatgcggctactgggttccgaaggggctgttgccccggggcaccagcttcag
gaaggggtggcagtgctgggtgcgaggaacccccagtcggtgagggagctgagcccagtaggttaggtcacgcccccagttgtggcaggcggcgcgcagggccc
>IBE-C1808_milleri_B
tgcagagcccggcggcagaaggccgtgtgggggaaggtcacccgcctgtcccgagccatgcggctactgggttccgaaggggctgttgccccggggcaccagcttcag
gaaggggtggcagtgctgggtgcgaggaacccccagtcggtgagggagctgagcccagtaggttaggtcacgcccccagttgtggcaggcggcgcgcagggccc
>IBE-C2895_anomalus_A
tgcagagcccggcggcagaaggccgtgtgggggaaggtcacccgcctgtcccgagccatgcggctactgggttccgaaggggctgttgccccggggcaccagcttcag
gaaggggcggcagtgctgggtgcgaggaacccccagtcggtgagggagctgagcccagtaggttaggtcacgcccccagttgtggcaggcggcgcgcagggccc
>IBE-C2895_anomalus_B
tgcagagcccggcggcagaaggccgtgtgggggaaggtcacccgcctgtcccgagccatgcggctactgggttccgaaggggctgttgccccggggcaccagcttcag
gaaggggcggcagtgctgggtgcgaggaacccccagtcggtgagggagctgagcccagtaggttaggtcacgcccccagttgtggcaggcggcgcgcagggccc
>IBE-C3786_milleri_A
tgcagagcccggcggcagaaggccgtgtgggggaaggtcacccgcctgtcccgagccatgcggctactgggttccgaaggggctgttgccccggggcaccagcttcag
gaaggggtggcagtgctgggtgcgaggaacccccagtcggtgagggagctgagcccagtaggttaggtcacgcccccagttgtggcaggcggcgcgcagggccc
>IBE-C3786_milleri_B
tgcagagcccggcggcagaaggccgtgtgggggaaggtcacccgcctgtcccgagccatgcggctactgggttccgaaggggctgttgccccggggcaccagcttcag
gaaggggtggcagtgctgggtgcgaggaacccccagtcggtgagggagctgagcccagtaggttaggtcacgcccccagttgtggcaggcggcgcgcagggccc
>IBE-C4116_milleri_A
tgcagagcccggcggcagaaggccgtgtgggggaaggtcacccgcctgtcccgagccatgcggctactgggttccgaaggggctgttgccccggggcaccagcttcag
gaaggggtggcagtgctgggtgcgaggaacccccagtcggtgagggagctgagcccagtaggttaggtcacgcccccagttgtggcaggcggcgcgcagggccc
>IBE-C4116_milleri_B
tgcagagcccggcggcagaaggccgtgtgggggaaggtcacccgcctgtcccgagccatgcggctactgggttccgaaggggctgttgccccggggcaccagcttcag
gaaggggtggcagtgctgggtgcgaggaacccccagtcggtgagggagctgagcccagtaggttaggtcacgcccccagttgtggcaggcggcgcgcagggccc
>IBE-C101_fodiens_A
tgcagagccaggcggcagaaggccgtgtgggggaaggtcacccgcctgtcccgagccatgcggctactgggttccgaaggggctgttgccccggggcaccagcttcag
gaaggggcggcagtgctgggtgcgaggaaaccccagtcggtgagggagctgagcccagtaggttaggtcacgcccccagttgtggcaggcggcgagcagggccc
>IBE-C101_fodiens_B
tgcagagccaggcggcagaaggccgtgtgggggaaggtcacccgcctgtcccgagccatgcggctactgggttccgaaggggctgttgccccggggcaccagcttcag
gaaggggcggcagtgctgggtgcgaggaaaccccagtcggtgagggagctgagcccagtaggttaggtcacgcccccagttgtggcaggcggcgagcagggccc
>IBE-C1914_fodiens_A
tgcagagccaggcggcagaaggccgtgtgggggaaggtcacccgcctgtcccgagccatgcggctactgggttccgaaggggctgttgccccggggcaccagcttcag
gaaggggcggcagtgctgggtgcgaggaaaccccagtcggtgagggagctgagcccagtaggttaggtcacgcccccagttgtggcaggcggcgagcagggccc
>IBE-C1914_fodiens_B
tgcagagccaggcggcagaaggccgtgtgggggaaggtcacccgcctgtcccgagccatgcggctactgggttccgaaggggctgttgccccggggcaccagcttcag
gaaggggcggcagtgctgggtgcgaggaaaccccagtcggtgagggagctgagcccagtaggttaggtcacgcccccagttgtggcaggcggcgagcagggccc
>IBE-C4120_teres_A
tgcagagcccggcggcagaaggccgtgtgggggaaggtcacccgcctgtcccgagccatgcggctactgggttccgaaggggctgttgccccggggcaccagcttcag
gaaggggcggcagtgctgggtgcgaggaacccccagtcagtgagggagctgagcccagtaggttaggtcacgcccccagttgtggcaggcggcgcgcagggccc
>IBE-C4120_teres_B
tgcagagcccggcggcagaaggccgtgtgggggaaggtcacccgcctgtcccgagccatgcggctactgggttccgaaggggctgttgccccggggcaccagcttcag
gaaggggcggcagtgctgggtgcgaggaacccccagtcggtgagggagctgagcccagtaggttaggtcacgcccccagttgtggcaggcggcgcgcagggccc
>IBE-C4122_teres_A
tgcagagccgggcggcagaaggccgtgtgggggaaggtcacccgcctgtcccgagccatgcggctactgggttccgaaggggctgttgccccggggcaccagcttcag
gaaggggcggcagtgctgggtgcgaggaacccccagtcggtgagggagctgagcccagtaggttaggtcacgcccccagttgtggcaggcggcgcgcagggccc
>IBE-C4122_teres_B
tgcagagcccggcggcagaaggccgtgtgggggaaggtcacccgcctgtcccgagccatgcggctactgggttccgaaggggctgttgccccggggcaccagcttcag
gaaggggcggcagtgctgggtgcgaggaacccccagtcggtgagggagctgagcccagtaggttaggtcacgcccccagttgtggcaggcggcgcgcagggccc
>IBE-C1927_milleri_A
tgcagagcccggcggcagaaggccgtgtgggggaaggtcacccgcctgtcccgagccatgcggctactgggttccgaaggggctgttgccccggggcaccagcttcag
gaaggggtggcagtgctgggtgcgaggaacccccagtcggtgagggagctgagcccagtaggttaggtcacgcccccagttgtggcaggcggcgcgcagggccc
>IBE-C1927_milleri_B
tgcagagcccggcggcagaaggccgtgtgggggaaggtcacccgcctgtcccgagccatgcggctactgggttccgaaggggctgttgccccggggcaccagcttcag
gaaggggtggcagtgctgggtgcgaggaacccccagtcggtgagggagctgagcccagtaggttaggtcacgcccccagttgtggcaggcggcgcgcagggccc
>IBE-C5182_niethammeri_A
tgcagagccaggcggcagaaggccgtgtgggggaaggtcacccgcctgtcccgagccatgcggctactgggttccgaaggggctgttgccccggggcaccagcttcag
gaaggggcggcagtgctgggtgcgaggaaaccccagtcggtgagggagctgagcccagtaggttaggtcacgcccccagttgtggcaggcggcgagcagggccc
>IBE-C5182_niethammeri_B
tgcagagccaggcggcagaaggccgtgtgggggaaggtcacccgcctgtcccgagccatgcggctactgggttccgaaggggctgttgccccggggcaccagcttcag
gaaggggcggcagtgctgggtgcgaggaaaccccagtcggtgagggagctgagcccagtaggttaggtcacgcccccagttgtggcaggcggcgagcagggccc
>IBE-C5367_fodiens_A

tgcagagccaggcggcagaaggccgtgtgggggaaggtcacccgcctgtcccgagccatgcggctactgggttccgaaggggctgttgccccggggcaccagcttcag
gaaggggcggcagtgctgggtgcgaggaaaccccagtcggtgaggggagctgagcccagtaggttaggtcacgcccccagttgtggcaggcggcgagcagggccc
>IBE-C5367_fodiens_B
tgcagagccaggcggcagaaggccgtgtgggggaaggtcacccgcctgtcccgagccatgcggctactgggttccgaaggggctgttgccccggggcaccagcttcag
gaaggggcggcagtgctgggtgcgaggaaaccccagtcggtgaggggagctgagcccagtaggttaggtcacgcccccagttgtggcaggcggcgagcagggccc
>IBE-C5368_niethammeri_A
tgcagagccaggcggcagaaggccgtgtgggggaaggtcacccgcctgtcccgagccatgcggctactgggttccgaaggggctgttgccccggggcaccagcttcag
gaaggggcggcagtgctgggtgcgaggaaaccccagtcggtgaggggagctgagcccagtaggttaggtcacgcccccagttgtggcaggcggcgagcagggccc
>IBE-C5368_niethammeri_B
tgcagagccaggcggcagaaggccgtgtgggggaaggtcacccgcctgtcccgagccatgcggctactgggttccgaaggggctgttgccccggggcaccagcttcag
gaaggggcggcagtgctgggtgcgaggaaaccccagtcggtgaggggagctgagcccagtaggttaggtcacgcccccagttgtggcaggcggcgagcagggccc
>IBE-C5697_niethammeri_A
tgcagagccaggcggcagaaggccgtgtgggggaaggtcacccgcctgtcccgagccatgcggctactgggttccgaaggggctgttgccccggggcaccagcttcag
gaaggggcggcagtgctgggtgcgaggaaaccccagtcggtgaggggagctgagcccagtaggttaggtcacgcccccagttgtggcaggcggcgagcagggccc
>IBE-C5697_niethammeri_B
tgcagagccaggcggcagaaggccgtgtgggggaaggtcacccgcctgtcccgagccatgcggctactgggttccgaaggggctgttgccccggggcaccagcttcag
gaaggggcggcagtgctgggtgcgaggaaaccccagtcggtgaggggagctgagcccagtaggttaggtcacgcccccagttgtggcaggcggcgagcagggccc
>IBE-C5700_anomalus_A
tgcagagcccggcggcagaaggccgtgtgggggaaggtcacccgcctgtcccgagccatgcggctactgggttccgaaggggctgttgccccggggcaccagcttcag
gaaggggcggcagtgctgggtgcgaggaacccccagtcggtgaggggagctgagcccagtaggttaggtcacgcccccagttgtggcaggcggcgcgcagggccc
>IBE-C5700_anomalus_B
tgcagagcccggcggcagaaggccgtgtgggggaaggtcacccgcctgtcccgagccatgcggctactgggttccgaaggggctgttgccccggggcaccagcttcag
gaaggggcggcagtgctgggtgcgaggaacccccagtcggtgaggggagctgagcccagtaggttaggtcacgcccccagttgtggcaggcggcgcgcagggccc
>IBE-C5701_niethammeri_A
tgcagagccaggcggcagaaggccgtgtgggggaaggtcacccgcctgtcccgagccatgcggctactgggttccgaaggggctgttgccccggggcaccagcttcag
gaaggggcggcagtgctgggtgcgaggaaaccccagtcggtgaggggagctgagcccagtaggttaggtcacgcccccagttgtggcaggcggcgagcagggccc
>IBE-C5701_niethammeri_B
tgcagagccaggcggcagaaggccgtgtgggggaaggtcacccgcctctcccgagccatgcggctactgggttccgaaggggctgttgccccggggcaccagcttcag
gaaggggcggcagtgctgggtgcgaggaaaccccagtcggtgaggggagctgagcccagtaggttaggtcacgcccccagttgtggcaggcggcgagcagggccc
>IBE-C5370_fodiens_A
tgcagagccaggcggcagaaggccgtgtgggggaaggtcacccgcctgtcccgagccatgcggctactgggttccgaaggggctgttgccccggggcaccagcttcag
gaaggggcggcagtgctgggtgcgaggaaaccccagtcggtgaggggagctgagcccagtaggttaggtcacgcccccagttgtggcaggcggcgagcagggccc
>IBE-C5370_fodiens_B
tgcagagccaggcggcagaaggccgtgtgggggaaggtcacccgcctgtcccgagccatgcggctactgggttccgaaggggctgttgccccggggcaccagcttcag
gaaggggcggcagtgctgggtgcgaggaaaccccagtcggtgaggggagctgagcccagtaggttaggtcacgcccccagttgtggcaggcggcgagcagggccc
>IBE-C5383_anomalus_A
tgcagagcccggcggcagaaggccgtgtgggggaaggtcacccgcctgtcccgagccatgcggctactgggttccgaaggggctgttgccccggggcaccagcttcag
gaaggggcggcagtgctgggtgcgaggaacccccagtcggtgaggggagctgagcccagtaggttaggtcacgcccccagttgtggcaggcggcgcgcagggccc
>IBE-C5383_anomalus_B
tgcagagcccggcggcagaaggccgtgtgggggaaggtcacccgcctgtcccgagccatgcggctactgggttccgaaggggctgttgccccggggcaccagcttcag
gaaggggcggcagtgctgggtgcgaggaacccccagtcggtgaggggagctgagcccagtaggttaggtcacgcccccagttgtggcaggcggcgcgcagggccc
>IBE-C5603_niethammeri_A
tgcagagccaggcggcagaaggccgtgtgggggaaggtcacccgcctgtcccgagccatgcggctactgggttccgaaggggctgttgccccggggcaccagcttcag
gaaggggcggcagtgctgggtgcgaggaaaccccagtcggtgaggggagctgagcccagtaggttaggtcacgcccccagttgtggcaggcggcgagcagggccc
>IBE-C5603_niethammeri_B
tgcagagccaggcggcagaaggccgtgtgggggaaggtcacccgcctgtcccgagccatgcggctactgggttccgaaggggctgttgccccggggcaccagcttcag
gaaggggcggcagtgctgggtgcgaggaaaccccagtcggtgaggggagctgagcccagtaggttaggtcacgcccccagttgtggcaggcggcgagcagggccc
>IBE-C5604_niethammeri_A
tgcagagccaggcggcagaaggccgtgtgggggaaggtcacccgcctgtcccgagccatgcggctactgggttccgaaggggctgttgccccggggcaccagcttcag
gaaggggcggcagtgctgggtgcgaggaaaccccagtcggtgaggggagctgagcccagtaggttaggtcacgcccccagttgtggcaggcggcgagcagggccc
>IBE-C5604_niethammeri_B
tgcagagccaggcggcagaaggccgtgtgggggaaggtcacccgcctgtcccgagccatgcggctactgggttccgaaggggctgttgccccggggcaccagcttcag
gaaggggcggcagtgctgggtgcgaggaaaccccagtcggtgaggggagctgagcccagtaggttaggtcacgcccccagttgtggcaggcggcgagcagggccc
>IBE-C5719_anomalus_A
tgcagagcccggcggcagaaggccgtgtgggggaaggtcacccgcctgtcccgagccatgcggctactgggttccgaaggggctgttgccccggggcaccagcttcag
gaaggggcggcagtgctgggtgcgaggaacccccagtcggtgaggggagctgagcccagtaggttaggtcacgcccccagttgtggcaggcggcgcgcagggccc
>IBE-C5719_anomalus_B
tgcagagcctggcggcagaaggccgtgtgggggaaggtcacccgcctgtcccgagccatgcggctactgggttccgaaggggctgttgccccggggcaccagcttcag
gaaggggcggcagtgctgggtgcgaggaacccccagtcggtgaggggagctgagcccagtaggttaggtcacgcccccagttgtggcaggcggcgcgcagggccc
>IBE-C5720_anomalus_A
tgcagagcccggcggcagaaggccgtgtgggggaaggtcacccgcctgtcccgagccatgcggctactgggttccgaaggggctgttgccccggggcaccagcttcag
gaaggggcggcagtgctgggtgcgaggaacccccagtcggtgaggggagctgagcccagtaggttaggtcacgcccccagttgtggcaggcggcgcgcagggccc
>IBE-C5720_anomalus_B
tgcagagcccggcggcagaaggccgtgtgggggaaggtcacccgcctgtcccgagccatgcggctactgggttccgaaggggctgttgccccggggcaccagcttcag
gaaggggcggcagtgctgggtgcgaggaacccccagtcggtgaggggagctgagcccagtaggttaggtcacgcccccagttgtggcaggcggcgcgcagggccc
>IBE-C5699_niethammeri_A
tgcagagccaggcggcagaaggccgtgtgggggaaggtcacccgcctgtcccgagccatgcggctactgggttccgaaggggctgttgccccggggcaccagcttcag
gaaggggcggcagtgctgggtgcgaggaaaccccagtcggtgaggggagctgagcccagtaggttaggtcacgcccccagttgtggcaggcggcgagcagggccc
>IBE-C5699_niethammeri_B
tgcagagccaggcggcagaaggccgtgtgggggaaggtcacccgcctgtcccgagccatgcggctactgggttccgaaggggctgttgccccggggcaccagcttcag
gaaggggcggcagtgctgggtgcgaggaaaccccagtcggtgaggggagctgagcccagtaggttaggtcacgcccccagttgtggcaggcggcgagcagggccc
>IBE-C5741_niethammeri_A
tgcagagccaggcggcagaaggccgtgtgggggaaggtcacccgcctgtcccgagccatgcggctactgggttccgaaggggctgttgccccggggcaccagcttcag
gaaggggcggcagtgctgggtgcgaggaaaccccagtcggtgaggggagctgagcccagtaggttaggtcacgcccccagttgtggcaggcagcgagcagggccc

>IBE-C5741_niethammeri_B
tgcagagccaggcggcagaaggccgtgtgggggaaggtcacccgcctgtcccgagccatgcggctactgggttccgaaggggctgttgccccggggcaccagcttcag
gaaggggcggcagtgctgggtgcgaggaaaccccagtcggtgagggagctgagcccagtaggttaggtcacgcccccagttgtggcaggcggcgagcagggccc
>UAM-24769_fodiens_A
tgcagagccaggcggcagaaggccgtgtgggggaaggtcacccgcctgtcccgagccatgcggctactgggttccgaaggggctgttgccccggggcaccagcttcag
gaaggggcggcagtgctgggtgcgaggaaaccccagtcggtgagggagctgagcccagtaggttaggtcacgcccccagttgtggcaggcggcgagcagggccc
>UAM-24769_fodiens_B
tgcagagccaggcggcagaaggccgtgtgggggaaggtcacccgcctgtcccgagccatgcggctactgggttccgaaggggctgttgccccggggcaccagcttcag
gaaggggcggcagtgctgggtgcgaggaaaccccagtcggtgagggagctgagcccagtaggttaggtcacgcccccagttgtggcaggcggcgagcagggccc
>UAM-24770_fodiens_A
tgcagagccaggcggcagaaggccgtgtgggggaaggtcacccgcctgtcccgagccatgcggctactgggttccgaaggggctgttgccccggggcaccagcttcag
gaaggggcagcagtgctgggtgcgaggaaaccccagtcggtgagggagctgagcccagtaggttaggtcacgcccccagttgtggcaggcggcgagcagggccc
>UAM-24770_fodiens_B
tgcagagccaggcggcagaaggccgtgtgggggaaggtcacccgcctgtcccgagccatgcggctactgggttccgaaggggctgttgccccggggcaccagcttcag
gaaggggcggcagtgctgggtgcgaggaaaccccagtcggtgagggagctgagcccagtaggttaggtcacgcccccagttgtggcaggcggcgagcagggccc
>IBE-C5372_niethammeri_A
tgcagagccaggcggcagaaggccgtgtgggggaaggtcacccgcctgtcccgagccatgcggctactgggttccgaaggggctgttgccccggggcaccagcttcag
gaaggggcggcagtgctgggtgcgaggaaaccccagtcggtgagggagctgagcccagtaggttaggtcacgcccccagttgtggcaggcggcgagcagggccc
>IBE-C5372_niethammeri_B
tgcagagccaggcggcagaaggccgtgtgggggaaggtcacccgcctctcccgagccatgcggctactgggttccgaaggggctgttgccccggggcaccagcttcag
gaaggggcggcagtgctgggtgcgaggaaaccccagtcggtgagggagctgagcccagtaggttaggtcacgcccccagttgtggcaggcggcgagcagggccc
>IBE-C5374_fodiens_A
tgcagagccaggcggcagaaggccgtgtgggggaaggtcacccgcctgtcccgagccatgcggctactgggttccgaaggggctgttgccccggggcaccagcttcag
gaaggggcggcagtgctgggtgcgaggaaaccccagtcggtgagggagctgagcccagtaggttaggtcacgcccccagttgtggcaggcggcgagcagggccc
>IBE-C5374_fodiens_B
tgcagagccaggcggcagaaggccgtgtgggggaaggtcacccgcctgtcccgagccatgcggctactgggttccgaaggggctgttgccccggggcaccagcttcag
gaaggggcggcagtgctgggtgcgaggaaaccccagtcggtgagggagctgagcccagtaggttaggtcacgcccccagttgtggcaggcggcgagcagggccc
>IBE-C5376_fodiens_A
tgcagagccaggcggcagaaggccgtgtgggggaaggtcacccgcctgtcccgagccatgcggctactgggttccgaaggggctgttgccccggggcaccagcttcag
gaaggggcggcagtgctgggtgcgaggaaaccccagtcggtgagggagctgagcccagtaggttaggtcacgcccccagttgtggcaggcggcgagcagggccc
>IBE-C5376_fodiens_B
tgcagagccaggcggcagaaggccgtgtgggggaaggtcacccgcctgtcccgagccatgcggctactgggttccgaaggggctgttgccccggggcaccagcttcag
gaaggggcggcagtgctgggtgcgaggaaaccccagtcggtgagggagctgagcccagtaggttaggtcacgcccccagttgtggcaggcggcgagcagggccc
>IBE-C5726_anomalus_A
tgcagagcccggcggcagaaggccgtgtgggggaaggtcacccgcctgtcccgagccatgcggctactgggttccgaaggggctgttgccccggggcaccagcttcag
gaaggggcggcagtgctgggtgcgaggaacccccagtcggtgagggagctgagcccagtaggttaggtcacgcccccagttgtggcaggcggcgcgcagggccc
>IBE-C5726_anomalus_B
tgcagagcctggcggcagaaggccgtgtgggggaaggtcacccgcctgtcccgagccatgcggctactgggttccgaaggggctgttgccccggggcaccagcttcag
gaaggggcggcagtgctgggtgcgaggaacccccagtcggtgagggagctgagcccagtaggttaggtcacgcccccagttgtggcaggcggcgcgcagggccc
>IBE-C5742_fodiens_A
tgcagagccaggcggcagaaggccgtgtgggggaaggtcacccgcctgtcccgagccatgcggctactgggttccgaaggggctgttgccccggggcaccagcttcag
gaaggggcggcagtgctgggtgcgaggaaaccccagtcggtgagggagctgagcccagtaggttaggtcacgcccccagttgtggcaggcggcgagcagggccc
>IBE-C5742_fodiens_B
tgcagagccaggcggcagaaggccgtgtgggggaaggtcacccgcctgtcccgagccatgcggctactgggttccgaaggggctgttgccccggggcaccagcttcag
gaaggggcggcagtgctgggtgcgaggaaaccccagtcggtgagggagctgagcccagtaggttaggtcacgcccccagttgtggcaggcggcgagcagggccc
>IBE-C5744_fodiens_A
tgcagagccaggcggcagaaggccgtgtgggggaaggtcacccgcctgtcccgagccatgcggctactgggttccgaaggggctgttgccccggggcaccagcttcag
gaaggggcggcagtgctgggtgcgaggaaaccccagtcggtgagggagctgagcccagtaggttaggtcacgcccccagttgtggcaggcggcgagcagggccc
>IBE-C5744_fodiens_B
tgcagagccaggcggcagaaggccgtgtgggggaaggtcacccgcctctcccgagccatgcggctactgggttccgaaggggctgttgccccggggcaccagcttcag
gaaggggcggcagtgctgggtgcgaggaaaccccagtcggtgagggagctgagcccagtaggttaggtcacgcccccagttgtggcaggcggcgagcagggccc
>IBE-C6063_fodiens_A
tgcagagccaggcggcagaaggccgtgtgggggaaggtcacccgcctgtcccgagccatgcggctactgggttccgaaggggctgttgccccggggcaccagcttcag
gaaggggcggcagtgctgggtgcgaggaaaccccagtcggtgagggagctgagcccagtaggttaggtcacgcccccagttgtggcaggcggcgagcagggccc
>IBE-C6063_fodiens_B
tgcagagccaggcggcagaaggccgtgtgggggaaggtcacccgcctgtcccgagccatgcggctactgggttccgaaggggctgttgccccggggcaccagcttcag
gaaggggcggcagtgctgggtgcgaggaaaccccagtcggtgagggagctgagcccagtaggttaggtcacgcccccagttgtggcaggcggcgagcagggccc
>IBE-C6064_fodiens_A
tgcagagccaggcggcagaaggccgtgtgggggaaggtcacccgcctgtcccgagccatgcggctactgggttccgaaggggctgttgccccggggcaccagcttcag
gaaggggcggcagtgctgggtgcgaggaaaccccagtcggtgagggagctgagcccagtaggttaggtcacgcccccagttgtggcaggcggcgagcagggccc
>IBE-C6064_fodiens_B
tgcagagccaggcggcagaaggccgtgtgggggaaggtcacccgcctgtcccgagccatgcggctactgggttccgaaggggctgttgccccggggcaccagcttcag
gaaggggcggcagtgctgggtgcgaggaaaccccagtcggtgagggagctgagcccagtaggttaggtcacgcccccagttgtggcaggcggcgagcagggccc
>IBE-C6065_anomalus_A
tgcagagcccggcggcagaaggccgtgtgggggaaggtcacccgcctgtcccgagccatgcggctactgggttccgaaggggctgttgccccggggcaccagcttcag
gaaggggcggcagtgctgggtgcgaggaacccccagtcggtgagggagctgagcccagtaggttaggtcacgcccccagttgtggcaggcggcgcgcagggccc
>IBE-C6065_anomalus_B
tgcagagcctggcggcagaaggccgtgtgggggaaggtcacccgcctgtcccgagccatgcggctactgggttccgaaggggctgttgccccggggcaccagcttcag
gaaggggcggcagtgctgggtgcgaggaacccccagtcggtgagggagctgagcccagtaggttaggtcacgcccccagttgtggcaggcggcgcgcagggccc
>IBE-C6066_fodiens_A
tgcagagccaggcggcagaaggccgtgtgggggaaggtcacccgcctgtcccgagccatgcggctactgggttccgaaggggctgttgccccggggcaccagcttcag
gaaggggcggcagtgctgggtgcgaggaaaccccagtcggtgagggagctgagcccagtaggttaggtcacgcccccagttgtggcaggcggcgagcagggccc
>IBE-C6066_fodiens_B

tgcagagccaggcggcagaaggccgtgtgggggaaggtcacccgcctgtcccgagccatgcggctactgggttccgaaggggctgttgccccggggcaccagcttcag
gaaggggcggcagtgctgggtgcgaggaaaccccagtcggtgagggagctgagcccagtaggttaggtcacgcccccagttgtggcaggcggcgagcagggccc
>IBE-C6069_fodiens_A
tgcagagccaggcggcagaaggccgtgtgggggaaggtcacccgcctgtcccgagccatgcggctactgggttccgaaggggctgttgccccggggcaccagcttcag
gaaggggcggcagtgctgggtgcgaggaaaccccagtcggtgagggagctgagcccagtaggttaggtcacgcccccagttgtggcaggcggcgagcagggccc
>IBE-C6069_fodiens_B
tgcagagccaggcggcagaaggccgtgtgggggaaggtcacccgcctgtcccgagccatgcggctactgggttccgaaggggctgttgccccggggcaccagcttcag
gaaggggcggcagtgctgggtgcgaggaaaccccagtcggtgagggagctgagcccagtaggttaggtcacgcccccagttgtggcaggcggcgagcagggccc
>IBE-C4117_fodiens_A
tgcagagccaggcggcagaaggccgtgtgggggaaggtcacccgcctgtcccgagccatgcggctactgggttccgaaggggctgttgccccggggcaccagcttcag
gaaggggcggcagtgctgggtgcgaggaaaccccagtcggtgagggagctgagcccagtaggttaggtcacgcccccagttgtggcaggcggcgagcagggccc
>IBE-C4117_fodiens_B
tgcagagccaggcggcagaaggccgtgtgggggaaggtcacccgcctgtcccgagccatgcggctactgggttccgaaggggctgttgccccggggcaccagcttcag
gaaggggcggcagtgctgggtgcgaggaaaccccagtcggtgagggagctgagcccagtaggttaggtcacgcccccagttgtggcaggcggcgagcagggccc
>IBE-C6105_niethammeri_A
tgcagagccaggcggcagaaggccgtgtgggggaaggtcacccgcctgtcccgagccatgcggctactgggttccgaaggggctgttgccccggggcaccagcttcag
gaaggggcggcagtgctgggtgcgaggaaaccccagtcggtgagggagctgagcccagtaggttaggtcacgcccccagttgtggcaggcggcgagcagggccc
>IBE-C6105_niethammeri_B
tgcagagccaggcggcagaaggccgtgtgggggaaggtcacccgcctgtcccgagccatgcggctactgggttccgaaggggctgttgccccggggcaccagcttcag
gaaggggcggcagtgctgggtgcgaggaaaccccagtcggtgagggagctgagcccagtaggttaggtcacgcccccagttgtggcaggcggcgagcagggccc
>IBE-C6106_niethammeri_A
tgcagagccaggcggcagaaggccgtgtgggggaaggtcacccgcctgtcccgagccatgcggctactgggttccgaaggggctgttgccccggggcaccagcttcag
gaaggggcggcagtgctgggtgcgaggaaaccccagtcggtgagggagctgagcccagtaggttaggtcacgcccccagttgtggcaggcggcgagcagggccc
>IBE-C6106_niethammeri_B
tgcagagccaggcggcagaaggccgtgtgggggaaggtcacccgcctgtcccgagccatgcggctactgggttccgaaggggctgttgccccggggcaccagcttcag
gaaggggcggcagtgctgggtgcgaggaaaccccagtcggtgagggagctgagcccagtaggttaggtcacgcccccagttgtggcaggcggcgagcagggccc
>IBE-C6110_niethammeri_A
tgcagagccaggcggcagaaggccgtgtgggggaaggtcacccgcctgtcccgagccatgcggctactgggttccgaaggggctgttgccccggggcaccagcttcag
gaaggggcggcagtgctgggtgcgaggaaaccccagtcggtgagggagctgagcccagtaggttaggtcacgcccccagttgtggcaggcggcgagcagggccc
>IBE-C6110_niethammeri_B
tgcagagccaggcggcagaaggccgtgtgggggaaggtcacccgcctgtcccgagccatgcggctactgggttccgaaggggctgttgccccggggcaccagcttcag
gaaggggcggcagtgctgggtgcgaggaaaccccagtcggtgagggagctgagcccagtaggttaggtcacgcccccagttgtggcaggcgacgagcagggccc
>IBE-C6124_anomalus_A
tgcagagcccggcggcagaaggccgtgtgggggaaggtcacccgcctgtcccgagccatgcggctactgggttccgaaggggctgttgccccggggcaccagcttcag
gaaggggcggcagtgctgggtgcgaggaacccccagtcggtgagggagctgagcccagtaggttaggtcacgcccccagttgtggcaggcggcgcgcagggccc
>IBE-C6124_anomalus_B
tgcagagcccggcggcagaaggccgtgtgggggaaggtcacccgcctgtcccgagccatgcggctactgggttccgaaggggctgttgccccggggcaccagcttcag
gaaggggcggcagtgctgggtgcgaggaacccccagtcggtgagggagctgagcccagtaggttaggtcacgcccccagttgtggcaggcggcgcgcagggccc
>IBE-C6125_anomalus_A
tgcagagcccggcggcagaaggccgtgtgggggaaggtcacccgcctgtcccgagccatgcggctactgggttccgaaggggctgttgccccggggcaccagcttcag
gaaggggcggcagtgctgggtgcgaggaacccccagtcggtgagggagctgagcccagtaggttaggtcacgcccccagttgtggcaggcggcgcgcagggccc
>IBE-C6125_anomalus_B
tgcagagcctggcggcagaaggccgtgtgggggaaggtcacccgcctgtcccgagccatgcggctactgggttccgaaggggctgttgccccggggcaccagcttcag
gaaggggcggcagtgctgggtgcgaggaacccccagtcggtgagggagctgagcccagtaggttaggtcacgcccccagttgtggcaggcggcgcgcagggccc
>IBE-C5188_fodiens_A
tgcagagccaggcggcagaaggccgtgtgggggaaggtcacccgcctgtcccgagccatgcggctactgggttccgaaggggctgttgccccggggcaccagcttcag
gaaggggcggcagtgctgggtgcgaggaaaccccagtcggtgagggagctgagcccagtaggttaggtcacgcccccagttgtggcaggcggcgagcagggccc
>IBE-C5188_fodiens_B
tgcagagccaggcggcagaaggccgtgtgggggaaggtcacccgcctctcccgagccatgcggctactgggttccgaaggggctgttgccccggggcaccagcttcag
gaaggggcggcagtgctgggtgcgaggaaaccccagtcggtgagggagctgagcccagtaggttaggtcacgcccccagttgtggcaggcggcgagcagggccc
>IBE-C5359_niethammeri_A
tgcagagccaggcggcagaaggccgtgtgggggaaggtcacccgcctgtcccgagccatgcggctactgggttccgaaggggctgttgccccggggcaccagcttcag
gaaggggcggcagtgctgggtgcgaggaaaccccagtcggtgagggagctgagcccagtaggttaggtcacgcccccagttgtggcaggcggcgagcagggccc
>IBE-C5359_niethammeri_B
tgcagagccaggcggcagaaggccgtgtgggggaaggtcacccgcctgtcccgagccatgcggctactgggttccgaaggggctgttgccccggggcaccagcttcag
gaaggggcggcagtgctgggtgcgaggaaaccccagtcggtgagggagctgagcccagtaggttaggtcacgcccccagttgtggcaggcggcgagcagggccc
>IBE-C5371_niethammeri_A
tgcagagccaggcggcagaaggccgtgtgggggaaggtcacccgcctgtcccgagccatgcggctactgggttccgaaggggctgttgccccggggcaccagcttcag
gaaggggcggcagtgctgggtgcgaggaaaccccagtcggtgagggagctgagcccagtaggttaggtcacgcccccagttgtggcaggcggcgagcagggccc
>IBE-C5371_niethammeri_B
tgcagagccaggcggcagaaggccgtgtgggggaaggtcacccgcctgtcccgagccatgcggctactgggttccgaaggggctgttgccccggggcaccagcttcag
gaaggggcggcagtgctgggtgcgaggaaaccccagtcggtgagggagctgagcccagtaggttaggtcacgcccccagttgtggcaggcggcgagcagggccc
>MSB-158495_fodiens_A
tgcagagccaggcggcagaaggccgtgtgggggaaggtcacccgcctgtcccgagccatgcggctactgggttccgaaggggctgttgccccggggcaccagcttcag
gaaggggcggcagtgctgggtgcgaggaaaccccagtcggtgagggagctgagcccagtaggttaggtcacgcccccagttgtggcaggcggcgagcagggccc
>MSB-158495_fodiens_B
tgcagagccaggcggcagaaggccgtgtgggggaaggtcacccgcctgtcccgagccatgcggctactgggttccgaaggggctgttgccccggggcaccagcttcag
gaaggggcggcagtgctgggtgcgaggaaaccccagtcggtgagggagctgagcccagtaggttaggtcacgcccccagttgtggcaggcggcgagcagggccc
>MSB-158621_fodiens_A
tgcagagccaggcggcagaaggccgtgtgggggaaggtcacccgcctgtcccgagccatgcggctactgggttccgaaggggctgttgccccggggcaccagcttcag
gaaggggcggcagtgctgggtgcgaggaaaccccagtcggtgagggagctgagcccagtaggttaggtcacgcccccagttgtggcaggcggcgagcagggccc
>MSB-158621_fodiens_B
tgcagagccaggcggcagaaggccgtgtgggggaaggtcacccgcctgtcccgagccatgcggctactgggttccgaaggggctgttgccccggggcaccagcttcag
gaaggggcggcagtgctgggtgcgaggaaaccccagtcggtgagggagctgagcccagtaggttaggtcacgcccccagttgtggcaggcggcgagcagggccc

```
>MSB-288577_fodiens_A
tgcagagccaggcggcagaaggccgtgtgggggaaggtcacccgcctgtcccgagccatgcggctactgggttccgaaggggctgttgccccggggcaccagcttcag
gaaggggcggcagtgctgggtgcgaggaaaccccagtcggtgagggagctgagcccagtaggttaggtcacgcccccagttgtggcaggcggcgagcagggccc
>MSB-288577_fodiens_B
tgcagagccaggcggcagaaggccgtgtgggggaaggtcacccgcctgtcccgagccatgcggctactgggttccgaaggggctgttgccccggggcaccagcttcag
gaaggggcggcagtgctgggtgcgaggaaaccccagtcggtgagggagctgagcccagtaggttaggtcacgcccccagttgtggcaggcggcgagcagggccc
>MSB-288698_fodiens_A
tgcagagccaggcggcagaaggccgtgtgggggaaggtcacccgcctgtcccgagccatgcggctactgggttccgaaggggctgttgccccggggcaccagcttcag
gaaggggcggcagtgctgggtgcgaggaaaccccagtcggtgagggagctgagcccagtaggttaggtcacgcccccagttgtggcaggcggcgagcagggccc
>MSB-288698_fodiens_B
tgcagagccaggcggcagaaggccgtgtgggggaaggtcacccgcctgtcccgagccatgcggctactgggttccgaaggggctgttgccccggggcaccagcttcag
gaaggggcggcagtgctgggtgcgaggaaaccccagtcggtgagggagctgagcccagtaggttaggtcacgcccccagttgtggcaggcggcgagcagggccc
>MSB-293526_fodiens_A
tgcagagccaggcggcagaaggccgtgtgggggaaggtcacccgcctgtcccgagccatgcggctactgggttccgaaggggctgttgccccggggcaccagcttcag
gaaggggcggcagtgctgggtgcgaggaaaccccagtcggtgagggagctgagcccagtaggttaggtcacgcccccagttgtggcaggcggcgagcagggccc
>MSB-293526_fodiens_B
tgcagagccaggcggcagaaggccgtgtgggggaaggtcacccgcctgtcccgagccatgcggctactgggttccgaaggggctgttgccccggggcaccagcttcag
gaaggggcggcagtgctgggtgcgaggaaaccccagtcggtgagggagctgagcccagtaggttaggtcacgcccccagttgtggcaggcggcgagcagggccc
>MSB-95472_fodiens_A
tgcagagccaggcggcagaaggccgtgtgggggaaggtcacccgcctgtcccgagccatgcggctactgggttccgaaggggctgttgccccggggcaccagcttcag
gaaggggcggcagtgctgggtgcgaggaaaccccagtcggtgagggagctgagcccagtaggttaggtcacgcccccagttgtggcaggcggcgagcagggccc
>MSB-95472_fodiens_B
tgcagagccaggcggcagaaggccgtgtgggggaaggtcacccgcctgtcccgagccatgcggctactgggttccgaaggggctgttgccccggggcaccagcttcag
gaaggggcggcagtgctgggtgcgaggaaaccccagtcggtgagggagctgagcccagtaggttaggtcacgcccccagttgtggcaggcggcgagcagggccc
>IBE-C4515_anomalus_A
tgcagagcccggcggcagaaggccgtgtgggggaaggtcacccgcctgtcccgagccatgcggctactgggttccgaaggggctgttgccccggggcaccagcttcag
gaaggggcggcagtgctgggtgcgaggaacccccagtcggtgagggagctgagcccagtaggttaggtcacgcccccagttgtggcaggcggcgcgcagggccc
>IBE-C4515_anomalus_B
tgcagagcccggcggcagaaggccgtgtgggggaaggtcacccgcctgtcccgagccatgcggctactgggttccgaaggggctgttgccccggggcaccagcttcag
gaaggggcggcagtgctgggtgcgaggaacccccagtcggtgagggagctgagcccagtaggttaggtcacgcccccagttgtggcaggcggcgcgcagggccc
```

CSF2-intron-2-oneseq.fasta
>IBE-C1529_anomalus_A
tttgttcatgacacaggggctgtgacatgagcatttattgttgaaagacagtggctatagaggggcacagtgagattcaaggaatgatcattgctcacatagctctgg
gcacaccttttccctaaatgacctctgtttctggggtttgggacctttttaaaataccagagcaaaggaatatgtgctatctaaggggcctgatctgagcagtg
>IBE-C1529_anomalus_B
tttgttcatgacacaggggctgtgacatgagcatttattgttgaaagacagtggctatagaggggcacagtgagattcaaggaatgatcattgctcacatagctctgg
gcacaccttttccctaaatgacctctgtttctggggtttgggacctttttaaaataccagagcaaaggaatatgtgctatctaaggggcctgatctgagcagtg
>IBE-C1789_anomalus_A
tttgttcatgacacaggggctgtgacatgagcatttattgttgaaagacagtggctatagaggggcacagtgagattcaaggaatgatcattgctcacatagctctgg
gcacaccttttccctaaatgacctctgtttctggggtttgggacctttttaaaataccagagcaaaggaatatgtgctatctaaggggcctgatctgagcagtg
>IBE-C1789_anomalus_B
tttgttcatgacacaggggctgtgacatgagcatttattgttgaaagacagtggctatagaggggcacagtgagattcaaggaatgatcattgctcacatagctctgg
gcacaccttttccctaaatgacctctgtttctggggtttgggacctttttaaaataccagagcaaaggaatatgtgctatctaaggggcctgatctgagcagtg
>IBE-C1808_milleri_A
tttgttcatgacacaggggctgtaacatgagcatttattgttgaaagacagtggctatagaggggcacagtgagattcaaggaatgatcatcgctcacatagctctgg
gcacaccttttccctaaatgacctctgtttctgggatttgggacctttttaaaataccagagcaaaggaatatgtgctatctaaggggcctgatctgagctgtg
>IBE-C1808_milleri_B
tttgttcatgacacaggggctgtaacatgagcatttattgttgaaagacagtggctatagaggggcacagtgagattcaaggaatgatcatcgctcacatagctctgg
gcacaccttttccctaaatgacctctgtttctgggatttgggacctttttaaaataccagagcaaaggaatatgtgctatctaaggggcctgatctgagctgtg
>IBE-C2895_anomalus_A
tttgttcatgacacaggggctgtgacatgagcatttattgttgaaagacagtggctatagaggggcacagtgagattcaaggaatgatcattgctcacatagctctgg
gcacaccttttccctaaatgacctctgtttctggggtttgggacctttttaaaataccagagcaaaggaatatgtgctatctaaggggcctgatctgagcagtg
>IBE-C2895_anomalus_B
tttgttcatgacacaggggctgtgacatgagcatttattgttgaaagacagtggctatagaggggcacagtgagattcaaggaatgatcattgctcacatagctctgg
gcacaccttttccctaaatgacctctgtttctggggtttgggacctttttaaaataccagagcaaaggaatatgtgctatctaaggggcctgatctgagcagtg
>IBE-C3786_milleri_A
tttgttcatgacacaggggctgtaacatgagcatttattgttgaaagacagtggctatagaggggcacagtgagattcaaggaatgatcatcgctcacatagctctgg
gcacaccttttccctaaatgacctctgtttctgggatttgggacctttttaaaataccagagcaaaggaatatgtgctatctaaggggcctgatctgagctgtg
>IBE-C3786_milleri_B
tttgttcatgacacaggggctgtaacatgagcatttattgttgaaagacagtggctatagaggggcacagtgagattcaaggaatgatcatcgctcacatagctctgg
gcacaccttttccctaaatgacctctgtttctgggatttgggacctttttaaaataccagagcaaaggaatatgtgctatctaaggggcctgatctgagctgtg
>IBE-C4116_milleri_A
tttgttcatgacacaggggctgtaacatgagcatttattgttgaaagacagtggctatagaggggcacagtgagattcaaggaatgatcatcgctcacatagctctgg
gcacaccttttccctaaatgacctctgtttctgggatttgggacctttttaaaataccagagcaaaggaatatgtgctatctaaggggcctgatctgagctgtg
>IBE-C4116_milleri_B
tttgttcatgacacaggggctgtaacatgagcatttattgttgaaagacagtggctatagaggggcacagtgagattcaaggaatgatcatcgctcacatagctctgg
gcacaccttttccctaaatgacctctgtttctgggatttgggacctttttaaaataccagagcaaaggaatatgtgctatctaaggggcctgatctgagctgtg
>IBE-C101_fodiens_A
tttgttcatgagacaggggctgtgacatgagcatttattgttgaaagacagtggctatagaggggcacagtcagattcaaggaatgatcattgctcacatagctccgg
gcacaccttttccctaaatgacctctgtttctggggtttgggaccttcttaaaataccagggcaaaggaatatgtgctatctgaggggcttgatctgagcagtg
>IBE-C101_fodiens_B
tttgttcatgagacaggggctgtgacatgagcatttattgttgaaagacagtggctatagaggggcacagtcagattcaaggaatgatcattgctcacatagctccgg
gcacaccttttccctaaatgacctctgtttctggggtttgggaccttcttaaaataccagggcaaaggaatatgtgctatctgaggggcttgatctgagcagtg
>IBE-C1914_fodiens_A
tttgttcatgagacaggggctgtgacatgagcatttattgttgaaagacagtggctatagaggggcacagtcagattcaaggaatgatcattgctcacatagctccgg
gcacaccttttccctaaatgacctctgtttctggggtttgggaccttcttaaaataccagggcaaaggaatatgtgctatctgaggggcttgatctgagcagtg
>IBE-C1914_fodiens_B
tttgttcatgagacaggggctgtgacatgagcatttattgttgaaagacagtggctatagaggggcacagtcagattcaaggaatgatcattgctcacatagctccgg
gcacaccttttccctaaatgacctctgtttctggggtttgggaccttcttaaaataccagggcaaaggaatatgtgctatctgaggggcttgatctgagcagtg
>IBE-C4120_teres_A
tttgttcatgacacaggggctgtaacatgagcatttattgttgaaagacagtggctatagaggggcacagtgagactcaaggaatgatcatcgctcacatagctctgg
gcacaccttttccctaaatgacctctgtttctggggtttgggacctttttaaaataccagagcaaaggaatatgtgctatctaaggggcctgatctgagctgtg
>IBE-C4120_teres_B
tttgttcatgacacaggggctgtaacatgagcatttattgttgaaagacagtggctatagaggggcacagtgagactcaaggaatggtcatcgctcacatagctctgg
gcacaccttttccctaaatgacctctgtttctggggtttgggacctttttaaaataccagagcaaaggaatatgtgctatctaaggggcctgatctgagctgtg
>IBE-C4122_teres_A
tttgttcatgacacaggggctgtaacatgagcatttattgttgaaagacagtggctatagaggggcacagtgagactcaaggaatgatcatcgctcacatagctctgg
gcacaccttttccctaaatgacctctgtttctggggtttgggacctttttaaaataccagagcaaaggaatatgtgctatctaaggggcctgatctgagctgtg
>IBE-C4122_teres_B
tttgttcatgacacaggggctgtaacatgagcatttattgttgaaagacagtggctatagaggggcacagtgagactcaaggaatggtcatcgctcacatagctctgg
gcacaccttttccctaaatgacctctgtttctggggtttgggacctttttaaaataccagagcaaaggaatatgtgctatctaaggggcctgatctgagctgtg
>IBE-C5182_niethammeri_A
tttgttcatgagacaggggctgtgacatgagcatttattgttgaaagacagtggctatagaggggcacagtcagattcaaggaatgatcattgctcacatagctccgg
gcacaccttttccctaaatgacctctgtttctggggtttgggaccttcttaaaataccagggcaaaggaatatgtgctatctgaggggcttgatctgagcagtg
>IBE-C5182_niethammeri_B
tttgttcatgagacaggggctgtgacatgagcatttattgttgaaagacagtggctatagaggggcacagtcagattcaaggaatgatcattgctcacatagctccgg
gcacaccttttccctaaatgacctctgtttctggggtttgggaccttcttaaaataccagggcaaaggaatatgtgctatctgaggggcttgatctgagcagtg
>IBE-C5368_niethammeri_A
tttgttcatgagacaggggctgtgacatgagcatttattgttgaaagacagtggctatagaggggcacagtcagattcaaggaatgatcattgctcacatagctccgg
gcacaccttttccctaaatgacctctgtttctggggtttgggaccttcttaaaataccagggcaaaggaatatgtgctatctgaggggcttgatctgagcagtg
>IBE-C5368_niethammeri_B
tttgttcatgagacaggggctgtgacatgagcatttattgttgaaagacagtggctatagaggggcacagtcagattcaaggaatgatcattgctcacatagctccgg
gcacaccttttccctaaatgacctctgtttctggggtttgggaccttcttaaaataccagggcaaaggaatatgtgctatctgaggggcttgatctgagcagtg
>IBE-C5697_niethammeri_A

tttgttcatgagacaggggctgtgacatgagcatttattgttgaaagacagtggctatagaggggcacagtcagattcaaggaatgatcattgctcacatagctccgg
gcacaccttttccctaaatgacctctgtttctggggtttgggaccttcttaaaataccagggcaaaggaatatgtgctatctgaggggcttgatctgagcagtg
>IBE-C5697_niethammeri_B
tttgttcatgagacaggggctgtgacatgagcatttattgttgaaagacagtggctatagaggggcacagtcagattcaaggaatgatcattgctcacatagctccgg
gcacaccttttccctaaatgacctctgtttctggggtttgggaccttcttaaaataccagggcaaaggaatatgtgctatctgaggggcttgatctgagcagtg
>IBE-C5700_anomalus_A
tttgttcatgacacaggggctgtgacatgagcatttattgttgaaagacagtggctatagaggggcacagtgagattcaaggaatgatcattgctcacatagctctgg
gcacaccttttccctaaatgacctctgtttctggggtttgggacctttttaaaataccagagcaaaggaatatgtgctatctaaggggcctgatctgagcagtg
>IBE-C5700_anomalus_B
tttgttcatgacacaggggctgtgacatgagcatttattgttgaaagacagtggctatagaggggcacagtgagattcaaggaatgatcattgctcacatagctctgg
gcacaccttttccctaaatgacctctgtttctggggtttgggacctttttaaaataccagagcaaaggaatatgtgctatctaaggggcctgatctgagcagtg
>IBE-C5701_niethammeri_A
tttgttcatgagacaggggctgtgacatgagcatttattgttgaaagacagtggctatagaggggcacagtcagattcaaggaatgatcattgctcacatagctccgg
gcacaccttttccctaaatgacctctgtttctggggtttgggaccttcttaaaataccagggcaaaggaatatgtgctatctgaggggcttgatctgagcagtg
>IBE-C5701_niethammeri_B
tttgttcatgagacaggggctgtgacatgagcatttattgttgaaagacagtggctatagaggggcacagtcagattcaaggaatgatcattgctcacatagctccgg
gcacaccttttccctaaatgacctctgtttctggggtttgggaccttcttaaaataccagggcaaaggaatatgtgctatctgaggggcttgatctgagcagtg
>IBE-C1927_milleri_A
tttgttcatgacacaggggctgtaacatgagcatttattgttgaaagacagtggctatagaggggcacagtgagattcaaggaatgatcatcgctcacatagctctgg
gcacaccttttccctaaatgacctctgtttctgggatttgggacctttttaaaataccagagcaaaggaatatgtgctatctaaggggcctgatctgagctgtg
>IBE-C1927_milleri_B
tttgttcatgacacaggggctgtaacatgagcatttattgttgaaagacagtggctatagaggggcacagtgagattcaaggaatgatcatcgctcacatagctctgg
gcacaccttttccctaaatgacctctgtttctgggatttgggacctttttaaaataccagagcaaaggaatatgtgctatctaaggggcctgatctgagctgtg
>IBE-C5367_fodiens_A
tttgttcatgagacaggggctgtgacatgagcatttattgttgaaagacagtggctatagaggggcacagtcagattcaaggaatgatcattgctcacatagctccgg
gcacaccttttccctaaatgacctctgtttctggggtttgggaccttcttaaaataccagggcaaaggaatatgtgctatctgaggggcttgatctgagcagtg
>IBE-C5367_fodiens_B
tttgttcatgagacaggggctgtgacatgagcatttattgttgaaagacagtggctatagaggggcacagtcagattcaaggaatgatcattgctcacatagctccgg
gcacaccttttccctaaatgacctctgtttctggggtttgggaccttcttaaaataccagggcaaaggaatatgtgctatctgaggggcttgatctgagcagtg
>IBE-C5370_fodiens_A
tttgttcatgagacaggggctgtgacatgagcatttattgttgaaagacagtggctatagaggggcacagtcagattcaaggaatgatcattgctcacatagctccgg
gcacaccttttccctaaatgacctctgtttctggggtttgggaccttcttaaaataccagggcaaaggaatatgtgctatctgaggggcttgatctgagcagtg
>IBE-C5370_fodiens_B
tttgttcatgagacaggggctgtgacatgagcatttattgttgaaagacagtggctatagaggggcacagtcagattcaaggaatgatcattgctcacatagctccgg
gcacaccttttccctaaatgacctctgtttctggggtttgggaccttcttaaaataccagggcaaaggaatatgtgctatctgaggggcttgatctgagcagtg
>IBE-C5383_anomalus_A
tttgttcatgacacaggggctgtgacatgagcatttattgttgaaagacagtggctatagaggggcacagtgagattcaaggaatgatcattgctcacatagctctgg
gcacaccttttccctaaatgacctctgtttctggggtttgggacctttttaaaataccagagcaaaggaatatgtgctatctaaggggcctgatctgagcagtg
>IBE-C5383_anomalus_B
tttgttcatgacacaggggctgtgacatgagcatttattgttgaaagacagtggctatagaggggcacagtgagattcaaggaatgatcattgctcacatagctctgg
gcacaccttttccctaaatgacctctgtttctggggtttgggacctttttaaaataccagagcaaaggaatatgtgctatctaaggggcctgatctgagcagtg
>IBE-C5603_niethammeri_A
tttgttcatgagacaggggctgtgacatgagcatttattgttgaaagacagtggctatagaggggcacagtcagattcaaggaatgatcattgctcacatagctccgg
gcacaccttttccctaaatgacctctgtttctggggtttgggaccttcttaaaataccagggcaaaggaatatgtgctatctgaggggcttgatctgagcagtg
>IBE-C5603_niethammeri_B
tttgttcatgagacaggggctgtgacatgagcatttattgttgaaagacagtggctatagaggggcacagtcagattcaaggaatgatcattgctcacatagctccgg
gcacaccttttccctaaatgacctctgtttctggggtttgggaccttcttaaaataccagggcaaaggaatatgtgctatctgaggggcttgatctgagcagtg
>IBE-C5604_niethammeri_A
tttgttcatgagacaggggctgtgacatgagcatttattgttgaaagacagtggctatagaggggcacagtcagattcaaggaatgatcattgctcacatagctccgg
gcacaccttttccctaaatgacctctgtttctggggtttgggaccttcttaaaataccagggcaaaggaatatgtgctatctgaggggcttgatctgagcagtg
>IBE-C5604_niethammeri_B
tttgttcatgagacaggggctgtgacatgagcatttattgttgaaagacagtggctatagaggggcacagtcagattcaaggaatgatcattgctcacatagctccgg
gcacaccttttccctaaatgacctctgtttctggggtttgggaccttcttaaaataccagggcaaaggaatatgtgctatctgaggggcttgatctgagcagtg
>IBE-C5719_anomalus_A
tttgttcatgacacaggggctgtgacatgagcatttattgttgaaagacagtggctatagaggggcacagtgagattcaaggaatgatcattgctcacatagctctgg
gcacaccttttccctaaatgacctctgtttctggggtttgggacctttttaaaataccagagcaaaggaatatgtgctatctaaggggcctgatctgagcagtg
>IBE-C5719_anomalus_B
tttgttcatgacacaggggctgtgacatgagcatttattgttgaaagacagtggctatagaggggcacagtgagattcaaggaatgatcattgctcacatagctctgg
gcacaccttttccctaaatgacctctgtttctggggtttgggacctttttaaaataccagagcaaaggaatatgtgctatctaaggggcctgatctgagcagtg
>IBE-C5720_anomalus_A
tttgttcatgacacaggggctgtgacatgagcatttattgttgaaagacagtggctatagaggggcacagtgagattcaaggaatgatcattgctcacatagctctgg
gcacaccttttccctaaatgacctctgtttctggggtttgggacctttttaaaataccagagcaaaggaatatgtgctatctaaggggcctgatctgagcagtg
>IBE-C5720_anomalus_B
tttgttcatgacacaggggctgtgacatgagcatttattgttgaaagacagtggctatagaggggcacagtgagattcaaggaatgatcattgctcacatagctctgg
gcacaccttttccctaaatgacctctgtttctggggtttgggacctttttaaaataccagagcaaaggaatatgtgctatctaaggggcctgatctgagcagtg
>IBE-C5699_niethammeri_A
tttgttcatgagacaggggctgtgacatgagcatttattgttgaaagacagtggctatagaggggcacagtcagattcaaggaatgatcattgctcacatagctccgg
gcacaccttttccctaaatgacctctgtttctggggtttgggaccttcttaaaataccagggcaaaggaatatgtgctatctgaggggcttgatctgagcagtg
>IBE-C5699_niethammeri_B
tttgttcatgagacaggggctgtgacatgagcatttattgttgaaagacagtggctatagaggggcacagtcagattcaaggaatgatcattgctcacatagctccgg
gcacaccttttccctaaatgacctctgtttctggggtttgggaccttcttaaaataccagggcaaaggaatatgtgctatctgaggggcttgatctgagcagtg
>IBE-C5741_niethammeri_A
tttgttcatgagacaggggctgtgacatgagcatttattgttgaaagacagtggctatagaggggcacagtcagattcaaggaatgatcattgctcacatagctccgg
gcacaccttttccctaaatgacctctgtttctggggtttgggaccttcttaaaataccagggcaaaggaatatgtgctatctgaggggcttgatctgagcagtg

>IBE-C5741_niethammeri_B
tttgttcatgagacaggggctgtgacatgagcatttattgttgaaagacagtggctatagaggggcacagtcagattcaaggaatgatcattgctcacatagctccgg
gcacaccttttccctaaatgacctctgtttctggggtttgggaccttcttaaaataccagggcaaaggaatatgtgctatctgaggggcttgatctgagcagtg
>UAM-24769_fodiens_A
tttgttcatgagacaggggctgtgacatgagcatttattgttgaaagacagtggctatagaggggcacagtcagattcaaggaatgatcattgctcacatagctccgg
gcacaccttttccctaaatgacctctgtttctggggtttgggaccttcttaaaataccagggcaaaggaatatgtgctatctgaggggcttgatctgagcagtg
>UAM-24769_fodiens_B
tttgttcatgagacaggggctgtgacatgagcatttattgttgaaagacagtggctatagaggggcacagtcagattcaaggaatgatcattgctcacatagctccgg
gcacaccttttccctaaatgacctctgtttctggggtttgggaccttcttaaaataccagggcaaaggaatatgtgctatctgaggggcttgatctgagcagtg
>UAM-24770_fodiens_A
tttgttcatgagacaggggctgtgacatgagcatttattgttgaaagacagtggctatagaggggcacagtcagattcaaggaatgatcattgctcacatagctccgg
gcacaccttttccctaaatgacctctgtttctggggtttgggaccttcttaaaataccagggcaaaggaatatgtgctatctgaggggcttgatctgagcagtg
>UAM-24770_fodiens_B
tttgttcatgagacaggggctgtgacatgagcatttattgttgaaagacagtggctatagaggggcacagtcagattcaaggaatgatcattgctcacatagctccgg
gcacaccttttccctaaatgacctctgtttctggggtttgggaccttcttaaaataccagggcaaaggaatatgtgctatctgaggggcttgatctgagcagtg
>IBE-C5372_niethammeri_A
tttgttcatgagacaggggctgtgacatgagcatttattgttgaaagacagtggctatagaggggcacagtcagattcaaggaatgatcattgctcacatagctccgg
gcacaccttttccctaaatgacctctgtttctggggtttgggaccttcttaaaataccagggcaaaggaatatgtgctatctgaggggcttgatctgagcagtg
>IBE-C5372_niethammeri_B
tttgttcatgagacaggggctgtgacatgagcatttattgttgaaagacagtggctatagaggggcacagtcagattcaaggaatgatcattgctcacatagctccgg
gcacaccttttccctaaatgacctctgtttctggggtttgggaccttcttaaaataccagggcaaaggaatatgtgctatctgaggggcttgatctgagcagtg
>IBE-C5374_fodiens_A
tttgttcatgagacaggggctgtgacatgagcatttattgttgaaagacagtggctatagaggggcacagtcagattcaaggaatgatcattgctcacatagctccgg
gcacaccttttccctaaatgacctctgtttctggggtttgggaccttcttaaaataccagggcaaaggaatatgtgctatctgaggggcttgatctgagcagtg
>IBE-C5374_fodiens_B
tttgttcatgagacaggggctgtgacatgagcatttattgttgaaagacagtggctatagaggggcacagtcagattcaaggaatgatcattgctcacatagctccgg
gcacaccttttccctaaatgacctctgtttctggggtttgggaccttcttaaaataccagggcaaaggaatatgtgctatctgaggggcttgatctgagcagtg
>IBE-C5376_fodiens_A
tttgttcatgagacaggggctgtgacatgagcatttattgttgaaagacagtggctatagaggggcacagtcagattcaaggaatgatcattgctcacatagctccgg
gcacaccttttccctaaatgacctctgtttctggggtttgggaccttcttaaaataccagggcaaaggaatatgtgctatctgaggggcttgatctgagcagtg
>IBE-C5376_fodiens_B
tttgttcatgagacaggggctgtgacatgagcatttattgttgaaagacagtggctatagaggggcacagtcagattcaaggaatgatcattgctcacatagctccgg
gcacaccttttccctaaatgacctctgtttctggggtttgggaccttcttaaaataccagggcaaaggaatatgtgctatctgaggggcttgatctgagcagtg
>IBE-C5726_anomalus_A
tttgttcatgacacaggggctgtgacatgagcatttattgttgaaagacagtggctatagaggggcacagtgagattcaaggaatgatcattgctcacatagctctgg
gcacaccttttccctaaatgacctctgtttctggggtttgggacctttttaaaataccagagcaaaggaatatgtgctatctaaggggcctgatctgagcagtg
>IBE-C5726_anomalus_B
tttgttcatgacacaggggctgtgacatgagcatttattgttgaaagacagtggctatagaggggcacagtgagattcaaggaatgatcattgctcacatagctctgg
gcacaccttttccctaaatgacctctgtttctggggtttgggacctttttaaaataccagagcaaaggaatatgtgctatctaaggggcctgatctgagcagtg
>IBE-C5742_fodiens_A
tttgttcatgagacaggggctgtgacatgagcatttattgttgaaagacagtggctatagaggggcacagtcagattcaaggaatgatcattgctcacatagctccgg
gcacaccttttccctaaatgacctctgtttctggggtttgggaccttcttaaaataccagggcaaaggaatatgtgctatctgaggggcttgatctgagcagtg
>IBE-C5742_fodiens_B
tttgttcatgagacaggggctgtgacatgagcatttattgttgaaagacagtggctatagaggggcacagtcagattcaaggaatgatcattgctcacatagctccgg
gcacaccttttccctaaatgacctctgtttctggggtttgggaccttcttaaaataccagggcaaaggaatatgtgctatctgaggggcttgatctgagcagtg
>IBE-C5744_fodiens_A
tttgttcatgagacaggggctgtgacatgagcatttattgttgaaagacagtggctatagaggggcacagtcagattcaaggaatgatcattgctcacatagctccgg
gcacaccttttccctaaatgacctctgtttctggggtttgggaccttcttaaaataccagggcaaaggaatatgtgctatctgaggggcttgatctgagcagtg
>IBE-C5744_fodiens_B
tttgttcatgagacaggggctgtgacatgagcatttattgttgaaagacagtggctatagaggggcacagtcagattcaaggaatgatcattgctcacatagctccgg
gcacaccttttccctaaatgacctctgtttctggggtttgggaccttcttaaaataccagggcaaaggaatatgtgctatctgaggggcttgatctgagcagtg
>IBE-C6063_fodiens_A
tttgttcatgagacaggggctgtgacatgagcatttattgttgaaagacagtggctatagaggggcacagtcagattcaaggaatgatcattgctcacatagctccgg
gcacaccttttccctaaatgacctctgtttctggggtttgggaccttcttaaaataccagggcaaaggaatatgtgctatctgaggggcttgatctgagcagtg
>IBE-C6063_fodiens_B
tttgttcatgagacaggggctgtgacatgagcatttattgttgaaagacagtggctatagaggggcacagtcagattcaaggaatgatcattgctcacatagctccgg
gcacaccttttccctaaatgacctctgtttctggggtttgggaccttcttaaaataccagggcaaaggaatatgtgctatctgaggggcttgatctgagcagtg
>IBE-C6064_fodiens_A
tttgttcatgagacaggggctgtgacatgagcatttattgttgaaagacagtggctatagaggggcacagtcagattcaaggaatgatcattgctcacatagctccgg
gcacaccttttccctaaatgacctctgtttctggggtttgggaccttcttaaaataccagggcaaaggaatatgtgctatctgaggggcttgatctgagcagtg
>IBE-C6064_fodiens_B
tttgttcatgagacaggggctgtgacatgagcatttattgttgaaagacagtggctatagaggggcacagtcagattcaaggaatgatcattgctcacatagctccgg
gcacaccttttccctaaatgacctctgtttctggggtttgggaccttcttaaaataccagggcaaaggaatatgtgctatctgaggggcttgatctgagcagtg
>IBE-C6065_anomalus_A
tttgttcatgacacaggggctgtgacatgagcatttattgttgaaagacagtggctatagaggggcacagtgagattcaaggaatgatcattgctcacatagctctgg
gcacaccttttccctaaatgacctctgtttctggggtttgggacctttttaaaataccagagcaaaggaatatgtgctatctaaggggcctgatctgagcagtg
>IBE-C6065_anomalus_B
tttgttcatgacacaggggctgtgacatgagcatttattgttgaaagacagtggctatagaggggcacagtgagattcaaggaatgatcattgctcacatagctctgg
gcacaccttttccctaaatgacctctgtttctggggtttgggacctttttaaaataccagagcaaaggaatatgtgctatctaaggggcctgatctgagcagtg
>IBE-C6069_fodiens_A
tttgttcatgagacaggggctgtgacatgagcatttattgttgaaagacagtggctatagaggggcacagtcagattcaaggaatgatcattgctcacatagctccgg
gcacaccttttccctaaatgacctctgtttctggggtttgggaccttcttaaaataccagggcaaaggaatatgtgctatctgaggggcttgatctgagcagtg
>IBE-C6069_fodiens_B

tttgttcatgagacaggggctgtgacatgagcatttattgttgaaagacagtggctatagaggggcacagtcagattcaaggaatgatcattgctcacatagctccgg
gcacaccttttccctaaatgacctctgtttctggggtttgggaccttcttaaaataccagggcaaaggaatatgtgctatctgaggggcttgatctgagcagtg
>IBE-C4117_fodiens_A
tttgttcatgagacaggggctgtgacatgagcatttattgttgaaagacagtggctatagaggggcacagtcagattcaaggaatgatcattgctcacatagctccgg
gcacaccttttccctaaatgacctctgtttctggggtttgggaccttcttaaaataccagggcaaaggaatatgtgctatctgaggggcttgatctgagcagtg
>IBE-C4117_fodiens_B
tttgttcatgagacaggggctgtgacatgagcatttattgttgaaagacagtggctatagaggggcacagtcagattcaaggaatgatcattgctcacatagctccgg
gcacaccttttccctaaatgacctctgtttctggggtttgggaccttcttaaaataccagggcaaaggaatatgtgctatctgaggggcttgatctgagcagtg
>IBE-C6105_niethammeri_A
tttgttcatgagacaggggctgtgacatgagcatttattgttgaaagacagtggctatagaggggcacagtcagattcaaggaatgatcattgctcacatagctccgg
gcacaccttttccctaaatgacctctgtttctggggtttgggaccttcttaaaataccagggcaaaggaatatgtgctatctgaggggcttgatctgagcagtg
>IBE-C6105_niethammeri_B
tttgttcatgagacaggggctgtgacatgagcatttattgttgaaagacagtggctatagaggggcacagtcagattcaaggaatgatcattgctcacatagctccgg
gcacaccttttccctaaatgacctctgtttctggggtttgggaccttcttaaaataccagggcaaaggaatatgtgctatctgaggggcttgatctgagcagtg
>IBE-C6106_niethammeri_A
tttgttcatgagacaggggctgtgacatgagcatttattgttgaaagacagtggctatagaggggcacagtcagattcaaggaatgatcattgctcacatagctctgg
gcacaccttttccctaaatgacctctgtttctggggtttgggaccttcttaaaataccagggcaaaggaatatgtgctatctgaggggcttgatctgagcagtg
>IBE-C6106_niethammeri_B
tttgttcatgagacaggggctgtgacatgagcatttattgttgaaagacagtggctatagaggggcacagtcagattcaaggaatgatcattgctcacatagctctgg
gcacaccttttccctaaatgacctctgtttctggggtttgggaccttcttaaaataccagggcaaaggaatatgtgctatctgaggggcttgatctgagcagtg
>IBE-C6108_niethammeri_A
tttgttcatgagacaggggctgtgacatgagcatttattgttgaaagacagtggctatagaggggcacagtcagattcaaggaatgatcattgctcacatagctccgg
gcacaccttttccctaaatgacctctgtttctggggtttgggaccttcttaaaataccagggcaaaggaatatgtgctatctgaggggcttgatctgagcagtg
>IBE-C6108_niethammeri_B
tttgttcatgagacaggggctgtgacatgagcatttattgttgaaagacagtggctatagaggggcacagtcagattcaaggaatgatcattgctcacatagctccgg
gcacaccttttccctaaatgacctctgtttctggggtttgggaccttcttaaaataccagggcaaaggaatatgtgctatctgaggggcttgatctgagcagtg
>IBE-C6118_anomalus_A
tttgttcatgacacaggggctgtgacatgagcatttattgttgaaagacagtggctatagaggggcacagtgagattcaaggaatgatcattgctcacatagctctgg
gcacaccttttccctaaatgacctctgtttctggggtttgggacctttttaaaataccagagcaaaggaatatgtgctatctaaggggcctgatctgagcagtg
>IBE-C6118_anomalus_B
tttgttcatgacacaggggctgtgacatgagcatttattgttgaaagacagtggctatagaggggcacagtgagattcaaggaatgatcattgctcacatagctctgg
gcacaccttttccctaaatgacctctgtttctggggtttgggacctttttaaaataccagagcaaaggaatatgtgctatctaaggggcctgatctgagcagtg
>IBE-C6121_anomalus_A
tttgttcatgacacaggggctgtgacatgagcatttattgttgaaagacagtggctatagaggggcacagtgagattcaaggaatgatcattgctcacatagctctgg
gcacaccttttccctaaatgacctctgtttctggggtttgggacctttttaaaataccagagcaaaggaatatgtgctatctaaggggcctgatctgagcagtg
>IBE-C6121_anomalus_B
tttgttcatgacacaggggctgtgacatgagcatttattgttgaaagacagtggctatagaggggcacagtgagattcaaggaatgatcattgctcacatagctctgg
gcacaccttttccctaaatgacctctgtttctggggtttgggacctttttaaaataccagagcaaaggaatatgtgctatctaaggggcctgatctgagcagtg
>IBE-C6110_niethammeri_A
tttgttcatgagacaggggctgtgacatgagcatttattgttgaaagacagtggctatagaggggcacagtcagattcaaggaatgatcattgctcacatagctccgg
gcacaccttttccctaaatgacctctgtttctggggtttgggaccttcttaaaataccagggcaaaggaatatgtgctatctgaggggcttgatctgagcagtg
>IBE-C6110_niethammeri_B
tttgttcatgagacaggggctgtgacatgagcatttattgttgaaagacagtggctatagaggggcacagtcagattcaaggaatgatcattgctcacatagctccgg
gcacaccttttccctaaatgacctctgtttctggggtttgggaccttcttaaaataccagggcaaaggaatatgtgctatctgaggggcttgatctgagcagtg
>IBE-C6116_niethammeri_A
tttgttcatgagacaggggctgtgacatgagcatttattgttgaaagacagtggctatagaggggcacagtcagattcaaggaatgatcattgctcacatagctccgg
gcacaccttttccctaaatgacctctgtttctggggtttgggaccttcttaaaataccagggcaaaggaatatgtgctatctgaggggcttgatctgagcagtg
>IBE-C6116_niethammeri_B
tttgttcatgagacaggggctgtgacatgagcatttattgttgaaagacagtggctatagaggggcacagtcagattcaaggaatgatcattgctcacatagctccgg
gcacaccttttccctaaatgacctctgtttctggggtttgggaccttcttaaaataccagggcaaaggaatatgtgctatctgaggggcttgatctgagcagtg
>IBE-C6124_anomalus_A
tttgttcatgacacaggggctgtgacatgagcatttattgttgaaagacagtggctatagaggggcacagtgagattcaaggaatgatcattgctcacatagctctgg
gcacaccttttccctaaatgacctctgtttctggggtttgggacctttttaaaataccagagcaaaggaatatgtgctatctaaggggcctgatctgagcagtg
>IBE-C6124_anomalus_B
tttgttcatgacacaggggctgtgacatgagcatttattgttgaaagacagtggctatagaggggcacagtgagattcaaggaatgatcattgctcacatagctctgg
gcacaccttttccctaaatgacctctgtttctggggtttgggacctttttaaaataccagagcaaaggaatatgtgctatctaaggggcctgatctgagcagtg
>IBE-C6125_anomalus_A
tttgttcatgacacaggggctgtgacatgagcatttattgttgaaagacagtggctatagaggggcacagtgagattcaaggaatgatcattgctcacatagctctgg
gcacaccttttccctaaatgacctctgtttctggggtttgggacctttttaaaataccagagcaaaggaatatgtgctatctaaggggcctgatctgagcagtg
>IBE-C6125_anomalus_B
tttgttcatgacacaggggctgtgacatgagcatttattgttgaaagacagtggctatagaggggcacagtgagattcaaggaatgatcattgctcacatagctctgg
gcacaccttttccctaaatgacctctgtttctggggtttgggacctttttaaaataccagagcaaaggaatatgtgctatctaaggggcctgatctgagcagtg
>IBE-C5188_fodiens_A
tttgttcatgagacaggggctgtgacatgagcatttattgttgaaagacagtggctatagaggggcacagtcagattcaaggaatgatcattgctcacatagctccgg
gcacaccttttccctaaatgacctctgtttctggggtttgggaccttcttaaaataccagggcaaaggaatatgtgctatctgaggggcttgatctgagcagtg
>IBE-C5188_fodiens_B
tttgttcatgagacaggggctgtgacatgagcatttattgttgaaagacagtggctatagaggggcacagtcagattcaaggaatgatcattgctcacatagctccgg
gcacaccttttccctaaatgacctctgtttctggggtttgggaccttcttaaaataccagggcaaaggaatatgtgctatctgaggggcttgatctgagcagtg
>IBE-C5359_niethammeri_A
tttgttcatgagacaggggctgtgacatgagcatttattgttgaaagacagtggctatagaggggcacagtcagattcaaggaatgatcattgctcacatagctccgg
gcacaccttttccctaaatgacctctgtttctggggtttgggaccttcttaaaataccagggcaaaggaatatgtgctatctgaggggcttgatctgagcagtg
>IBE-C5359_niethammeri_B
tttgttcatgagacaggggctgtgacatgagcatttattgttgaaagacagtggctatagaggggcacagtcagattcaaggaatgatcattgctcacatagctccgg
gcacaccttttccctaaatgacctctgtttctggggtttgggaccttcttaaaataccagggcaaaggaatatgtgctatctgaggggcttgatctgagcagtg

>IBE-C5371_niethammeri_A
tttgttcatgagacaggggctgtgacatgagcatttattgttgaaagacagtggctatagaggggcacagtcagattcaaggaatgatcattgctcacatagctccgg
gcacaccttttccctaaatgacctctgtttctggggtttgggaccttcttaaaataccagggcaaaggaatatgtgctatctgaggggcttgatctgagcagtg
>IBE-C5371_niethammeri_B
tttgttcatgagacaggggctgtgacatgagcatttattgttgaaagacagtggctatagaggggcacagtcagattcaaggaatgatcattgctcacatagctccgg
gcacaccttttccctaaatgacctctgtttctggggtttgggaccttcttaaaataccagggcaaaggaatatgtgctatctgaggggcttgatctgagcagtg
>MSB-158621_fodiens_A
tttgttcatgagacaggggctgtgacatgagcatttattgttgaaagacagtggctatagaggggcacagtcagattcaaggaatgatcattgctcacatagctccgg
gcacaccttttccctaaatgacctctgtttctggggtttgggaccttcttaaaataccagggcaaaggaatatgtgctatctgaggggcttgatctgagcagtg
>MSB-158621_fodiens_B
tttgttcatgagacaggggctgtgacatgagcatttattgttgaaagacagtggctatagaggggcacagtcagattcaaggaatgatcattgctcacatagctccgg
gcacaccttttccctaaatgacctctgtttctggggtttgggaccttcttaaaataccagggcaaaggaatatgtgctatctgaggggcttgatctgagcagtg
>MSB-288577_fodiens_A
tttgttcatgagacaggggctgtgacatgagcatttattgttgaaagacagtggctatagaggggcacagtcagattcaaggaatgatcattgctcacatagctccgg
gcacaccttttccctaaatgacctctgtttctggggtttgggaccttcttaaaataccagggcaaaggaatatgtgctatctgaggggcttgatctgagcagtg
>MSB-288577_fodiens_B
tttgttcatgagacaggggctgtgacatgagcatttattgttgaaagacagtggctatagaggggcacagtcagattcaaggaatgatcattgctcacatagctccgg
gcacaccttttccctaaatgacctctgtttctggggtttgggaccttcttaaaataccagggcaaaggaatatgtgctatctgaggggcttgatctgagcagtg
>MSB-288698_fodiens_A
tttgttcatgagacaggggctgtgacatgagcatttattgttgaaagacagtggctatagaggggcacagtcagattcaaggaatgatcattgctcacatagctccgg
gcacaccttttccctaaatgacctctgtttctggggtttgggaccttcttaaaataccagggcaaaggaatatgtgctatctgaggggcttgatctgagcagtg
>MSB-288698_fodiens_B
tttgttcatgagacaggggctgtgacatgagcatttattgttgaaagacagtggctatagaggggcacagtcagattcaaggaatgatcattgctcacatagctccgg
gcacaccttttccctaaatgacctctgtttctggggtttgggaccttcttaaaataccagggcaaaggaatatgtgctatctgaggggcttgatctgagcagtg
>MSB-293526_fodiens_A
tttgttcatgagacaggggctgtgacatgagcatttattgttgaaagacagtggctatagaggggcacagtcagattcaaggaatgatcattgctcacatagctccgg
gcacaccttttccctaaatgacctctgtttctggggtttgggaccttcttaaaataccagggcaaaggaatatgtgctatctgaggggcttgatctgagcagtg
>MSB-293526_fodiens_B
tttgttcatgagacaggggctgtgacatgagcatttattgttgaaagacagtggctatagaggggcacagtcagattcaaggaatgatcattgctcacatagctccgg
gcacaccttttccctaaatgacctctgtttctggggtttgggaccttcttaaaataccagggcaaaggaatatgtgctatctgaggggcttgatctgagcagtg
>MSB-95472_fodiens_A
tttgttcatgagacaggggctgtgacatgagcatttattgttgaaagacagtggctatagaggggcacagtcagattcaaggaatgatcattgctcacatagctccgg
gcacaccttttccctaaatgacctctgtttctggggtttgggaccttcttaaaataccagggcaaaggaatatgtgctatctgaggggcttgatctgagcagtg
>MSB-95472_fodiens_B
tttgttcatgagacaggggctgtgacatgagcatttattgttgaaagacagtggctatagaggggcacagtcagattcaaggaatgatcattgctcacatagctccgg
gcacaccttttccctaaatgacctctgtttctggggtttgggaccttcttaaaataccagggcaaaggaatatgtgctatctgaggggcttgatctgagcagtg
>MSB-158495_fodiens_A
tttgttcatgagacaggggctgtgacatgagcatttattgttgaaagacagtggctatagaggggcacagtcagattcaaggaatgatcattgctcacatagctccgg
gcacaccttttccctaaatgacctctgtttctggggtttgggaccttcttaaaataccagggcaaaggaatatgtgctatctgaggggcttgatctgagcagtg
>MSB-158495_fodiens_B
tttgttcatgagacaggggctgtgacatgagcatttattgttgaaagacagtggctatagaggggcacagtcagattcaaggaatgatcattgctcacatagctccgg
gcacaccttttccctaaatgacctctgtttctggggtttgggaccttcttaaaataccagggcaaaggaatatgtgctatctgaggggcttgatctgagcagtg
>IBE-C4515_anomalus_A
tttgttcatgacacaggggctgtgacatgagcatttattgttgaaagacagtggctatagaggggcacagtgagattcaaggaatgatcattgctcacatagctctgg
gcacaccttttccctaaatgacctctgtttctggggtttgggacctttttaaaataccagagcaaaggaatatgtgctatctaaggggcctgatctgagcagtg
>IBE-C4515_anomalus_B
tttgttcatgacacaggggctgtgacatgagcatttattgttgaaagacagtggctatagaggggcacagtgagattcaaggaatgatcattgctcacatagctctgg
gcacaccttttccctaaatgacctctgtttctggggtttgggacctttttaaaataccagagcaaaggaatatgtgctatctaaggggcctgatctgagcagtg

GDAP1-intron-1-oneseq.fasta

```
>IBE-C1529_anomalus_A
ccgggccggcgccgccacccttgcaggcctcaaggcaatctcgctccctccagggacgctgcaccttccctggagcttagaggaacatctgagctccaatggctgtga
ccctggtttaccactcttggaagcgacaggaaacatcacttttgccagagggagcgggtggaggccttgtcctgggccgg
>IBE-C1529_anomalus_B
ccgggccggcgccgccacccttgcaggcctcaaggcaatctcgctccctccagggacgctgcaccttccctggagcttagaggaacatctgagctccaatggctgtga
ccctggtttaccactcttggaagcgacaggaaacatcacttttgccagagggagcgggtggaggccttgtcctgggccgg
>IBE-C1789_anomalus_A
ccgggccggcgccgccacccttgcaggcctcaaggcaatctcgctccctccagggacgctgcaccttccctggagcttagaggaacatctgagctccaatggctgtga
ccctggtttaccactcttggaagcgacaggaaacatcacttttgccagagggagcgggtggaggccttgtcctgggccgg
>IBE-C1789_anomalus_B
ccgggccggcgccgccacccttgcaggcctcaaggcaatctcgctccctccagggacgctgcaccttccctggagcttagaggaacatctgagctccaatggctgtga
ccctggtttaccactcttggaagcgacaggaaacatcacttttgccagagggagcgggtggaggccttgtcctgggccgg
>IBE-C1808_milleri_A
ccgggccggcgcggccacccttgcaggcctcaaggcaatctcgctccctccagggacgctgcaccttccctggagcttagaggaacatctgagctccaatggctgtga
ccctggtttgccactcttggaagcgacaggaaacatcacttttgccagagggagcgggtggaggccttgtcctgggccgg
>IBE-C1808_milleri_B
ccgggccggcgcggccacccttgcaggcctcaaggcaatctcgctccctccagggacgctgcaccttccctggagcttagaggaacatctgagctccaatggctgtga
ccctggtttgccactcttggaagcgacaggaaacatcacttttgccagagggagcgggtggaggccttgtcctgggccgg
>IBE-C2895_anomalus_A
ccgggccggcgccgccacccttgcaggcctcaaggcaatctcgctccctccagggacgctgcaccttccctggagcttagaggaacatctgagctccaatggctgtga
ccctggtttaccactcttggaagcgacaggaaacatcacttttgccagagggagcgggtggaggccttgtcctgggccgg
>IBE-C2895_anomalus_B
ccgggccggcgccgccacccttgcaggcctcaaggcaatctcactccctccagggacgctgcaccttccctggagcttagaggaacatctgagctccaatggctgtga
ccctggtttaccactcttggaagcgacaggaaacatcacttttgccagagggagcgggtggaggccttgtcctgggccgg
>IBE-C3786_milleri_A
ccgggccggcgcggccacccttgcaggcctcaaggcaatctcgctccctccagggacgctgcaccttccctggagcttagaggaacatctgagctccaatggctgtga
ccctggtttgccactcttggaagcgacaggaaacatcacttttgccagagggagcgggtggaggccttgtcctgggccgg
>IBE-C3786_milleri_B
ccgggccggcgcggccacccttgcaggcctcaaggcaatctcgctccctccagggacgctgcaccttccctggagcttagaggaacatctgagctccaatggctgtga
ccctggtttgccactcttggaagcgacaggaaacatcacttttgccagagggagcgggtggaggccttgtcctgggccgg
>IBE-C4116_milleri_A
ccgggccggcgctgccacccttgcaggcctcaaggcaatctcgctccctccagggacgctgcaccttccctggagcttagaggaacatctgagctccaatggctgtga
ccctggtttgccactcttggaagcgacaggaaacatcacttttgccagagggagcgggtggaggccttgtcctgggccgg
>IBE-C4116_milleri_B
ccgggccggcgctgccacccttgcaggcctcaaggcaatctcgctccctccagggacgctgcaccttccctggagcttagaggaacatctgagctccaatggctgtga
ccctggtttgccactcttggaagcgacaggaaacatcacttttgccagagggagcgggtggaggccttgtcctgggccgg
>IBE-C101_fodiens_A
ccggggcggcgccgccacccttgcaggcctcaaggcaatctcgctccttccagggacgctgcaccttccctggagcttagaggaagatctgagctccaatggctgtga
ccctggtttgccactcatagaagcgacaggaaacatcacttttgccagacggagcgggtggaggccttgtcctgggccgg
>IBE-C101_fodiens_B
ccggggcggcgccgccacccttgcaggcctcaaggcaatctcgctccttccagggacgctgcaccttccctggagcttagaggaagatctgagctccaatggctgtga
ccctggtttgccactcatagaagcgacaggaaacatcacttttgccagacggagcgggtggaggccttgtcctgggccgg
>IBE-C1914_fodiens_A
ccggggcggcgccgccacccttgcaggcctcaaggcaatctcgctccttccagggacgctgcaccttccctggagcttagaggaagatctgagctccaatggctgtga
ccctggtttgccactcatagaagcgacaggaaacatcacttttgccagacggagcgggtggaggccttgtcctgggccgg
>IBE-C1914_fodiens_B
ccggggcggcgccgccacccttgcaggcctcaaggcaatctcgctccttccagggacgctgcaccttccctggagcttagaggaagatctgagctccaatggctgtga
ccctgctttgccactcatagaagcgacaggaaacatcacttttgccagacggagcgggtggaggccttgtcctgggccgg
>IBE-C4120_teres_A
ccgggccggcgccgccacccttgcaggcctcaaggcaatctcgctccctccagggacgctgcaccttccctggagcttagaggaacatctgagctccaatggctgtga
ccctggtttgccactcttggaagcgacaggaagcatcacttttgccagagggagcgggtggaggccttgtcctgggccgg
>IBE-C4120_teres_B
ccgggccggcgccgccacccttgcaggcctcaaggcaatctcgctccctccagggacgctgcaccttccctggagcttagaggaacatctgagctccaatggctgtga
ccctggtttgccactcttggaagcgacaggaagcatcacttttgccagagggagcgggtggaggccttgtcctgggccgg
>IBE-C4122_teres_A
ccgggccggcgccgccacccttgcaggcctcaaggcaatctcgctccctccagggacgctgcaccttccctggagcttagaggaacatctgagctccaatggctgtga
ccctggtttgccactcttggaagcgacaggaagcatcacttttgccagagggagcgggtggaggccttgtcctgggccgg
>IBE-C4122_teres_B
ccgggccggcgccgccacccttgcaggcctcaaggcaatctcgctccctccagggacgctgcaccttccctggagcttagaggaacatctgagctccaatggctgtga
ccctggtttgccactcttggaagcgacaggaaacatcacttttgccagagggagcgggtggaggccttgtcctgggccgg
>IBE-C1927_milleri_A
ccgggccggcgcggccacccttgcaggcctcaaggcaatctcgctccctccagggacgctgcaccttccctggagcttagaggaacatctgagctccaatggctgtga
ccctggtttgccactcttggaagcgacaggaaacatcacttttgccagagggagcgggtggaggccttgtcctgggccgg
>IBE-C1927_milleri_B
ccgggccggcgcggccacccttgcaggcctcaaggcaatctcgctccctccagggacgctgcaccttccctggagcttagaggaacatctgagctccaatggctgtga
ccctggtttgccactcttggaagcgacaggaaacatcacttttgccagagggagcgggtggaggccttgtcctgggccgg
>IBE-C5182_niethammeri_A
ccggggcggcgccgccacccttgcaggcctcaaggcaatctcgctccttccagggacgctgcaccttccctggagcttagaggaagatctgagctccaatggctgtga
ccctgctttgccactcatagaagcgacaggaaacatcacttttgccagacggagcgggtggaggccttgtcctgggccgg
>IBE-C5182_niethammeri_B
ccggggcggcgccgccacccttgcaggcctcaaggcaatctcgctccttccagggacgctgcaccttccctggagcttagaggaagatctgagctccaatggctgtga
ccctgctttgccactcatagaagcgacaggaaacatcacttttgccagacggagcgggtggaggccttgtcctgggccgg
>IBE-C5367_fodiens_A
```

```
ccggggcggcgccgccacccttgcaggcctcaaggcaatctcgctccttccagggacgctgcaccttccctggagcttagaggaagatctgagctccaatggctgtga
ccctggtttgccactcatagaagcgacaggaaacatcacttttgccagacggagcgggtggaggccttgtcctgggccgg
>IBE-C5367_fodiens_B
ccggggcggcgccgccacccttgcaggcctcaaggcaatctcgctccttccagggacgctgcaccttccctggagcttagaggaagatctgagctccaatggctgtga
ccctggtttgccactcatagaagcgacaggaaacatcacttttgccagacggagcgggtggaggccttgtcctgggccgg
>IBE-C5368_niethammeri_A
ccggggcggcgccgccacccttgcaggcctcaaggcaatctcgctccttccagggacgctgcaccttccctggagcttagaggaagatctgagctccaatggctgtga
ccctgctttgccactcatagaagcgacaggaaacatcacttttgccagacggagcgggtggaggccttgtcctgggccgg
>IBE-C5368_niethammeri_B
ccggggcggcgccgccacccttgcaggcctcaaggcaatctcgctccttccagggacgctgcaccttccctggagcttagaggaagatctgagctccaatggctgtga
ccctgctttgccactcatagaagcgacaggaaacatcacttttgccagacggagcgggtggaggccttgtcctgggccgg
>IBE-C5697_niethammeri_A
ccggggcggcgccgccacccttgcaggcctcaaggcaatctcgctccttccagggacgctgcaccttccctggagcttagaggaagatctgagctccaatggctgtga
ccctgctttgccactcatagaagcgacaggaaacatcacttttgccagacggagcgggtggaggccttgtcctgggccgg
>IBE-C5697_niethammeri_B
ccggggcggcgccgccacccttgcaggcctcaaggcaatctcgctccttccagggacgctgcaccttccctggagcttagaggaagatctgagctccaatggctgtga
ccctgctttgccactcatagaagcgacaggaaacatcacttttgccagacggagcgggtggaggccttgtcctgggccgg
>IBE-C5700_anomalus_A
ccgggccggcgccgccacccttgcaggcctcaaggcaatctcgctccctccagggacgctgcaccttccctggagcttagaggaacatctgagctccaatggctgtga
ccctggtttaccactcttggaagcgacaggaaacatcacttttgccagagggagcgggtggaggccttgtcctgggccgg
>IBE-C5700_anomalus_B
ccgggccggcgccgccacccttgcaggcctcaaggcaatctcgctccctccagggacgctgcaccttccctggagcttagaggaacatctgagctccaatggctgtga
ccctggtttaccactcttggaagcgacaggaaacatcacttttgccagagggagcgggtggaggccttgtcctgggccgg
>IBE-C5701_niethammeri_A
ccggggcggcgccgccacccttgcaggcctcaaggcaatctcgctccttccagggacgctgcaccttccctggagcttagaggaagatctgagctccaatggctgtga
ccctggtttgccactcatagaagcgacaggaaacatcacttttgccagacggagcgggtggaggccttgtcctgggccgg
>IBE-C5701_niethammeri_B
ccggggcggcgccgccacccttgcaggcctcaaggcaatctcgctccttccagggacgctgcaccttccctggagcttagaggaagatctgagctccaatggctgtga
ccctggtttgccactcatagaagcgacaggaaacatcacttttgccagacggagcgggtggaggccttgtcctgggccgg
>IBE-C5370_fodiens_A
ccggggcggcgccgccacccttgcaggcctcaaggcaatctcgctccttccagggacgctgcaccttccctggagcttagaggaagatctgagctccaatggctgtga
ccctggtttgccactcatagaagcgacaggaaacatcacttttgccagacggagcgggtggaggccttgtcctgggccgg
>IBE-C5370_fodiens_B
ccggggcggcgccgccacccttgcaggcctcaaggcaatctcgctccttccagggacgctgcaccttccctggagcttagaggaagatctgagctccaatggctgtga
ccctggtttgccactcatagaagcgacaggaaacatcacttttgccagacggagcgggtggaggccttgtcctgggccgg
>IBE-C5603_niethammeri_A
ccggggcggcgccgccacccttgcaggcctcaaggcaatctcgctccttccagggacgctgcaccttccctggagcttagaggaagatctgagctccaatggctgtga
ccctggtttgccactcatagaagcgacaggaaacatcacttttgccagacggagcgggtggaggccttgtcctgggccgg
>IBE-C5603_niethammeri_B
ccggggcggcgccgccacccttgcaggcctcaaggcaatctcgctccttccagggacgctgcaccttccctggagcttagaggaagatctgagctccaatggctgtga
ccctggtttgccactcatagaagcgacaggaaacatcacttttgccagacggagcgggtggaggccttgtcctgggccgg
>IBE-C5604_niethammeri_A
ccggggcggcgccgccacccttgcaggcctcaaggcaatctcgctccttccagggacgctgcaccttccctggagcttagaggaagatctgagctccaatggctgtga
ccctgctttgccactcatagaagcgacaggaaacatcacttttgccagacggagcgggtggaggccttgtcctgggccgg
>IBE-C5604_niethammeri_B
ccggggcggcgccgccacccttgcaggcctcaaggcaatctcgctccttccagggacgctgcaccttccctggagcttagaggaagatctgagctccaatggctgtga
ccctgctttgccactcatagaagcgacaggaaacatcacttttgccagacggagcgggtggaggccttgtcctgggccgg
>IBE-C5383_anomalus_A
ccgggccggcgccgccacccttgcaggcctcaaggcaatctcgctccctccagggacgctgcaccttccctggagcttagaggaacatctgagctccaatggctgtga
ccctggtttaccactcttggaagcgacaggaaacatcacttttgccagagggagcgggtggaggccttgtcctgggccgg
>IBE-C5383_anomalus_B
ccgggccggcgccgccacccttgcaggcctcaaggcaatctcgctccctccagggacgctgcaccttccctggagcttagaggaacatctgagctccaatggctgtga
ccctggtttaccactcttggaagcgacaggaaacatcacttttgccagagggagcgggtggaggccttgtcctgggccgg
>IBE-C5719_anomalus_A
ccgggccggcgccgccacccttgcaggcctcaaggcaatctcgctccctccagggacgctgcaccttccctggagcttagaggaacatctgagctccaatggctgtga
ccctggtttaccactcttggaagcgacaggaaacatcacttttgccagagggagcgggtggaggccttgtcctgggccgg
>IBE-C5719_anomalus_B
ccgggccggcgccgccacccttgcaggcctcaaggcaatctcgctccctccagggacgctgcaccttccctggagcttagaggaacatctgagctccaatggctgtga
ccctggtttaccactcttggaagcgacaggaaacatcacttttgccagagggagcgggtggaggccttgtcctgggccgg
>IBE-C5720_anomalus_A
ccgggccggcgccgccacccttgcaggcctcaaggcaatctcgctccctccagggacgctgcaccttccctggagcttagaggaacatctgagctccaatggctgtga
ccctggtttaccactcttggaagcgacaggaaacatcacttttgccagagggagcgggtggaggccttgtcctgggccgg
>IBE-C5720_anomalus_B
ccgggccggcgccgccacccttgcaggcctcaaggcaatctcgctccctccagggacgctgcaccttccctggagcttagaggaacatctgagctccaatggctgtga
ccctggtttaccactcttggaagcgacaggaaacatcacttttgccagagggagcgggtggaggccttgtcctgggccgg
>IBE-C5699_niethammeri_A
ccggggcggcgccgccacccttgcaggcctcaaggcaatctcgctccttccagggacgctgcaccttccctggagcttagaggaagatctgagctccaatggctgtga
ccctggtttgccactcatagaagcgacaggaaacatcacttttgccagacggagcgggtggaggccttgtcctgggccgg
>IBE-C5699_niethammeri_B
ccggggcggcgccgccacccttgcaggcctcaaggcaatctcgctccttccagggacgctgcaccttccctggagcttagaggaagatctgagctccaatggctgtga
ccctgctttgccactcatagaagcgacaggaaacatcacttttgccagacggagcgggtggaggccttgtcctgggccgg
>UAM-24769_fodiens_A
ccggggcggcgccgccacccttgcaggcctcaaggcaatctcgctccttccagggacgctgcaccttccctggagcttagaggaagatctgagctccaatggctgtga
ccctggtttgccactcatagaagcgacaggaaacatcacttttgccagacggagcgggtggaggccttgtcctgggccgg
```

>UAM-24769_fodiens_B
ccggggcggcgccgccacccttgcaggcctcaaggcaatctcgctccttccagggacgctgcaccttccctggagcttagaggaagatctgagctccaatggctgtga
ccctggtttgccactcatagaagcgacaggaaacatcacttttgccagacggagcgggtggaggccttgtcctgggccgg
>IBE-C5372_niethammeri_A
ccggggcggcgccgccacccttgcaggcctcaaggcaatctcgctccttccagggacgctgcaccttccctggagcttagaggaagatctgagctccaatggctgtga
ccctggtttgccactcatagaagcgacaggaaacatcacttttgccagacggagcgggtggaggccttgtcctgggccgg
>IBE-C5372_niethammeri_B
ccggggcggcgccgccacccttgcaggcctcaaggcaatctcgctccttccagggacgctgcaccttccctggagcttagaggaagatctgagctccaatggctgtga
ccctggtttgccactcatagaagcgacaggaaacatcacttttgccagacggagcgggtggaggccttgtcctgggccgg
>IBE-C5374_fodiens_A
ccggggcggcgccgccacccttgcaggcctcaaggcaatctcgctccttccagggacgctgcaccttccctggagcttagaggaagatctgagctccaatggctgtga
ccctggtttgccactcatagaagcgacaggaaacatcacttttgccagacggagcgggtggaggccttgtcctgggccgg
>IBE-C5374_fodiens_B
ccggggcggcgccgccacccttgcaggcctcaaggcaatctcgctccttccagggacgctgcaccttccctggagcttagaggaagatctgagctccaatggctgtga
ccctggtttgccactcatagaagcgacaggaaacatcacttttgccagacggagcgggtggaggccttgtcctgggccgg
>IBE-C5726_anomalus_A
ccgggccggcgccgccacccttgcaggcctcaaggcaatctcgctccctccagggacgctgcaccttccctggagcttagaggaacatctgagctccaatggctgtga
ccctggtttaccactcttggaagcgacaggaaacatcacttttgccagagggagcgggtggaggccttgtcctgggccgg
>IBE-C5726_anomalus_B
ccgggccggcgccgccacccttgcaggcctcaaggcaatctcgctccctccagggacgctgcaccttccctggagcttagaggaacatctgagctccaatggctgtga
ccctggtttaccactcttggaagcgacaggaaacatcacttttgccagagggagcgggtggaggccttgtcctgggccgg
>IBE-C5742_fodiens_A
ccggggcggcgccgccacccttgcaggcctcaaggcaatctcgctccttccagggacgctgcaccttccctggagcttagaggaagatctgagctccaatggctgtga
ccctggtttgccactcatagaagcgacaggaaacatcacttttgccagacggagcgggtggaggccttgtcctgggccgg
>IBE-C5742_fodiens_B
ccggggcggcgccgccacccttgcaggcctcaaggcaatctcgctccttccagggacgctgcaccttccctggagcttagaggaagatctgagctccaatggctgtga
ccctgctttgccactcatagaagcgacaggaaacatcacttttgccagacggagcgggtggaggccttgtcctgggccgg
>IBE-C5744_fodiens_A
ccggggcggcgccgccacccttgcaggcctcaaggcaatctcgctccttccagggacgctgcaccttccctggagcttagaggaagatctgagctccaatggctgtga
ccctggtttgccactcatagaagcgacaggaaacatcacttttgccagacggagcgggtggaggccttgtcctgggccgg
>IBE-C5744_fodiens_B
ccggggcggcgccgccacccttgcaggcctcaaggcaatctcgctccttccagggacgctgcaccttccctggagcttagaggaagatctgagctccaatggctgtga
ccctgctttgccactcatagaagcgacaggaaacatcacttttgccagacggagcgggtggaggccttgtcctgggccgg
>IBE-C6063_fodiens_A
ccggggcggcgccgccacccttgcaggcctcaaggcaatctcgctccttccagggacgctgcaccttccctggagcttagaggaagatctgagctccaatggctgtga
ccctggtttgccactcatagaagcgacaggaaacatcacttttgccagacggagcgggtggaggccttgtcctgggccgg
>IBE-C6063_fodiens_B
ccggggcggcgccgccacccttgcaggcctcaaggcaatctcgctccttccagggacgctgcaccttccctggagcttagaggaagatctgagctccaatggctgtga
ccctggtttgccactcatagaagcgacaggaaacatcacttttgccagacggagcgggtggaggccttgtcctgggccgg
>IBE-C6065_anomalus_A
ccgggccggcgccgccacccttgcaggcctcaaggcaatctcgctccctccagggacgctgcaccttccctggagcttagaggaacatctgagctccaatggctgtga
ccctggtttaccactcttggaagcgacaggaaacatcacttttgccagagggagcgggtggaggccttgtcctgggccgg
>IBE-C6065_anomalus_B
ccgggccggcgccgccacccttgcaggcctcaaggcaatctcgctccctccagggacgctgcaccttccctggagcttagaggaacatctgagctccaatggctgtga
ccctggtttaccactcttggaagcgacaggaaacatcacttttgccagagggagcgggtggaggccttgtcctgggccgg
>IBE-C6066_fodiens_A
ccggggcggcgccgccacccttgcaggcctcaaggcaatctcgctccttccagggacgctgcaccttccctggagcttagaggaagatctgagctccaatggctgtga
ccctggtttgccactcatagaagcgacaggaaacatcacttttgccagacggagcgggtggaggccttgtcctgggccgg
>IBE-C6066_fodiens_B
ccggggcggcgccgccacccttgcaggcctcaaggcaatctcgctccttccagggacgctgcaccttccctggagcttagaggaagatctgagctccaatggctgtga
ccctggtttgccactcatagaagcgacaggaaacatcacttttgccagacggagcgggtggaggccttgtcctgggccgg
>IBE-C6069_fodiens_A
ccggggcggcgccgccacccttgcaggcctcaaggcaatctcgctccttccagggacgctgcaccttccctggagcttagaggaagatctgagctccaatggctgtga
ccctggtttgccactcatagaagcgacaggaaacatcacttttgccagacggagcgggtggaggccttgtcctgggccgg
>IBE-C6069_fodiens_B
ccggggcggcgccgccacccttgcaggcctcaaggcaatctcgctccttccagggacgctgcaccttccctggagcttagaggaagatctgagctccaatggctgtga
ccctggtttgccactcatagaagcgacaggaaacatcacttttgccagacggagcgggtggaggccttgtcctgggccgg
>IBE-C4117_fodiens_A
ccggggcggcgccgccacccttgcaggcctcaaggcaatctcgctccttccagggacgctgcaccttccctggagcttagaggaagatctgagctccaatggctgtga
ccctgctttgccactcatagaagcgacaggaaacatcacttttgccagacggagcgggtggaggccttgtcctgggccgg
>IBE-C4117_fodiens_B
ccggggcggcgccgccacccttgcaggcctcaaggcaatctcgctccttccagggacgctgcaccttccctggagcttagaggaagatctgagctccaatggctgtga
ccctgctttgccactcatagaagcgacaggaaacatcacttttgccagacggagcgggtggaggccttgtcctgggccgg
>IBE-C6105_niethammeri_A
ccggggcggcgccgccacccttgcaggcctcaaggcaatctcgctccttccagggacgctgcaccttccctggagcttagaggaagatctgagctccaatggctgtga
ccctggtttgccactcatagaagcgacaggaaacatcacttttgccagacggagcgggtggaggccttgtcctgggccgg
>IBE-C6105_niethammeri_B
ccggggcggcgccgccacccttgcaggcctcaaggcaatctcgctccttccagggacgctgcaccttccctggagcttagaggaagatctgagctccaatggctgtga
ccctggtttgccactcatagaagcgacaggaaacatcacttttgccagacggagcgggtggaggccttgtcctgggccgg
>IBE-C6106_niethammeri_A
ccggggcggcgccgccacccttgcaggcctcaaggcaatctcgctccttccagggacgctgcaccttccctggagcttagaggaagatctgagctccaatggctgtga
ccctggtttgccactcatagaagcgacaggaaacatcacttttgccagacggagcgggtggaggccttgtcctgggccgg
>IBE-C6106_niethammeri_B

ccggggcggcgccgccacccttgcaggcctcaaggcaatctcgctccttccagggacgctgcaccttccctggagcttagaggaagatctgagctccaatggctgtga
ccctggtttgccactcatagaagcgacaggaaacatcacttttgccagacggagcgggtggaggccttgtcctgggccgg
>IBE-C6108_niethammeri_A
ccggggcggcgccgccacccttgcaggcctcaaggcaatctcgctccttccagggacgctgcaccttccctggagcttagaggaagatctgagctccaatggctgtga
ccctgctttgccactcatagaagcgacaggaaacatcacttttgccagacggagcgggtggaggccttgtcctgggccgg
>IBE-C6108_niethammeri_B
ccggggcggcgccgccacccttgcaggcctcaaggcaatctcgctccttccagggacgctgcaccttccctggagcttagaggaagatctgagctccaatggctgtga
ccctgctttgccactcatagaagcgacaggaaacatcacttttgccagacggagcgggtggaggccttgtcctgggccgg
>IBE-C6118_anomalus_A
ccgggccggcgccgccacccttgcaggcctcaaggcaatctcgctccctccagggacgctgcaccttccctggagcttagaggaacatctgagctccaatggctgtga
ccctggtttaccactcttggaagcgacaggaaacatcacttttgccagagggagcgggtggaggccttgtcctgggccgg
>IBE-C6118_anomalus_B
ccgggccggcgccgccacccttgcaggcctcaaggcaatctcgctccctccagggacgctgcaccttccctggagcttagaggaacatctgagctccaatggctgtga
ccctggtttaccactcttggaagcgacaggaaacatcacttttgccagagggagcgggtggaggccttgtcctgggccgg
>IBE-C6121_anomalus_A
ccgggccggcgccgccacccttgcaggcctcaaggcaatctcgctccctccagggacgctgcaccttccctggagcttagaggaacatctgagctccaatggctgtga
ccctggtttaccactcttggaagcgacaggaaacatcacttttgccagagggagcgggtggaggccttgtcctgggccgg
>IBE-C6121_anomalus_B
ccgggccggcgccgccacccttgcaggcctcaaggcaatctcgctccctccagggacgctgcaccttccctggagcttagaggaacatctgagctccaatggctgtga
ccctggtttaccactcttggaagcgacaggaaacatcacttttgccagagggagcgggtggaggccttgtcctgggccgg
>IBE-C6116_niethammeri_A
ccggggcggcgccgccacccttgcaggcctcaaggcaatctcgctccttccagggacgctgcaccttccctggagcttagaggaagatctgagctccaatggctgtga
ccctggtttgccactcatagaagcgacaggaaacatcacttttgccagacggagcgggtggaggccttgtcctgggccgg
>IBE-C6116_niethammeri_B
ccggggcggcgccgccacccttgcaggcctcaaggcaatctcgctccttccagggacgctgcaccttccctggagcttagaggaagatctgagctccaatggctgtga
ccctgctttgccactcatagaagcgacaggaaacatcacttttgccagacggagcgggtggaggccttgtcctgggccgg
>IBE-C6124_anomalus_A
ccgggccggcgccgccacccttgcaggcctcaaggcaatctcgctccctccagggacgctgcaccttccctggagcttagaggaacatctgagctccaatggctgtga
ccctggtttaccactcttggaagcgacaggaaacatcacttttgccagagggagcgggtggaggccttgtcctgggccgg
>IBE-C6124_anomalus_B
ccgggccggcgccgccacccttgcaggcctcaaggcaatctcgctccctccagggacgctgcaccttccctggagcttagaggaacatctgagctccaatggctgtga
ccctggtttaccactcttggaagcgacaggaaacatcacttttgccagagggagcgggtggaggccttgtcctgggccgg
>IBE-C5188_fodiens_A
ccggggcggcgccgccacccttgcaggcctcaaggcaatctcgctccttccagggacgctgcaccttccctggagcttagaggaagatctgagctccaatggctgtga
ccctggtttgccactcatagaagcgacaggaaacatcacttttgccagacggagcgggtggaggccttgtcctgggccgg
>IBE-C5188_fodiens_B
ccggggcggcgccgccacccttgcaggcctcaaggcaatctcgctccttccagggacgctgcaccttccctggagcttagaggaagatctgagctccaatggctgtga
ccctgctttgccactcatagaagcgacaggaaacatcacttttgccagacggagcgggtggaggccttgtcctgggccgg
>IBE-C5359_niethammeri_A
ccggggcggcgccgccacccttgcaggcctcaaggcaatctcgctccttccagggacgctgcaccttccctggagcttagaggaagatctgagctccaatggctgtga
ccctggtttgccactcatagaagcgacaggaaacatcacttttgccagacggagcgggtggaggccttgtcctgggccgg
>IBE-C5359_niethammeri_B
ccggggcggcgccgccacccttgcaggcctcaaggcaatctcgctccttccagggacgctgcaccttccctggagcttagaggaagatctgagctccaatggctgtga
ccctgctttgccactcatagaagcgacaggaaacatcacttttgccagacggagcgggtggaggccttgtcctgggccgg
>IBE-C5371_niethammeri_A
ccggggcggcgccgccacccttgcaggcctcaaggcaatctcgctccttccagggacgctgcaccttccctggagcttagaggaagatctgagctccaatggctgtga
ccctggtttgccactcatagaagcgacaggaaacatcacttttgccagacggagcgggtggaggccttgtcctgggccgg
>IBE-C5371_niethammeri_B
ccggggcggcgccgccacccttgcaggcctcaaggcaatctcgctccttccagggacgctgcaccttccctggagcttagaggaagatctgagctccaatggctgtga
ccctggtttgccactcatagaagcgacaggaaacatcacttttgccagacggagcgggtggaggccttgtcctgggccgg
>MSB-158495_fodiens_A
ccggggcggcgccgccacccttgcaggcctcaaggcaatctcgctccttccagggacgctgcaccttccctggagcttagaggaagatctgagctccaatggctgtga
ccctggtttgccactcatagaagcgacaggaaacatcacttttgccagacggagcgggtggaggccttgtcctgggccgg
>MSB-158495_fodiens_B
ccggggcggcgccgccacccttgcaggcctcaaggcaatctcgctccttccagggacgctgcaccttccctggagcttagaggaagatctgagctccaatggctgtga
ccctggtttgccactcatagaagcgacaggaaacatcacttttgccagacggagcgggtggaggccttgtcctgggccgg
>MSB-158621_fodiens_A
ccggggcggcgccgccacccttgcaggcctcaaggcaatctcgctccttccagggacgctgcaccttccctggagcttagaggaagatctgagctccaatggctgtga
ccctggtttgccactcatagaagcgacaggaaacatcacttttgccagacggagcgggtggaggccttgtcctgggccgg
>MSB-158621_fodiens_B
ccggggcggcgccgccacccttgcaggcctcaaggcaatctcgctccttccagggacgctgcaccttccctggagcttagaggaagatctgagctccaatggctgtga
ccctggtttgccactcatagaagcgacaggaaacatcacttttgccagacggagcgggtggaggccttgtcctgggccgg
>MSB-288577_fodiens_A
ccggggcggcgccgccacccttgcaggcctcaaggcaatctcgctccttccagggacgctgcaccttccctggagcttagaggaagatctgagctccaatggctgtga
ccctggtttgccactcatagaagcgacaggaaacatcacttttgccagacggagcgggtggaggccttgtcctgggccgg
>MSB-288577_fodiens_B
ccggggcggcgccgccacccttgcaggcctcaaggcaatctcgctccttccagggacgctgcaccttccctggagcttagaggaagatctgagctccaatggctgtga
ccctggtttgccactcatagaagcgacaggaaacatcacttttgccagacggagcgggtggaggccttgtcctgggccgg
>MSB-95472_fodiens_A
ccggggcggcgccgccacccttgcaggcctcaaggcaatctcgctccttccagggacgctgcaccttccctggagcttagaggaagatctgagctccaatggctgtga
ccctgctttgccactcatagaagcgacaggaaacatcacttttgccagacggagcgggtggaggccttgtcctgggccgg
>MSB-95472_fodiens_B
ccggggcggcgccgccacccttgcaggcctcaaggcaatctcgctccttccagggacgctgcaccttccctggagcttagaggaagatctgagctccaatggctgtga
ccctgctttgccactcatagaagcgacaggaaacatcacttttgccagacggagcgggtggaggccttgtcctgggccgg

>MSB-293526_fodiens_A
ccggggcggcgccgccacccttgcaggcctcaaggcaatctcgctccttccagggacgctgcaccttccctggagcttagaggaagatctgagctccaatggctgtga
ccctggtttgccactcatagaagcgacaggaaacatcacttttgccagacggagcgggtggaggccttgtcctgggccgg
>MSB-293526_fodiens_B
ccggggcggcgccgccacccttgcaggcctcaaggcaatctcgctccttccagggacgctgcaccttccctggagcttagaggaagatctgagctccaatggctgtga
ccctggtttgccactcatagaagcgacaggaaacatcacttttgccagacggagcgggtggaggccttgtcctgggccgg
>MSB-288698_fodiens_A
ccggggcggcgccgccacccttgcaggcctcaaggcaatctcgctccttccagggacgctgcaccttccctggagcttagaggaagatctgagctccaatggctgtga
ccctggtttgccactcatagaagcgacaggaaacatcacttttgccagacggagcgggtggaggccttgtcctgggccgg
>MSB-288698_fodiens_B
ccggggcggcgccgccacccttgcaggcctcaaggcaatctcgctccttccagggacgctgcaccttccctggagcttagaggaagatctgagctccaatggctgtga
ccctggtttgccactcatagaagcgacaggaaacatcacttttgccagacggagcgggtggaggccttgtcctgggccgg
>IBE-C4515_anomalus_A
ccgggccggcgccgccacccttgcaggcctcaaggcaatctcgctccctccagggacgctgcaccttccctggagcttagaggaacatctgagctccaatggctgtga
ccctggtttaccactcttggaagcgacaggaaacatcacttttgccagagggagcgggtggaggccttgtcctgggccgg
>IBE-C4515_anomalus_B
ccgggccggcgccgccacccttgcaggcctcaaggcaatctcgctccctccagggacgctgcaccttccctggagcttagaggaacatctgagctccaatggctgtga
ccctggtttaccactcttggaagcgacaggaaacatcacttttgccagagggagcgggtggaggccttgtcctgggccgg

JMJD-intron-2-oneseq.fasta
>IBE-C1529_anomalus_A
aggtgcctgagccttttcaggagctaggggggcatcacgataacctagggcagccgcgtgaccctcgagtgtctctcgcgtgcccctcacgactcctgggcatctgac
tttactcccatccacagctcccctcctagtccgcccagtgc
>IBE-C1529_anomalus_B
aggtgcctgagccttttcaggagctaggggggcatcacgataacctagggcagccgtgtgaccctcgagtgtctctcgcgtgcccctcacgactcctgggcatctgac
tttactcccatccacagctcccctcctagtccgcccagtgc
>IBE-C1789_anomalus_A
aggtgcctgagccttttcaggagctaggggggcatcacgataacctagggcagccgcgtgaccctcgagtgtctctcgcgtgcccctcacgactcctgggcatctgac
tttactcccatccacagctcccctcctagtccgcccagtgc
>IBE-C1789_anomalus_B
aggtgcctgagccttttcaggagctaggggggcatcacgataaccttgggcagccgcgtgaccctcgagtgtctctcgcgtgcccctcacgactcctgggcatctgac
tttactcccatccacagctcccctcctagtccgcccagtgc
>IBE-C1808_milleri_A
aggtgcctgagccttttcaggagctaggggggcatcacgataacccagggcagccgcgtgaccctcgagtgtctcttgcgtgcccctcccgactcctgggcatctgac
tttactcccatccccagctcccctcctagtccgcccagtgc
>IBE-C1808_milleri_B
aggtgcctgagccttttcaggagctaggggggcatcacgataacccagggcagccgcgtgaccctcgagtgtctcttgcgtgcccctcccgactcctgggcatctgac
tttactcccatccccagctcccctcctagtccgcccagtgc
>IBE-C2895_anomalus_A
aggtgcctgagccttttcaggagctaggggggcatcacgataacctagggcagccgcgtgaccctcgagtgtctctcgcgtgcccctcacgactcctgggcatctgac
tttactcccatccacagctcccctcctagtccgcccagtgc
>IBE-C2895_anomalus_B
aggtgcctgagccttttcaggagctaggggggcatcacgataaccttgggcagccgcgtgaccctcgagtgtctctcgcgtgcccctcacgactcctgggcatctgac
tttactcccatccacagctcccctcctagtccgcccagtgc
>IBE-C3786_milleri_A
aggtgcctgagccttttcaggagctaggggggcatcacgataacccagggcagccgcgtgaccctcgagtgtctcttgcgtgcccctcccgactcctgggcatctgac
tttactcccatccccagctcctctcctagtccgcccagtgc
>IBE-C3786_milleri_B
aggtgcctgagccttttcaggagctaggggggcatcacgataacccagggcagccgcgtgaccctcgagtgtctcttgcgtgcccctcccgactcctgggcatctgac
tttactcccatccccagctcctctcctagtccgcccagtgc
>IBE-C4116_milleri_A
aggtgcctgagccttttcaggagctaggggggcatcacgataacccagggcagccgcgtgaccctcgagtgtctcttgcgtgcccctcacgactcctgggcatctgac
tttactcccatccccagctcccctcctagtccgcccagtgc
>IBE-C4116_milleri_B
aggtgcctgagccttttcaggagctaggggggcatcacgataacctagggcagccgcgtgaccctcgagtgtctctcgcgtgcccctcacgactcctgggcatctgac
tttactcccatccccagctcccctcctagtccacccagtgc
>IBE-C101_fodiens_A
aggtgcctgagccttttcagaagctaggggggcatcacgataacccaggtcagccgagtgaccctcgagtgtctctcgcatgcccctcacgactcctgggcatcagac
ttgactcccatccccagttctcctcctagtccacccagtgc
>IBE-C101_fodiens_B
aggtgcctgagccttttcagaagctaggggggcatcacgataacccaggtcagccgagtgaccctcgagtgtctctcgcatgcccctcacgactcctgggcatcagac
ttgactcccatccccagttctcctcctagtccacccagtgc
>IBE-C1914_fodiens_A
aggtgcctgagccttttcagaagctaggggggcatcacgataacccaggtcagccgagtgaccctcgagtgtctctcgcatgcccctcacgactcctgggcatcagac
tttactcccatccccagttctcctcctagtccacccagtgc
>IBE-C1914_fodiens_B
aggtgcctgagccttttcagaagctaggggggcatcacgataacccaggtcagccgagtgaccctcgagtgtctctcgcatgcccctcacgactcctgggcatcagac
tttactcccatccccagttctcctcctagtccacccagtgc
>IBE-C4120_teres_A
aggtgcctgagccttttcaggagctaggggggcatcacgataacccagggcagccgcgtgaccctcgagtgtctctcgcgtgcccctcacaactcctgggcatctgac
tttactcccatccacagctcccctcctagtccgcccagtgc
>IBE-C4120_teres_B
aggtgcctgagccttttcaggagctaggggggcatcacgataacccagggcagccgcgtgaccctcgagtgtctctcgcgtgcccctcacaactcctgggcatctgac
tttactcccatccacagctcccctcctagtccgcccagtgc
>IBE-C4122_teres_A
aggtgcctgagccttttcaggagctaggggggcatcacgataacccagggcagccgcgtgaccctcgagtgtctctcgcgtgcccctcacaactcctgggcatctgac
tttactcccatccacagctcccctcctagtccgcccagtgc
>IBE-C4122_teres_B
aggtgcctgagccttttcaggagctaggggggcatcacgataacccagggcagccgcgtgaccctcgagtgtctctcgcgtgcccctcacgactcctgggcatctgac
tttactcccatccccagctcccctcctagtccgcccagtgc
>IBE-C1927_milleri_A
aggtgcctgagccttttcaggagctaggggggcatcacgataacccagggcagccgcgtgaccctcgagtgtctcttgcgtgcccctcccgactcctgggcatctgac
tttactcccatccccagctcccctcctagtccgcccagtgc
>IBE-C1927_milleri_B
aggtgcctgagccttttcaggagctaggggggcatcacgataacccagggcagccgcgtgaccctcgagtgtctcttgcgtgcccctcccgactcctgggcatctgac
tttactcccatccccagctcccctcctagtccgcccagtgc
>IBE-C5182_niethammeri_A
aggtgcctgagccttttcagaagctaggggggcatcacgataacccaggtcagccgagtgaccctcgagtgtctctcgcatgcccctcacgactcctgggcatcagac
ttgactcccatccccagttctcctcctagtccacccagtgc
>IBE-C5182_niethammeri_B
aggtgcctgagccttttcagaagctaggggggcatcacgataacccaggtcagccgagtgaccctcgagtgtctctcgcatgcccctcacgactcctgggcatcagac
tttactcccatccccagttctcctcctagtccacccagtgc
>IBE-C5367_fodiens_A

aggtgcctgagccttttcagaagctaggggggcatcacgataacccaggtcagccgagtgaccctcgagtgtctctcgcatgcccctcacgactcctgggcatcagac
tttactcccatccccagttctcctcctagtccacccagtgc
>IBE-C5367_fodiens_B
aggtgcctgagccttttcagaagctaggggggcatcacgataacccaggtcagccgagtgaccctcgagtgtctctcgcatgcccctcacgactcctgggcatcagac
tttactcccatccccagttctcctcctagtccacccagtgc
>IBE-C5368_niethammeri_A
aggtgcctgagccttttcagaagctaggggggcatcacgataacccaggtcagccgagtgaccctcgagtgtctctcgcatgcccctcacgactcctgggcatcagac
ttgactcccatccccagttctcctcctagtccacccagtgc
>IBE-C5368_niethammeri_B
aggtgcctgagccttttcagaagctaggggggcatcacgataacccaggtcagccgagtgaccctcgagtgtctctcgcatgcccctcacgactcctgggcatcagac
tttactcccatccccagttctcctcctagtccacccagtgc
>IBE-C5697_niethammeri_A
aggtgcctgagccttttcagaagctaggggggcatcacgataacccaggtcagccgagtgaccctcgagtgtctctcgcatgcccctcacgactcctgggcatcagac
tttactcccatccccagttctcctcctagtccacccagtgc
>IBE-C5697_niethammeri_B
aggtgcctgagccttttcagaagctaggggggcatcacgataacccaggtcagccgagtgaccctcgagtgtctctcgcatgcccctcacgactcctgggcatcagac
tttactcccatccccagttctcctcctagtccacccagtgc
>IBE-C5700_anomalus_A
aggtgcctgagccttttcaggagctaggggggcatcacgataacctagggcagccgcgtgaccctcgagtgtctctcgcgtgcccctcacgactcctgggcatctgac
tttactcccatccacagctcccctcctagtccgcccagtgc
>IBE-C5700_anomalus_B
aggtgcctgagccttttcaggagctaggggggcatcacgataacctagggcagccgcgtgaccctcgagtgtctctcgcgtgcccctcacgactcctgggcatctgac
tttactcccatccacagctcccctcctagtccgcccagtgc
>IBE-C5701_niethammeri_A
aggtgcctaagccttttcagaagctaggggggcatcacgataacccaggtcagccgagtgaccctcgagtgtctctcgcatgcccctcacgactcctgggcatcagac
tttactcccatccccagttctcctcctagtccacccagtgc
>IBE-C5701_niethammeri_B
aggtgcctaagccttttcagaagctaggggggcatcacgataacccaggtcagccgagtgaccctcgagtgtctctcgcatgcccctcacgactcctgggcatcagac
tttactcccatccccagttctcctcctagtccacccagtgc
>IBE-C5603_niethammeri_A
aggtgcctgagccttttcagaagctaggggggcatcacgataacccaggtcagccgagtgaccctcgagtgtctctcgcatgcccctcacgactcctgggcatcagac
tttactcccatccccagttctcctcctagtccacccagtgc
>IBE-C5603_niethammeri_B
aggtgcctgagccttttcagaagctaggggggcatcacgataacccaggtcagccgagtgaccctcgagtgtctctcgcatgcccctcacgactcctgggcatcagac
tttactcccatccccagttctcctcctagtccacccagtgc
>IBE-C5604_niethammeri_A
aggtgcctgagccttttcagaagctaggggggcatcacgataacccaggtcagccgagtgaccctcgagtgtctctcgcatgcccctcacgactcctgggcatcagac
tttactcccatccccagttctcctcctagtccacccagtgc
>IBE-C5604_niethammeri_B
aggtgcctgagccttttcagaagctaggggggcatcacgataacccaggtcagccgagtgaccctcgagtgtctctcgcatgcccctcacgactcctgggcatcagac
tttactcccatccccagttctcctcctagtccacccagtgc
>IBE-C5370_fodiens_A
aggtgcctgagccttttcagaagctaggggggcatcacgataacccaggtcagccgagtgaccctcgagtgtctctcgcatgcccctcacgactcctgggcatcagac
tttactcccatccccagttctcctcctagtccacccagtgc
>IBE-C5370_fodiens_B
aggtgcctgagccttttcagaagctaggggggcatcacgataacccaggtcagccgagtgaccctcgagtgtctctcgcatgcccctcacgactcctgggcatcagac
tttactcccatccccagttctcctcctagtccacccagtgc
>IBE-C5383_anomalus_A
aggtgcctgagccttttcaggagctaggggggcatcacgataacctagggcagccgcgtgaccctcgagtgtctctcgcgtgcccctcacgactcctgggcatctgac
tttactcccatccacagctcccctcctagtccgcccagtgc
>IBE-C5383_anomalus_B
aggtgcctgagccttttcaggagctaggggggcatcacgataacctagggcagccgcgtgaccctcgagtgtctctcgcgtgcccctcacgactcctgggcatctgac
tttactcccatccacagctcccctcctagtccgcccagtgc
>IBE-C5719_anomalus_A
aggtgcctgagccttttcaggagctaggggggcatcacgataacctagggcagccgcgtgaccctcgagtgtctctcgcgtgcccctcacgactcctgggcatctgac
tttactcccatccacagctcccctcctagtccgcccagtgc
>IBE-C5719_anomalus_B
aggtgcctgagccttttcaggagctaggggggcatcacgataacctagggcagccgcgtgaccctcgagtgtctctcgcgtgcccctcacgactcctgggcatctgac
tttactcccatccacagctcccctcctagtccgcccagtgc
>IBE-C5720_anomalus_A
aggtgcctgagccttttcaggagctaggggggcatcacgataacctagggcagccgcgtgaccctcgagtgtctctcgcgtgcccctcacgactcctgggcatctgac
tttactcccatccacagctcccctcctagtccgcccagtgc
>IBE-C5720_anomalus_B
aggtgcctgagccttttcaggagctaggggggcatcacgataaccttgggcagccgcgtgaccctcgagtgtctctcgcgtgcccctcacgactcctgggcatctgac
tttactcccatccacagctcccctcctagtccgcccagtgc
>UAM-24769_fodiens_A
aggtgcctgagccttttcagaagctaggggggcatcacgataacccaggtcagccgagtgaccctcgagtgtctctcgcatgcccctcacgactcctaggcatcagac
tttactcccatccccagttctcctcctagtccacccagtgc
>UAM-24769_fodiens_B
aggtgcctgagccttttcagaagctaggggggcatcacgataacccaggtcagccgagtgaccctcgagtgtctctcgcatgcccctcacgactcctaggcatcagac
tttactcccatccccagttctcctcctagtccacccagtgc
>UAM-24770_fodiens_A
aggtgcctgagccttttcagaagctaggggggcatcacgataacccaggtcagccgagtgaccctcgagtgtctctcgcatgcccctcacgactcctaggcatcagac
tttactcccatccccagttctcctcctagtccacccagtgc

>UAM-24770_fodiens_B
aggtgcctgagccttttcagaagctagggggcatcacgataacccaggtcagccgagtgaccctcgagtgtctctcgcatgcccctcacgactcctaggcatcagac
tttactcccatccccagttctcctcctagtccacccagtgc
>IBE-C5372_niethammeri_A
aggtgcctgagccttttcagaagctagggggcatcacgataacccaggtcagccgagtgaccctcgagtgtctctcgcatgcccctcacgactcctgggcatcagac
tttactcccatccccagttctcctcctagtccacccagtgc
>IBE-C5372_niethammeri_B
aggtgcctgagccttttcagaagctagggggcatcacgataacccaggtcagccgagtgaccctcgagtgtctctcgcatgcccctcacgactcctgggcatcagac
tttactcccatccccagttctcctcctagtccacccagtgc
>IBE-C5374_fodiens_A
aggtgcctgagccttttcagaagctagggggcatcacgataacccaggtcagccgagtgaccctcgagtgtctctcgcatgcccctcacgactcctgggcatcagac
ttgactcccatccccagttctcctcctagtccacccagtgc
>IBE-C5374_fodiens_B
aggtgcctgagccttttcagaagctagggggcatcacgataacccaggtcagccgagtgaccctcgagtgtctctcgcatgcccctcacgactcctgggcatcagac
ttgactcccatccccagttctcctcctagtccacccagtgc
>IBE-C5726_anomalus_A
aggtgcctgagccttttcaggagctagggggcatcacgataacctagggcagccgcgtgaccctcgagtgtctctcgcgtgcccctcacgactcctgggcatctgac
tttactcccatccacagctcccctcctagtccgcccagtgc
>IBE-C5726_anomalus_B
aggtgcctgagccttttcaggagctagggggcatcacgataacctagggcagccgcgtgaccctcgagtgtctctcgcgtgcccctcacgactcctgggcatctgac
tttactcccatccacagctcccctcctagtccgcccagtgc
>IBE-C5742_fodiens_A
aggtgcctgagccttttcagaagctagggggcatcacgataacccaggtcagccgagtgaccctcgagtgtctctcgcatgcccctcacgactcctgggcatcagac
ttgactcccatccccagttctcctcctagtccacccagtgc
>IBE-C5742_fodiens_B
aggtgcctgagccttttcagaagctagggggcatcacgataacccaggtcagccgagtgaccctcgagtgtctctcgcatgcccctcacgactcctgggcatcagac
tttactcccatccccagttctcctcctagtccacccagtgc
>IBE-C5744_fodiens_A
aggtgcctgagccttttcagaagctagggggcatcacgataacccaggtcagccgagtgaccctcgagtgtctctcgcatgcccctcacgactcctgggcatcagac
tttactcccatccccagttctcctcctagtccacccagtgc
>IBE-C5744_fodiens_B
aggtgcctgagccttttcagaagctagggggcatcacgataacccaggtcagccaagtgaccctcgagtgtctctcgcatgcccctcacgactcctgggcatcagac
tttactcccatccccagttctcctcctagtccacccagtgc
>IBE-C6063_fodiens_A
aggtgcctgagccttttcagaagctagggggcatcacgataacccaggtcagccgagtgaccctcgagtgtctctcgcatgcccctcacgactcctgggcatcagac
ttgactcccatccccagttctcctcctagtccacccagtgc
>IBE-C6063_fodiens_B
aggtgcctgagccttttcagaagctagggggcatcacgataacccaggtcagccgagtgaccctcgagtgtctctcgcatgcccctcacgactcctgggcatcagac
tttactcccatccccagttctcctcctagtccacccagtgc
>IBE-C6065_anomalus_A
aggtgcctgagccttttcaggagctagggggcatcacgataacctagggcagccgcgtgaccctcgagtgtctctcgcgtgcccctcacgactcctgggcatctgac
tttactcccatccacagctcccctcctagtccgcccagtgc
>IBE-C6065_anomalus_B
aggtgcctgagccttttcaggagctagggggcatcacgataacctagggcagccgcgtgaccctcgagtgtctctcgcgtgcccctcacgactcctgggcatctgac
tttactcccatccacagctcccctcctagtccgcccagtgc
>IBE-C6066_fodiens_A
aggtgcctgagccttttcagaagctagggggcatcacgataacccaggtcagccgagtgaccctcgagtgtctctcgcatgcccctcacgactcctgggcatcagac
tttactcccatccccagttctcctcctagtccacccagtgc
>IBE-C6066_fodiens_B
aggtgcctgagccttttcagaagctagggggcatcacgataacccaggtcagccaagtgaccctcgagtgtctctcgcatgcccctcacgactcctgggcatcagac
tttactcccatccccagttctcctcctagtccacccagtgc
>IBE-C6069_fodiens_A
aggtgcctgagccttttcagaagctagggggcatcacgataacccaggtcagccgagtgaccctcgagtgtctctcgcatgcccctcacgactcctgggcatcagac
ttgactcccatccccagttctcctcctagtccacccagtgc
>IBE-C6069_fodiens_B
aggtgcctgagccttttcagaagctagggggcatcacgataacccaggtcagccgagtgaccctcgagtgtctctcgcatgcccctcacgactcctgggcatcagac
tttactcccatccccagttctcctcctagtccacccagtgc
>IBE-C4117_fodiens_A
aggtgcctgagccttttcagaagctagggggcatcacgataacccaggtcagccgagtgaccctcgagtgtctctcgcatgcccctcacgactcctaggcatcagac
tttactcccatccccagttctcctcctagtccacccagtgc
>IBE-C4117_fodiens_B
aggtgcctgagccttttcagaagctagggggcatcacgataacccaggtcagccgagtgaccctcgagtgtctctcgcatgcccctcacgactcctaggcatcagac
tttactcccatccccagttctcctcctagtccacccagtgc
>IBE-C6106_niethammeri_A
aggtgcctgagccttttcagaagctagggggcatcacgataacccaggtcagccgagtgaccctcgagtgtctctcgcatgcccctcacgactcctgggcatcagac
tttactcccatccccagttctcctcctagtccacccagtgc
>IBE-C6106_niethammeri_B
aggtgcctgagccttttcagaagctagggggcatcacgataacccaggtcagccgagtgaccctcgagtgtctctcgcatgcccctcacgactcctgggcatcagac
tttactcccatccccagttctcctcctagtccacccagtgc
>IBE-C6108_niethammeri_A
aggtgcctgagccttttcagaagctagggggcatcacgataacccaggtcagccgagtgaccctcgagtgtctctcgcatgcccctcacgactcctgggcatcagac
tttactcccatccccagttctcctcctagtccacccagtgc
>IBE-C6108_niethammeri_B

aggtgcctgagccttttcagaagctagggggcatcacgataacccaggtcagccgagtgaccctcgagtgtctctcgcatgcccctcacgactcctgggcatcagac
tttactcccatccccagttctcctcctagtccacccagtgc
>IBE-C6118_anomalus_A
aggtgcctgagccttttcaggagctagggggcatcacgataacctagggcagccgcgtgaccctcgagtgtctctcgcgtgcccctcacgactcctgggcatctgac
tttactcccatccacagctcccctcctagtccgcccagtgc
>IBE-C6118_anomalus_B
aggtgcctgagccttttcaggagctagggggcatcacgataacctagggcagccgcgtgaccctcgagtgtctctcgcgtgcccctcacgactcctgggcatctgac
tttactcccatccacagctcccctcctagtccgcccagtgc
>IBE-C6121_anomalus_A
aggtgcctgagccttttcaggagctagggggcatcacgataacctagggcagccgcgtgaccctcgagtgtctctcgcgtgcccctcacgactcctgggcatctgac
tttactcccatccacagctcccctcctagtccgcccagtgc
>IBE-C6121_anomalus_B
aggtgcctgagccttttcaggagctagggggcatcacgataacctagggcagccgcgtgaccctcgagtgtctctcgcgtgcccctcacgactcctgggcatctgac
tttactcccatccacagctcccctcctagtccgcccagtgc
>IBE-C6110_niethammeri_A
aggtgcctgagccttttcagaagctagggggcatcacgataacccaggtcagccgagtgaccctcgagtgtctctcgcatgcccctcacgactcctgggcatcagac
ttgactcccatccccagttctcctcctagtccacccagtgc
>IBE-C6110_niethammeri_B
aggtgcctgagccttttcagaagctagggggcatcacgataacccaggtcagccgagtgaccctcgagtgtctctcgcatgcccctcacgactcctgggcatcagac
tttactcccatccccagttctcctcctagtccacccagtgc
>IBE-C6116_niethammeri_A
aggtgcctgagccttttcagaagctagggggcatcacgataacccaggtcagccgagtgaccctcgagtgtctctcgcatgcccctcacgactcctgggcatcagac
ttgactcccatccccagttctcctcctagtccacccagtgc
>IBE-C6116_niethammeri_B
aggtgcctgagccttttcagaagctagggggcatcacgataacccaggtcagccgagtgaccctcgagtgtctctcgcatgcccctcacgactcctgggcatcagac
tttactcccatccccagttctcctcctagtccacccagtgc
>IBE-C6125_anomalus_A
aggtgcctgagccttttcaggagctagggggcatcacgataacctagggcagccgcgtgaccctcgagtgtctctcgcgtgcccctcacgactcctgggcatctgac
tttactcccatccacagctcccctcctagtccgcccagtgc
>IBE-C6125_anomalus_B
aggtgcctgagccttttcaggagctagggggcatcacgataacctagggcagccgcgtgaccctcgagtgtctctcgcgtgcccctcacgactcctgggcatctgac
tttactcccatccacagctcccctcctagtccgcccagtgc
>IBE-C5188_fodiens_A
aggtgcctgagccttttcagaagctagggggcatcacgataacccaggtcagccgagtgaccctcgagtgtctctcgcatgcccctcacgactcctgggcatcagac
tttactcccatccccagttctcctcctagtccacccagtgc
>IBE-C5188_fodiens_B
aggtgcctgagccttttcagaagctagggggcatcacgataacccaggtcagccgagtgaccctcgagtgtctctcgcatgcccctcacgactcctgggcatcagac
tttactcccatccccagttctcctcctagtccacccagtgc
>IBE-C5741_niethammeri_A
aggtgcctgagccttttcagaagctagggggcatcacgataacccaggtcagccgagtgaccctcgagtgtctctcgcatgcccctcacgactcctgggcatcagac
tttactcccatccccagttctcctcctagtccacccagtgc
>IBE-C5741_niethammeri_B
aggtgcctgagccttttcagaagctagggggcatcacgataacccaggtcagccgagtgaccctcgagtgtctctcgcatgcccctcacgactcctgggcatcagac
tttactcccatccccagttctcctcctagtccacccagtgc
>MSB-158495_fodiens_A
aggtgcctgagccttttcagaagctagggggcatcacgataacccaggtcagccgagtgaccctcgagtgtctctcgcatgcccctcacgactcctgggcatcagac
ttgactcccatccccagttctcctcctagtccacccagtgc
>MSB-158495_fodiens_B
aggtgcctgagccttttcagaagctagggggcatcacgataacccaggtcagccgagtgaccctcgagtgtctctcgcatgcccctcacgactcctgggcatcagac
ttgactcccatccccagttctcctcctagtccacccagtgc
>MSB-158621_fodiens_A
aggtgcctgagccttttcagaagctagggggcatcacgataacccaggtcagccgagtgaccctcgagtgtctctcgcatgcccctcacgactcctgggcatcagac
ttgactcccatccccagttctcctcctagtccacccagtgc
>MSB-158621_fodiens_B
aggtgcctgagccttttcagaagctagggggcatcacgataacccaggtcagccgagtgaccctcgagtgtctctcgcatgcccctcacgactcctgggcatcagac
ttgactcccatccccagttctcctcctagtccacccagtgc
>MSB-293526_fodiens_A
aggtgcctgagccttttcagaagctagggggcatcacgataacccaggtcagccgagtgaccctcgagtgtctctcgcatgcccctcacgactcctgggcatcagac
ttgactcccatccccagttctcctcctagtccacccagtgc
>MSB-293526_fodiens_B
aggtgcctgagccttttcagaagctagggggcatcacgataacccaggtcagccgagtgaccctcgagtgtctctcgcatgcccctcacgactcctgggcatcagac
ttgactcccatccccagttctcctcctagtccacccagtgc
>MSB-95472_fodiens_A
aggtgcctgagccttttcagaagctagggggcatcacgataacccaggtcagccgagtgaccctcgagtgtctctcgcatgcccctcacgactcctaggcatcagac
tttactcccatccccagttctcctcctagtccacccagtgc
>MSB-95472_fodiens_B
aggtgcctgagccttttcagaagctagggggcatcacgataacccaggtcagccgagtgaccctcgagtgtctctcgcatgcccctcacgactcctaggcatcagac
tttactcccatccccagttctcctcctagtccacccagtgc
>MSB-288698_fodiens_A
aggtgcctgagccttttcagaagctagggggcatcacgataacccaggtcagccgagtgaccctcgagtgtctctcgcatgcccctcacgactcctaggcatcagac
tttactcccatccccagttctcctcctagtccacccagtgc
>MSB-288698_fodiens_B
aggtgcctgagccttttcagaagctagggggcatcacgataacccaggtcagccgagtgaccctcgagtgtctctcgcatgcccctcacgactcctgggcatcagac
ttgactcccatccccagttctcctcctagtccacccagtgc

>MSB-288577_fodiens_A
aggtgcctgagccttttcagaagctagggggggcatcacgataacccaggtcagccgagtgaccctcgagtgtctctcgcatgcccctcacgactcctgggcatcagac
ttgactcccatccccagttctcctcctagtccacccagtgc
>MSB-288577_fodiens_B
aggtgcctgagccttttcagaagctagggggggcatcacgataacccaggtcagccgagtgaccctcgagtgtctctcgcatgcccctcacgactcctgggcatcagac
ttgactcccatccccagttctcctcctagtccacccagtgc
>IBE-C4515_anomalus_A
aggtgcctgagccttttcaggagctagggggggcatcacgataacctagggcagccgcgtgaccctcgagtgtctctcgcgtgcccctcacgactcctgggcatctgac
tttactcccatccacagctcccctcctagtccgcccagtgc
>IBE-C4515_anomalus_B
aggtgcctgagccttttcaggagctagggggggcatcacgataacctagggcagccgcgtgaccctcgagtgtctctcgcgtgcccctcacgactcctgggcatctgac
tttactcccatccacagctcccctcctagtccgcccagtgc

MYCBPAP-intron-11-oneseq.fasta
>IBE-C1529_anomalus_A
acaccctgccagggtacccagatttcctgtttggaacgatcccagttttagttctcaaagtccgacaggcctgggaaccccagtcccggaaaacccggacacgtgggc
ccgggctcccttaagtgatgccaggcctcagctggcttctgccagttgaaacctggctctggcctctcag
>IBE-C1529_anomalus_B
acaccctgccagggtacccagatttcctgtttggaacgatcccagttttagttctcaaagtccgacaggcctgggaaccccagtcccggaaaacccggacacgtgggc
ccgggctcccttaagtgatgccaggcctcagctggcttctgccagttgaaacctggctctggcctctcag
>IBE-C1789_anomalus_A
acaccctgccagggtacccagatttcctgtttggaacgatcccagttttagttctcaaagtccgacaggcctgggaaccccagtcccggaaaacccggacacgtgggc
ccgggctcccttaagtgatgccaggcctcagctggcttctgccagttgaaacctggctctggcctctcag
>IBE-C1789_anomalus_B
acaccctgccagggtacccagatttcctgtttggaacgatcccagttttagttctcaaagtccgacaggcctgggaaccccagtcccggaaaacccggacacgtgggc
ccgagctcccttaagtgttgccaggcctcagctggcttctgccagttgaaacctggctctggcctctcag
>IBE-C1808_milleri_A
acaccctgccagggtacccagatttcctgtttggaacgatcccagttttagttctcaaagtccgacaggcctgggaaccccagtcccggaaaacccggacacgtgggc
ccgggctcccttaagtgatgccaggcctcagctggcttctgccagttgaaacctggctctggcctctcag
>IBE-C1808_milleri_B
acaccctgccagggtacccagatttcctgtttggaacgatcccagttttagttctcaaagtccgacaggcctgggaaccccagtcccggaaaacccggacacgtgggc
ccgggctcccttaagtgttgccaggcctcagctggcttctgccagttgaaacctggctctggcctctcag
>IBE-C2895_anomalus_A
acaccctgccagggtacccagatttcctgtttggaacgatcccagttttagttctcaaagtccgacaggcctgggaaccccagtcccggaaaacccggacacgtgggc
ccgggctcccttaagtgatgccaggcctcagctggcttctgccagttgaaacctggctctggcctctcag
>IBE-C2895_anomalus_B
acaccctgccagggtacccagatttcctgtttggaacgatcccagttttagttctcaaagtccgacaggcctgggaaccccagtcccggaaaacccggacacgtgggc
ccgggctcccttaagtgatgccaggcctcagctggcttctgccagttgaaacctggctctggcctctcag
>IBE-C3786_milleri_A
acgccctgccagggtacccagatttcctgtttggaacgatcccagttttagttctcaaagtccgacaggcctgggaaccccagtcccggaaaacccggacacgtgggc
ccgggctcccttaagtgttgccaggcctcagctggcttctgccagttgaaacctggctctggcctctcag
>IBE-C3786_milleri_B
acgccctgccagggtacccagatttcctgtttggaacgatcccagttttagttctcaaagtccgacaggcctgggaaccccagtcctggaaaacccggacacgtgggc
ccgggctcccttaagtgttgccaggcctcagctggcttctgccagttgaaacctggctctggcctctcag
>IBE-C4116_milleri_A
acgccctgccagggtacccagatttcctgtttggaacgatcccagttttagttctcaaagtccgacaggcctgggaaccccagtcccggaaaacccggacacgtgggc
ccgggctcccttaagtgttgccaggcctcagctggcttctgccagttgaaacctggctctggcctctcag
>IBE-C4116_milleri_B
acgccctgccagggtacccagatttcctgtttggaacgatcccagttttagttctcaaagtccgacaggcctgggaaccccagtcccggaaaacccggacacgtgggc
ccgggctcccttaagtgttgccaggcctcagctggcttctgccagttgaaacctggctctggcctctcag
>IBE-C101_fodiens_A
acgccctgccagggtacccagatttcctgtttggaacgatcccagttttagttctcaaagtccgacaggcctgggaaccccagtcccggaaaacccggacacgtgggc
ctgggctcccttaagtgttgccaggcctcagctggcttctgccagttgaaccctggctctggcctctcag
>IBE-C101_fodiens_B
acgccctgccagggtacccagatttcctgtttggaacgatcccagttttagttctcaaagtccgacaggcctgggaaccccagtcccggaaaacccggacacgtgggc
ctgggctcccttaagtgttgccaggcctcagctggcttctgccagttgaaccctggctctggcctctcag
>IBE-C1914_fodiens_A
acgccctgccagggtacccagatttcctgtttggaacgatcccagttttagttctcaaagtccgacaggcctgggaaccccagtcccggaaaacccagacacgtgggc
ctgggctcccttaagtgttgccacgcctcagctggcttctgccagttgaaccctggctctggcctctcag
>IBE-C1914_fodiens_B
acgccctgccagggtacccagatttcctgtttggaacgatcccagttttagttctcaaagtccgacaggcctgggaaccccagtcccggaaaacccagacacgtgggc
ctgggctcccttaagtgttgccacgcctcagctggcttctgccagttgaaccctggctctggcctctcag
>IBE-C4120_teres_A
acaccctgccagggtacccagatttcctgtttggaacgatcccagttttagttctcaaagtccgacaggcctgggaaccccagtcccggaaaacccggacacgtgggc
ccgggctcccttaagtgttgccaggcctcagctggctcctgccagttgaaacctggctctggcctctcag
>IBE-C4120_teres_B
acgccctgccagggtacccagatttcctgtttggaatgatcccagttttagttctcaaagtccgacaggcctgggaaccccagtcccggaaaacccggacacgtgggc
ccgggctcccttaagtgttgccaggcctcagctggcttctgccagttgaaacctggctctggcctctcag
>IBE-C4122_teres_A
acgccctgccagggtacccagatttcctgtttggaatgatcccagttttagttctcaaagtccgacaggcctgggaaccccagtcccggaaaacccggacacgtgggc
ccgggctcccttaagtgttgccaggcctcagctggcttctgccagttgaaacctggctctggcctctcag
>IBE-C4122_teres_B
acgccctgccagggtacccagatttcctgtttggaacgatcccagttttagttctcaaagtccgacaggcctgggaaccccagtcccggaaaacccggacacgtgggc
ctgggctcccttaagtgttgccaggcctcagctggctcctgccagttgaaacctggctctggcctctcag
>IBE-C5182_niethammeri_A
acgccctgccagggtacccagatttcctgtttggaacgatcccagttttagttctcaaagtccgacaggcctgggaaccccagtcccggaaaacccggacacgtgggc
ctgggctcccttaagtgttgccaggcctcagctggcttctgccagttgaaccctggctctggcctctcag
>IBE-C5182_niethammeri_B
acgccctgccagggtacccagatttcctgtttggaacgatcccagttttagttctcaaagtccgacaggcctgggaaccccagtcccggaaaacccggacacgtgggc
ctgggctcccttaagtgttgccaggcctcagctggcttctgccagttgaaccctggctctggcctctcag
>IBE-C5367_fodiens_A
acgccctgccagggtacccagatttcctgtttggaacgatcccagttttagttctcaaagtccgacaggcctgggaaccccagtcccggaaaacccagacacgtgggc
ctgggctcccttaagtgttgccacgcctcagctggcttctgccagttgaaccctggctctggcctctcag
>IBE-C5367_fodiens_B
acgccctgccagggtacccagatttcctgtttggaacgatcccagttttagttctcaaagtccgacaggcctgggaaccccagtcccggaaaacccagacacgtgggc
ctgggctcccttaagtgttgccacgcctcagctggcttctgccagttgaaccctggctctggcctctcag
>IBE-C5368_niethammeri_A

acgccctgccagggtacccagatttcctgtttggaacgatcccagttttagttctcaaagtccgacaggcctgggaaccccagtcccggaaaacccggacacgtgggc
ctgggctcccttaagtgttgccaggcctcagctggcttctgccagttgaaccctggctctggcctctcag
>IBE-C5368_niethammeri_B
acgccctgccagggtacccagatttcctgtttggaacgatcccagttttagttctcaaagtccgacaggcctgggaaccccagtcccggaaaacccggacacgtgggc
ctgggctcccttaagtgttgccaggcctcagctggcttctgccagttgaaccctggctctggcctctcag
>IBE-C5383_anomalus_A
acaccctgccagggtacccagatttcctgtttggaacgatcccagttttagttctcaaagtccgacaggcctgggaaccccagtcccggaaaacccggacacgtgggc
ccgggctcccttaagtgttgccaggcctcagctggcttctgccagttgaaacctggctctggcctctcag
>IBE-C5383_anomalus_B
acaccctgccagggtacccagatttcctgtttggaacgatcccagttttagttctcaaagtccgacaggcctgggaaccccagtcccggaaaacccggacacgtgggc
ccgggctcccttaagtgttgccaggcctcagctggcttctgccagttgaaacctggctctggcctctcag
>IBE-C5697_niethammeri_A
acgccctgccagggtacccagatttcctgtttggaacgatcccagttttagttctcaaagtccgacaggcctgggaaccccagtcccggaaaacccagacacgtgggc
ctgggctcccttaagtgttgccacgcctcagctggcttctgccagttgaaccctggctctggcctctcag
>IBE-C5697_niethammeri_B
acgccctgccagggtacccagatttcctgtttggaacgatcccagttttagttctcaaagtccgacaggcctgggaaccccagtcccggaaaacccagacacgtgggc
ctgggctcccttaagtgttgccacgcctcagctggcttctgccagttgaaccctggctctggcctctcag
>IBE-C5700_anomalus_A
acaccctgccagggtacccagatttcctgtttggaacgatcccagttttagttctcaaagtccgacaggcctgggaaccccagtcccggaaaacccggacacgtgggc
ccgggctcccttaagtgatgccaggcctcagctggcttctgccagttgaaacctggctctggcctctcag
>IBE-C5700_anomalus_B
acaccctgccagggtacccagatttcctgtttggaacgatcccagttttagttctcaaagtccgacaggcctgggaaccccagtcccggaaaacccggacacgtgggc
ccgggctcccttaagtgttgccaggcctcagctggcttctgccagttgaaacctggctctggcctctcag
>IBE-C5701_niethammeri_A
acgccctgccagggtacccagatttcctgtttggaacgatcccagttttagttctcaaagtccgacaggcctgggaaccccagtcccggaaaacccagacacgtgggc
ctgggctcccttaagtgttgccacgcctcagctggcttctgccagttgaaccctggctctggcctctcag
>IBE-C5701_niethammeri_B
acgccctgccagggtacccagatttcctgtttggaacgatcccagttttagttctcaaagtccgacaggcctgggaaccccagtcccggaaaacccagacacgtgggc
ctgggctcccttaagtgttgccacgcctcagctggcttctgccagttgaaccctggctctggcctctcag
>IBE-C5603_niethammeri_A
acgccctgccagggtacccagatttcctgtttggaacgatcccagttttagttctcaaagtccgacaggcctgggaaccccagtcccggaaaacccggacacgtgggc
ctgggctcccttaagtgttgccaggcctcagctggcttctgccagttgaaccctggctctggcctctcag
>IBE-C5603_niethammeri_B
acgccctgccagggtacccagatttcctgtttggaacgatcccagttttagttctcaaagtccgacaggcctgggaaccccagtcccggaaaacccggacacgtgggc
ctgggctcccttaagtgttgccaggcctcagctggcttctgccagttgaaccctggctctggcctctcag
>IBE-C5604_niethammeri_A
acgccctgccagggtacccagatttcctgtttggaacgatcccagttttagttctcaaagtccgacaggcctgggaaccccagtcccggaaaacccagacacgtgggc
ctgggctcccttaagtgttgccacgcctcagctggcttctgccagttgaaccctggctctggcctctcag
>IBE-C5604_niethammeri_B
acgccctgccagggtacccagatttcctgtttggaacgatcccagttttagttctcaaagtccgacaggcctgggaaccccagtcccggaaaacccagacacgtgggc
ctgggctcccttaagtgttgccacgcctcagctggcttctgccagttgaaccctggctctggcctctcag
>IBE-C1927_milleri_A
acgccctgccagggtacccagatttcctgtttggaacgatcccagttttagttctcaaagtccgacaggcctgggaaccccagtcccggaaaacccggacacgtgggc
ccgggctcccttaagtgttgccaggcctcagctggcttctgccagttgaaacctggctctggcctctcag
>IBE-C1927_milleri_B
acgccctgccagggtacccagatttcctgtttggaacgatcccagttttagttctcaaagtccgacaggcctgggaaccccagtcccggaaaacccggacacgtgggc
ccgggctcccttaagtgttgccaggcctcagctggcttctgccagttgaaacctggctctggcctctcag
>IBE-C5370_fodiens_A
acgccctgccagggtacccagatttcctgtttggaacgatcccagttttagttctcaaagtccgacaggcctgggaaccccagtcccggaaaacccagacacgtgggc
ctgggctcccttaagtgttgccaggcctcagctggcttctgccagttgaaccctggctctggcctctcag
>IBE-C5370_fodiens_B
acgccctgccagggtacccagatttcctgtttggaacgatcccagttttagttctcaaagtccgacaggcctgggaaccccagtcccggaaaacccagacacgtgggc
ctgggctcccttaagtgttgccaggcctcagctggcttctgccagttgaaccctggctctggcctctcag
>IBE-C5719_anomalus_A
acaccctgccagggtacccagatttcctgtttggaacgatcccagttttagttctcaaagtccgacaggcctgggaaccccagtcccggaaaacccggacacgtgggc
ccgggctcccttaagtgatgccaggcctcagctggcttctgccagttgaaacctggctctggcctctcag
>IBE-C5719_anomalus_B
acaccctgccagggtacccagatttcctgtttggaacgatcccagttttagttctcaaagtccgacaggcctgggaaccccagtcccggaaaacccggacacgtgggc
ccgggctcccttaagtgatgccaggcctcagctggcttctgccagttgaaacctggctctggcctctcag
>IBE-C5720_anomalus_A
acaccctgccagggtacccagatttcctgtttggaacgatcccagttttagttctcaaagtccgacaggcctgggaaccccagtcccggaaaacccggacacgtgggc
ccgggctcccttaagtgttgccaggcctcagctggcttctgccagttgaaacctggctctggcctctcag
>IBE-C5720_anomalus_B
acaccctgccagggtacccagatttcctgtttggaacgatcccagttttagttctcaaagtccgacaggcctgggaaccccagtcccggaaaacccggacacgtgggc
ccgggctcccttaagtgatgccaggcctcagctggcttctgccagttgaaacctggctctggcctctcag
>IBE-C5699_niethammeri_A
acgccctgccagggtacccagatttcctgtttggaacgatcccagttttagttctcaaagtccgacaggcctgggaaccccagtcccggaaaacccggacacgtgggc
ctgggctcccttaagtgttgccaggcctcagctggcttctgccagttgaaccctggctctggcctctcag
>IBE-C5699_niethammeri_B
acgccctgccagggtacccagatttcctgtttggaacgatcccagttttagttctcaaagtccgacaggcctgggaaccccagtcccggaaaacccggacacgtgggc
ctgggctcccttaagtgttgccaggcctcagctggcttctgccagttgaaccctggctctggcctctcag
>UAM-24769_fodiens_A
acgccctgccagggtacccagatttcctgtttggaacgatcccagttttagttctcaaagtccgacaggcctgggaaccccagtcccggaaaacccggacacgtgggc
ctgggctcccttaagtgttgccaggcctcagctggcttctgccagttgaaccctggctctggcctctcag

>UAM-24769_fodiens_B
acgccctgccagggtacccagatttcctgtttggaacgatcccagttttagttctcaaagtccgacaggcctgggaaccccagtcccggaaaacccggacacgtgggc
ctgggctcccttaagtgttgccaggcctcagctggcttctgccagttgaaccctggctctggcctctcag
>UAM-24770_fodiens_A
acgccctgccagggtacccagatttcctgtttggaacgatcccagttttagttctcaaagtccgacaggcctgggaaccccagtcccggaaaacccggacacgtgggc
ctgggctcccttaagtgttgccaggcctcagctggcttctgccagttgaaccctggctctggcctctcag
>UAM-24770_fodiens_B
acgccctgccagggtacccagatttcctgtttggaacgatcccagttttagttctcaaagtccgacaggcctgggaaccccagtcccggaaaacccggacacgtgggc
ctgggctcccttaagtgttgccaggcctcagctggcttctgccagttgaaccctggctctggcctctcag
>IBE-C5372_niethammeri_A
acgccctgccagggtacccagatttcctgtttggaacgatcccagttttagttctcaaagtccgacaggcctgggaaccccagtcccggaaaacccggacacgtgggc
ctgggctcccttaagtgttgccaggcctcagctggcttctgccagttgaaccctggctctggcctctcag
>IBE-C5372_niethammeri_B
acgccctgccagggtacccagatttcctgtttggaacgatcccagttttagttctcaaagtccgacaggcctgggaaccccagtcccggaaaacccagacacgtgggc
ctgggctcccttaagtgttgccacgcctcagctggcttctgccagttgaaccctggctctggcctctcag
>IBE-C5374_fodiens_A
acgccctgccagggtacccagatttcctgtttggaacgatcccagttttagttctcaaagtccgacaggcctgggaaccccagtcccggaaaacccagacacgtgggc
ctgggctcccttaagtgttgccacgcctcagctggcttctgccagttgaaccctggctctggcctctcag
>IBE-C5374_fodiens_B
acgccctgccagggtacccagatttcctgtttggaacgatcccagttttagttctcaaagtccgacaggcctgggaaccccagtcccggaaaacccagacacgtgggc
ctgggctcccttaagtgttgccacgcctcagctggcttctgccagttgaaccctggctctggcctctcag
>IBE-C5376_fodiens_A
acgccctgccagggtacccagatttcctgtttggaacgatcccagttttagttctcaaagtccgacaggcctgggaaccccagtcccggaaaacccggacacgtgggc
ctgggctcccttaagtgttgccaggcctcagctggcttctgccagttgaaccctggctctggcctctcag
>IBE-C5376_fodiens_B
acgccctgccagggtacccagatttcctgtttggaacgatcccagttttagttctcaaagtccgacaggcctgggaaccccagtcccggaaaacccagacacgtgggc
ctgggctcccttaagtgttgccacgcctcagctggcttctgccagttgaaccctggctctggcctctcag
>IBE-C5726_anomalus_A
acaccctgccagggtacccagatttcctgtttggaacgatcccagttttagttctcaaagtccgacaggcctgggaaccccagtcccggaaaacccggacacgtgggc
ccgggctcccttaagtgttgccaggcctcagctggcttctgccagttgaaacctggctctggcctctcag
>IBE-C5726_anomalus_B
acaccctgccagggtacccagatttcctgtttggaacgatcccagttttagttctcaaagtccgacaggcctgggaaccccagtcccggaaaacccggacacgtgggc
ccgggctcccttaagtgttgccaggcctcagctggcttctgccagttgaaacctggctctggcctctcag
>IBE-C5744_fodiens_A
acgccctgccagggtacccagatttcctgtttggaacgatcccagttttagttctcaaagtccgacaggcctgggaaccccagtcccggaaaacccggacacgtgggc
ctgggctcccttaagtgttgccaggcctcagctggcttctgccagttgaaccctggctctggcctctcag
>IBE-C5744_fodiens_B
acgccctgccagggtacccagatttcctgtttggaacgatcccagttttagttctcaaagtccgacaggcctgggaaccccagtcccggaaaacccagacacgtgggc
ctgggctcccttaagtgttgccacgcctcagctggcttctgccagttgaaccctggctctggcctctcag
>IBE-C6063_fodiens_A
acgccctgccagggtacccagatttcctgtttggaacgatcccagttttagttctcaaagtccgacaggcctgggaaccccagtcccggaaaacccagacacgtgggc
ctgggctcccttaagtgttgccaggcctcagctggcttctgccagttgaaccctggctctggcctctcag
>IBE-C6063_fodiens_B
acgccctgccagggtacccagatttcctgtttggaacgatcccagttttagttctcaaagtccgacaggcctgggaaccccagtcccggaaaacccagacacgtgggc
ctgggctcccttaagtgttgccaggcctcagctggcttctgccagttgaaccctggctctggcctctcag
>IBE-C6064_fodiens_A
acgccctgccagggtacccagatttcctgtttggaacgatcccagttttagttctcaaagtccgacaggcctgggaaccccagtcccggaaaacccggacacgtgggc
ctgggctcccttaagtgttgccaggcctcagctggcttctgccagttgaaccctggctctggcctctcag
>IBE-C6064_fodiens_B
acgccctgccagggtacccagatttcctgtttggaacgatcccagttttagttctcaaagtccgacaggcctgggaaccccagtcccggaaaacccggacacgtgggc
ctgggctcccttaagtgttgccaggcctcagctggcttctgccagttgaaccctggctctggcctctcag
>IBE-C6065_anomalus_A
acaccctgccagggtacccagatttcctgtttggaacgatcccagttttagttctcaaagtccgacaggcctgggaaccccagtcccggaaaacccggacacgtgggc
ccgggctcccttaagtgatgccaggcctcagctggcttctgccagttgaaacctggctctggcctctcag
>IBE-C6065_anomalus_B
acaccctgccagggtacccagatttcctgtttggaacgatcccagttttagttctcaaagtccgacaggcctgggaaccccagtcccggaaaacccggacacgtgggc
ccgggctcccttaagtgatgccaggcctcagctggcttctgccagttgaaacctggctctggcctctcag
>IBE-C6066_fodiens_A
acgccctgccagggtacccagatttcctgtttggaacgatcccagttttagttctcaaagtccgacaggcctgggaaccccagtcccggaaaacccagacacgtgggc
ctgggctcccttaagtgttgccacgcctcagctggcttctgccagttgaaccctggctctggcctctcag
>IBE-C6066_fodiens_B
acgccctgccagggtacccagatttcctgtttggaacgatcccagttttagttctcaaagtccgacaggcctgggaaccccagtcccggaaaacccagacacgtgggc
ctgggctcccttaagtgttgccacgcctcagctggcttctgccagttgaaccctggctctggcctctcag
>IBE-C6069_fodiens_A
acgccctgccagggtacccagatttcctgtttggaacgatcccagttttagttctcaaagtccgacaggcctgggaaccccagtcccggaaaacccggacacgtgggc
ctgggctcccttaagtgttgccaggcctcagctggcttctgccagttgaaccctggctctggcctctcag
>IBE-C6069_fodiens_B
acgccctgccagggtacccagatttcctgtttggaacgatcccagttttagttctcaaagtccgacaggcctgggaaccccagtcccggaaaacccagacacgtgggc
ctgggctcccttaagtgttgccacgcctcagctggcttctgccagttgaaccctggctctggcctctcag
>IBE-C4117_fodiens_A
acgccctgccagggtacccagatttcctgtttggaacgatcccagttttagttctcaaagtccgacaggcctgggaaccccagtcccggaaaacccggacacgtgggc
ctgggctcccttaagtgttgccaggcctcagctggcttctgccagttgaaccctggctctggcctctcag
>IBE-C4117_fodiens_B

acgccctgccagggtacccagatttcctgtttggaacgatcccagttttagttctcaaagtccgacaggcctgggaaccccagtcccggaaaacccggacacgtgggc
ctgggctcccttaagtgttgccaggcctcagctggcttctgccagttgaaccctggctctggcctctcag
>IBE-C6105_niethammeri_A
acgccctgccagggtacccagatttcctgtttggaacgatcccagttttagttctcaaagtccgacaggcctgggaaccccagtcccggaaaacccggacacgtgggc
ctgggctcccttaagtgttgccaggcctcagctggcttctgccagttgaaccctggctctggcctctcag
>IBE-C6105_niethammeri_B
acgccctgccagggtacccagatttcctgtttggaacgatcccagttttagttctcaaagtccgacaggcctgggaaccccagtcccggaaaacccggacacgtgggc
ctgggctcccttaagtgttgccaggcctcagctggcttctgccagttgaaccctggctctggcctctcag
>IBE-C6106_niethammeri_A
acgccctgccagggtacccagatttcctgtttggaacgatcccagttttagttctcaaagtccgacaggcctgggaaccccagtcccggaaaacccagacacgtgggc
ctgggctcccttaagtgttgccacgcctcagctggcttctgccagttgaaccctggctctggcctctcag
>IBE-C6106_niethammeri_B
acgccctgccagggtacccagatttcctgtttggaacgatcccagttttagttctcaaagtccgacaggcctgggaaccccagtcccggaaaacccagacacgtgggc
ctgggctcccttaagtgttgccacgcctcagctggcttctgccagttgaaccctggctctggcctctcag
>IBE-C6118_anomalus_A
acaccctgccagggtacccagatttcctgtttggaacgatcccagttttagttctcaaagtccgacaggcctgggaaccccagtcccggaaaacccggacacgtgggc
ccgggctcccttaagtgttgccaggcctcagctggcttctgccagttgaaacctggctctggcctctcag
>IBE-C6118_anomalus_B
acaccctgccagggtacccagatttcctgtttggaacgatcccagttttagttctcaaagtccgacaggcctgggaaccccagtcccggaaaacccggacacgtgggc
ccgggctcccttaagtgttgccaggcctcagctggcttctgccagttgaaacctggctctggcctctcag
>IBE-C6121_anomalus_A
acaccctgccagggtacccagatttcctgtttggaacgatcccagttttagttctcaaagtccgacaggcctgggaaccccagtcccggaaaacccggacacgtgggc
ccgggctcccttaagtgttgccaggcctcagctggcttctgccagttgaaacctggctctggcctctcag
>IBE-C6121_anomalus_B
acaccctgccagggtacccagatttcctgtttggaacgatcccagttttagttctcaaagtccgacaggcctgggaaccccagtcccggaaaacccggacacgtgggc
ccgggctcccttaagtgttgccaggcctcagctggcttctgccagttgaaacctggctctggcctctcag
>IBE-C6110_niethammeri_A
acgccctgccagggtacccagatttcctgtttggaacgatcccagttttagttctcaaagtccgacaggcctgggaaccccagtcccggaaaacccggacacgtgggc
ctgggctcccttaagtgttgccaggcctcagctggcttctgccagttgaaccctggctctggcctctcag
>IBE-C6110_niethammeri_B
acgccctgccagggtacccagatttcctgtttggaacgatcccagttttagttctcaaagtccgacaggcctgggaaccccagtcccggaaaacccggacacgtgggc
ctgggctcccttaagtgttgccaggcctcagctggcttctgccagttgaaccctggctctggcctctcag
>IBE-C6116_niethammeri_A
acgccctgccagggtacccagatttcctgtttggaacgatcccagttttagttctcaaagtccgacaggcctgggaaccccagtcccggaaaacccggacacgtgggc
ctgggctcccttaagtgttgccaggcctcagctggcttctgccagttgaaccctggctctggcctctcag
>IBE-C6116_niethammeri_B
acgccctgccagggtacccagatttcctgtttggaacgatcccagttttagttctcaaagtccgacaggcctgggaaccccagtcccggaaaacccagacacgtgggc
ctgggctcccttaagtgttgccacgcctcagctggcttctgccagttgaaccctggctctggcctctcag
>IBE-C6124_anomalus_A
acaccctgccagggtacccagatttcctgtttggaacgatcccagttttagttctcaaagtccgacaggcctgggaaccccagtcccggaaaacccggacacgtgggc
ccgggctcccttaagtgatgccaggcctcagctggcttctgccagttgaaacctggctctggcctctcag
>IBE-C6124_anomalus_B
acaccctgccagggtacccagatttcctgtttggaacgatcccagttttagttctcaaagtccgacaggcctgggaaccccagtcccggaaaacccggacacgtgggc
ccgggctcccttaagtgttgccaggcctcagctggcttctgccagttgaaacctggctctggcctctcag
>IBE-C6125_anomalus_A
acaccctgccagggtacccagatttcctgtttggaacgatcccagttttagttctcaaagtccgacaggcctgggaaccccagtcccggaaaacccggacacgtgggc
ccgggctcccttaagtgatgccaggcctcagctggcttctgccagttgaaacctggctctggcctctcag
>IBE-C6125_anomalus_B
acaccctgccagggtacccagatttcctgtttggaacgatcccagttttagttctcaaagtccgacaggcctgggaaccccagtcccggaaaacccggacacgtgggc
ccgggctcccttaagtgttgccaggcctcagctggcttctgccagttgaaacctggctctggcctctcag
>MSB-158495_fodiens_A
acgccctgccagggtacccagatttcctgtttggaacgatcccagttttagttctcaaagtccgacaggcctgggaaccccagtcccggaaaacccggacacgtgggc
ctgggctcccttaagtgttgccaggcctcagctggcttctgccagttgaaccctggctctggcctctcag
>MSB-158495_fodiens_B
acgccctgccagggtacccagatttcctgtttggaacgatcccagttttagttctcaaagtccgacaggcctgggaaccccagtcccggaaaacccggacacgtgggc
ctgggctcccttaagtgttgccaggcctcagctggcttctgccagttgaaccctggctctggcctctcag
>MSB-158621_fodiens_A
acgccctgccagggtacccagatttcctgtttggaacgatcccagttttagttctcaaagtccgacaggcctgggaaccccagtcccggaaaacccggacacgtgggc
ctgggctcccttaagtgttgccaggcctcagctggcttctgccagttgaaccctggctctggcctctcag
>MSB-158621_fodiens_B
acgccctgccagggtacccagatttcctgtttggaacgatcccagttttagttctcaaagtccgacaggcctgggaaccccagtcccggaaaacccggacacgtgggc
ctgggctcccttaagtgttgccaggcctcagctggcttctgccagttgaaccctggctctggcctctcag
>MSB-288698_fodiens_A
acgccctgccagggtacccagatttcctgtttggaacgatcccagttttagttctcaaagtccgacaggcctgggaaccccagtcccggaaaacccggacacgtgggc
ctgggctcccttaagtgttgccaggcctcagctggcttctgccagttgaaccctggctctggcctctcag
>MSB-288698_fodiens_B
acgccctgccagggtacccagatttcctgtttggaacgatcccagttttagttctcaaagtccgacaggcctgggaaccccagtcccggaaaacccggacacgtgggc
ctgggctcccttaagtgttgccaggcctcagctggcttctgccagttgaaccctggctctggcctctcag
>MSB-293526_fodiens_A
acgccctgccagggtacccagatttcctgtttggaacgatcccagttttagttctcaaagtccgacaggcctgggaaccccagtcccggaaaacccggacacgtgggc
ctgggctcccttaagtgttgccaggcctcagctggcttctgccagttgaaccctggctctggcctctcag
>MSB-293526_fodiens_B
acgccctgccagggtacccagatttcctgtttggaacgatcccagttttagttctcaaagtccgacaggcctgggaaccccagtcccggaaaacccggacacgtgggc
ctgggctcccttaagtgttgccaggcctcagctggcttctgccagttgaaccctggctctggcctctcag

>MSB-95472_fodiens_A
acgccctgccagggtacccagatttcctgtttggaacgatcccagttttagttctcaaagtccgacaggcctgggaaccccagtcccggaaaacccggacacgtgggc
ctgggctcccttaagtgttgccaggcctcagctggcttctgccagttgaaccctggctctggcctctcag
>MSB-95472_fodiens_B
acgccctgccagggtacccagatttcctgtttggaatgatcccagttttagttctcaaagtccgacaggcctgggaaccccagtcccggaaaacccggacacgtgggc
ctgggctcccttaagtgttgccaggcctcagctggcttctgccagttgaaccctggctctggcctctcag
>MSB-288577_fodiens_A
acgccctgccagggtacccagatttcctgtttggaacgatcccagttttagttctcaaagtccgacaggcctgggaaccccagtcccggaaaacccggacacgtgggc
ctgggctcccttaagtgttgccaggcctcagctggcttctgccagttgaaccctggctctggcctctcag
>MSB-288577_fodiens_B
acgccctgccagggtacccagatttcctgtttggaacgatcccagttttagttctcaaagtccgacaggcctgggaaccccagtcccggaaaacccggacacgtgggc
ctgggctcccttaagtgttgccaggcctcagctggcttctgccagttgaaccctggctctggcctctcag
>IBE-C5371_niethammeri_A
acgccctgccagggtacccagatttcctgtttggaacgatcccagttttagttctcaaagtccgacaggcctgggaaccccagtcccggaaaacccagacacgtgggc
ctgggctcccttaagtgttgccacgcctcagctggcttctgccagttgaaccctggctctggcctctcag
>IBE-C5371_niethammeri_B
acgccctgccagggtacccagatttcctgtttggaacgatcccagttttagttctcaaagtccgacaggcctgggaaccccagtcccggaaaacccggacacgtgggc
ctgggctcccttaagtgttgccaggcctcagctggcttctgccagttgaaccctggctctggcctctcag
>IBE-C6108_niethammeri_A
acaccctgccagggtacccagatttcctgtttggaacgatcccagttttagttctcaaagtccgacaggcctgggaaccccagtcccggaaaacccagacacgtgggc
ctgggctcccttaagtgttgccacgcctcagctggcttctgccagttgaaccctggctctggcctctcag
>IBE-C6108_niethammeri_B
acgccctgccagggtacccagatttcctgtttggaacgatcccagttttagttctcaaagtccgacaggcctgggaaccccagtcccggaaaacccggacacgtgggc
ctgggctcccttaagtgttgccaggcctcagctggcttctgccagttgaaccctggctctggcctctcag
>IBE-C5742_fodiens_A
acgccctgccagggtacccagatttcctgtttggaacgatcccagttttagttctcaaagtccgacaggcctgggaaccccagtcccggaaaacccggacacgtgggc
ctgggctcccttaagtgttgccaggcctcagctggcttctgccagttgaaccctggctctggcctctcag
>IBE-C5742_fodiens_B
acgccctgccagggtacccagatttcctgtttggaacgatcccagttttagttctcaaagtccgacaggcctgggaaccccagtcccggaaaacccggacacgtgggc
ctgggctcccttaagtgttgccaggcctcagctggcttctgccagttgaaccctggctctggcctctcag
>IBE-C5188_fodiens_A
acgccctgccagggtacccagatttcctgtttggaacgatcccagttttagttctcaaagtccgacaggcctgggaaccccagtcccggaaaacccagacacgtgggc
ctgggctcccttaagtgttgccacgcctcagctggcttctgccagttgaaccctggctctggcctctcag
>IBE-C5188_fodiens_B
acgccctgccagggtacccagatttcctgtttggaacgatcccagttttagttctcaaagtccgacaggcctgggaaccccagtcccggaaaacccagacacgtgggc
ctgggctcccttaagtgttgccacgcctcagctggcttctgccagttgaaccctggctctggcctctcag
>IBE-C5741_niethammeri_A
acgccctgccagggtacccagatttcctgtttggaacgatcccagttttagttctcaaagtccgacaggcctgggaaccccagtcccggaaaacccggacacgtgggc
ctgggctcccttaagtgttgccaggcctcagctggcttctgccagttgaaccctggctctggcctctcag
>IBE-C5741_niethammeri_B
acgccctgccagggtacccagatttcctgtttggaacgatcccagttttagttctcaaagtccgacaggcctgggaaccccagtcccggaaaacccggacacgtgggc
ctgggctcccttaagtgttgccaggcctcagctggcttctgccagttgaaccctggctctggcctctcag
>IBE-C4515_anomalus_A
acaccctgccagggtacccagatttcctgtttggaacgatcccagttttagttctcaaagtccgacaggcctgggaaccccagtcccggaaaacccggacacgtgggc
ccgggctcccttaagtgatgccaggcctcagctggcttctgccagttgaaacctggctctggcctctcag
>IBE-C4515_anomalus_B
acaccctgccagggtacccagatttcctgtttggaacgatcccagttttagttctcaaagtccgacaggcctgggaaccccagtcccggaaaacccggacacgtgggc
ccgggctcccttaagtgttgccaggcctcagctggcttctgccagttgaaacctggctctggcctctcag

TRAIP-intron-8-oneseq.fasta
>IBE-C1529_anomalus_A
ggctggggaagcacaggttccaaatgtgatgatgttagaggccaggtttcgtccagcagaggaggaccattggaacctgattcagcctgcacagctgctttcatttct
ggccatgtacatccatgggctgcaggtgcaggaagtagcccttgctcctgggaagctccatagggacactgccatctgaaagccccg
>IBE-C1529_anomalus_B
ggctggggaagcacaggttccaaatgtgatgatgttagaggccaggtttcgtccagcagaggaggaccattggaacctgattcagcctgcacagctgctttcatttct
ggccatgtacatccatgggctgcaggtgcaggaagtagcccttgctcctgggaagctccatagggacactgccatctgaaagccccg
>IBE-C1789_anomalus_A
ggctggggaagcacaggttccaaatgtgatgatgttagaggccaggtttcgtccagcagaggaggaccattggaacctgattcagcctgcacagctgctttcatttct
ggccatgtacatccatgggctgcaggtgcaggaagtagcccttgctcctgggaagctccatagggacactgccatctgaaagcccca
>IBE-C1789_anomalus_B
ggctggggaagcacaggttccaaatgtgatgatgttagaggccaggtttcgtccagcagaggaggaccattggaacctgattcagcctgcacagctgctttcatttct
ggccatgtacatccatgggctgcaggtgcaggaagtagcccttgctcctgggaagctccatagggacactgccatctgaaagccccg
>IBE-C1808_milleri_A
ggctggggaagcacaggttccaaatgtgatgatgttagaggccaggtttcgtccagcagaggaggaccattggaacctgattcagcctgcacagctgctttcatttct
ggccatgtacatccatgggctgcaggtgcaggaagtagcccttgctcctgggaagctccatagggacactgccatctgaaagcccca
>IBE-C1808_milleri_B
ggctggggaagcacaggttccaaatgtgatgatgttagaggccaggtttcgtccagcagaggaggaccattggaacctgattcagcctgcacagctgctttcatttct
ggccatgtacatccatgggctgcaggtgcaggaagtagcccttgctcctgggaagctccatagggacactgccatctgaaagcccca
>IBE-C2895_anomalus_A
ggctggggaagcacaggttccaaatgtgatgatgttagaggccaggtttcgtccagcagaggaggaccattggaacctgattcagcctgcacagctgctttcatttct
ggccatgtacatccatgggctgcaggtgcaggaagtagcccttgctcctgggaagctccatagggacactgccatctgaaagccccg
>IBE-C2895_anomalus_B
ggctggggaagcacaggttccaaatgtgatgatgttagaggccaggtttcgtccagcagaggaggaccattggaacctgattcagcctgcacagctgctttcatttct
ggccatgtacatccatgggctgcaggtgcaggaagtagcccttgctcctgggaagctccatagggacactgccatctgaaagccccg
>IBE-C3786_milleri_A
ggctggggaagcacaggttccaaatgtgatgatgttagaggccaggtttcgtccagcagaggaggaccattggaacctgattcagcctgcacagctgctttcatttct
ggccatgtacatccatgggctgcaggtgcaggaagtagcccttgctcctgggaagctccatagggacactgccatctgaaagcccca
>IBE-C3786_milleri_B
ggctggggaagcacaggttccaaatgtgatgatgttagaggccaggtttcgtccagcagaggaggaccattggaacctgattcagcctgcacagctgctttcatttct
ggccatgtacatccatgggctgcaggtgcaggaagtagcccttgctcctgggaagctccatagggacactgccatctgaaagcccca
>IBE-C4116_milleri_A
ggctggggaagcacaggttccaaatgtgatgatgttagaggccaggtttcgtccagcagaggaggaccattggaacctgattcagcctgcacagctgctttcatttct
ggccatgtacatccatgggctgcaggtgcaggaagtagcccttgcacctgggaagctccatagggacactgccatctgaaagcccca
>IBE-C4116_milleri_B
ggctggggaagcacaggttccaaatgtgatgatgttagaggccaggtttcgtccagcagaggaggaccattggaacctgattcagcctgcacagctgctttcatttct
ggccatgtacatccatgggctgcaggtgcaggaagtagcccttgcacctgggaagctccatagggacactgccatctgaaagcccca
>IBE-C101_fodiens_A
ggctggggaagcacaggttccaaatgtgatgatgttagaggccgggtttcgtccagcagaggaggaccattggaacctgattcagcctgcacagctgctttcatttct
ggccatgtatatccatgggctgcaggtgcaggaagtagcccttgctcctgggaagctccatagggacactgccagctgaaagcccca
>IBE-C101_fodiens_B
ggctggggaagcacaggttccaaatgtgatgatgttagaggccgggtttcgtccagcagaggaggaccattggaacctgattcagcctgcacagctgctttcatttct
ggccatgtatatccatgggctgcaggtgcaggaagtagcccttgctcctgggaagctccatagggacactgccagctgaaagcccca
>IBE-C1914_fodiens_A
ggctggggaagcacaggttccaaatgtgatgatgttagaggccgggtttcgtccagcagaggaggaccattggaacctgattcagcctgcacagctgctttcatttct
ggccatgtatatccatgggctgcaggtgcaggaagtagcccttgctcctgggaagctccatagggacactgccagctgaaagcccca
>IBE-C1914_fodiens_B
ggctggggaagcacaggttccaaatgtgatgatgttagaggccgggtttcgtccagcagaggaggaccattggaacctgattcagcctgcacagctgctttcatttct
ggccatgtatatccatgggctgcaggtgcaggaagtagcccttgctcctgggaagctccatagggacactgccagctgaaagcccca
>IBE-C4120_teres_A
ggctgaggaagcacaggttccaaatgtgatgatgttagaggccaggtttcgtccagcagaggaggaccattggaacctgattcagcctgcacagctgctttcatttct
ggccatgtacatccatgggctgcaggtgcaggaagtagcccttgctcctgggaagctccatagggacactgccatctgaaagcccca
>IBE-C4120_teres_B
ggctgaggaagcacaggttccaaatgtgatgatgttagaggccaggtttcgtccagcagaggaggaccattggaacctgattcagcctgcacagctgctttcatttct
ggccatgtacatccatgggctgcaggtgcaggaagtagcccttgctcctgggaagctccatagggacactgccatctgaaagcccca
>IBE-C4122_teres_A
ggctgaggaagcacaggttccaaatgtgatgatgttagaggccaggtttcgtccagcagaggaggaccattggaacctgattcagcctgcacagctgctttcatttct
ggccatgtacatccatgggctgcaggtgcaggaagtagcccttgctcctgggaagctccatagggacactgccatctgaaagcccca
>IBE-C4122_teres_B
ggctgaggaagcacaggttccaaatgtgatgatgttagaggccaggtttcgtccagcagaggaggaccattggaacctgattcagcctgcacagctgctttcatttct
ggccatgtacatccatgggctgcaggtgcaggaagtagcccttgctcctgggaagctccatagggacactgccatctgaaagcccca
>IBE-C5182_niethammeri_A
ggctggggaagcacaggttccaaatgtgatgatgttagaggccgggtttcgtccagcagaggaggaccattggaacctgattcagcctgcacagctgctttcatttct
ggccatgtatatccatgggctgcaggtgcaggaagtagcccttgctcctgggaagctccatagggacactgccagctgaaagcccca
>IBE-C5182_niethammeri_B
ggctggggaagcacaggttccaaatgtgatgatgttagaggccgggtttcgtccagcagaggaggaccattggaacctgattcagcctgcacagctgctttcatttct
ggccatgtatatccatgggctgcaggtgcaggaagtagcccttgctcctgggaagctccatagggacactgccagctgaaagcccca
>IBE-C5368_niethammeri_A
ggctggggaagcacaggttccaaatgtgatgatgttagaggccgggtttcgtccagcagaggaggaccattggaacctgattcagcctgcacagctgctttcatttct
ggccatgtatatccatgggctgcaggtgcaggaagtagcccttgctcctgggaagctccatagggacactgccagctgaaagcccca
>IBE-C5368_niethammeri_B
ggctggggaagcacaggttccaaatgtgatgatgttagaggccgggtttcgtccagcagaggaggaccattggaacctgattcagcctgcacagctgctttcatttct
ggccatgtatatccatgggctgcaggtgcaggaagtagcccttgctcctgggaagctccatagggacactgccagctgaaagcccca
>IBE-C5700_anomalus_A

ggctggggaagcacaggttccaaatgtgatgatgttagaggccaggtttcgtccagcagaggaggaccattggaacctgattcagcctgcacagctgctttcatttct
ggccatgtacatccatgggctgcaggtgcaggaagtagcccttgctcctgggaagctccatagggacactgccatctgaaagccccg
>IBE-C5700_anomalus_B
ggctggggaagcacaggttccaaatgtgatgatgttagaggccaggtttcgtccagcagaggaggaccattggaacctgattcagcctgcacagctgctttcatttct
ggccatgtacatccatgggctgcaggtgcaggaagtagcccttgctcctgggaagctccatagggacactgccatctgaaagccccg
>IBE-C5701_niethammeri_A
ggctggggaagcacaggttccaaatgtgatgatgttagaggccgggtttcgtccagcagaggaggaccattggaacctgattcagcctgcacagctgctttcatttct
ggccatgtatatccatgggctgcaggtgcaggaagtagcccttgctcctgggaagctccatagggacactgccagctgaaagcccca
>IBE-C5701_niethammeri_B
ggctggggaagcacaggttccaaatgtgatgatgttagaggccgggtttcgtccagcagaggaggaccattggaacctgattcagcctgcacagctgctttcatttct
ggccatgtatatccatgggctgcaggtgcaggaagtagcccttgctcctgggaagctccatagggacactgccagctgaaagcccca
>IBE-C5370_fodiens_A
ggctggggaagcacaggttccaaatgtgatgatgttagaggccgggtttcgtccagcagaggaggaccattggaacctgattcagcctgcacagctgctttcatttct
ggccatgtatatccatgggctgcaggtgcaggaagtagcccttgctcctgggaagctccatagggacactgccagctgaaagcccca
>IBE-C5370_fodiens_B
ggctggggaagcacaggttccaaatgtgatgatgttagaggccgggtttcgtccagcagaggaggaccattggaacctgattcagcctgcacagctgctttcatttct
ggccatgtatatccatgggctgcaggtgcaggaagtagcccttgctcctgggaagctccatagggacactgccagctgaaagcccca
>IBE-C5383_anomalus_A
ggctggggaagcacaggttccaaatgtgatgatgttagaggccaggtttcgtccagcagaggaggaccattggaacctgattcagcctgcacagctgctttcatttct
ggccatgtacatccatgggctgcaggtgcaggaagtagcccttgctcctgggaagctccatagggacactgccatctgaaagccccg
>IBE-C5383_anomalus_B
ggctggggaagcacaggttccaaatgtgatgatgttagaggccaggtttcgtccagcagaggaggaccattggaacctgattcagcctgcacagctgctttcatttct
ggccatgtacatccatgggctgcaggtgcaggaagtagcccttgctcctgggaagctccatagggacactgccatctgaaagccccg
>IBE-C5603_niethammeri_A
ggctggggaagcacaggttccaaatgtgatgatgttagaggccgggtttcgtccagcagaggaggaccattggaacctgattcagcctgcacagctgctttcatttct
ggccatgtatatccatgggctgcaggtgcaggaagtagcccttgctcctgggaagctccatagggacactgccagctgaaagcccca
>IBE-C5603_niethammeri_B
ggctggggaagcacaggttccaaatgtgatgatgttagaggccgggtttcgtccagcagaggaggaccattggaacctgattcagcctgcacagctgctttcatttct
ggccatgtatatccatgggctgcaggtgcaggaagtagcccttgctcctgggaagctccatagggacactgccagctgaaagcccca
>IBE-C5604_niethammeri_A
ggctggggaagcacaggttccaaatgtgatgatgttagaggccgggtttcgtccagcagaggaggaccattggaacctgattcagcctgcacagctgctttcatttct
ggccatgtatatccatgggctgcaggtgcaggaagtagcccttgctcctgggaagctccatagggacactgccagctgaaagcccca
>IBE-C5604_niethammeri_B
ggctggggaagcacaggttccaaatgtgatgatgttagaggccgggtttcgtccagcagaggaggaccattggaacctgattcagcctgcacagctgctttcatttct
ggccatgtatatccatgggctgcaggtgcaggaagtagcccttgctcctgggaagctccatagggacactgccagctgaaagcccca
>IBE-C1927_milleri_A
ggctggggaagcacaggttccaaatgtgatgatgttagaggccaggtttcgtccagcagaggaggaccattggaacctgattcagcctgcacagctgctttcatttct
ggccatgtacatccatgggctgcaggtgcaggaagtagcccttgctcctgggaagctccatagggacactgccatctgaaagcccca
>IBE-C1927_milleri_B
ggctggggaagcacaggttccaaatgtgatgatgttagaggccaggtttcgtccagcagaggaggaccattggaacctgattcagcctgcacagctgctttcatttct
ggccatgtacatccatgggctgcaggtgcaggaagtagcccttgctcctgggaagctccatagggacactgccatctgaaagcccca
>IBE-C5367_fodiens_A
ggctggggaagcacaggttccaaatgtgatgatgttagaggccgggtttcgtccagcagaggaggaccattggaacctgattcagcctgcacagctgctttcatttct
ggccatgtatatccatgggctgcaggtgcaggaagtagcccttgctcctgggaagctccatagggacactgccagctgaaagcccca
>IBE-C5367_fodiens_B
ggctggggaagcacaggttccaaatgtgatgatgttagaggccgggtttcgtccagcagaggaggaccattggaacctgattcagcctgcacagctgctttcatttct
ggccatgtatatccatgggctgcaggtgcaggaagtagcccttgctcctgggaagctccatagggacactgccagctgaaagcccca
>IBE-C5697_niethammeri_A
ggctggggaagcacaggttccaaatgtgatgatgttagaggccgggtttcgtccagcagaggaggaccattggaacctgattcagcctgcacagctgctttcatttct
ggccatgtatatccatgggctgcaggtgcaggaagtagcccttgctcctgggaagctccatagggacactgccagctgaaagcccca
>IBE-C5697_niethammeri_B
ggctggggaagcacaggttccaaatgtgatgatgttagaggccgggtttcgtccagcagaggaggaccattggaacctgattcagcctgcacagctgctttcatttct
ggccatgtatatccatgggctgcaggtgcaggaagtagcccttgctcctgggaagctccatagggacactgccagctgaaagcccca
>IBE-C5719_anomalus_A
ggctggggaagcacaggttccaaatgtgatgatgttagaggccaggtttcgtccagcagaggaggaccattggaacctgattcagcctgcacagctgctttcatttct
ggccatgtacatccatgggctgcaggtgcaggaagtagcccttgctcctgggaagctccatagggacactgccatctgaaagcccca
>IBE-C5719_anomalus_B
ggctggggaagcacaggttccaaatgtgatgatgttagaggccaggtttcgtccagcagaggaggaccattggaacctgattcagcctgcacagctgctttcatttct
ggccatgtacatccatgggctgcaggtgcaggaagtagcccttgctcctgggaagctccatagggacactgccatctgaaagccccg
>IBE-C5720_anomalus_A
ggctggggaagcacaggttccaaatgtgatgatgttagaggccaggtttcgtccagcagaggaggaccattggaacctgattcagcctgcacagctgctttcatttct
ggccatgtacatccatgggctgcaggtgcaggaagtagcccttgctcctgggaagctccatagggacactgccatctgaaagccccg
>IBE-C5720_anomalus_B
ggctggggaagcacaggttccaaatgtgatgatgttagaggccaggtttcgtccagcagaggaggaccattggaacctgattcagcctgcacagctgctttcatttct
ggccatgtacatccatgggctgcaggtgcaggaagtagcccttgctcctgggaagctccatggggacactgccatctgaaagccccg
>IBE-C5699_niethammeri_A
ggctggggaagcacaggttccaaatgtgatgatgttagaggccgggtttcttccagcagaggaggaccattggaacctgattcagcctgcacagctgctttcatttct
ggccatgtatatccatgggctgcaggtgcaggaagtagcccttgctcctgggaagctccatagggacactgccagctgaaagcccca
>IBE-C5699_niethammeri_B
ggctggggaagcacaggttccaaatgtgatgatgttagaggccgggtttcgtccagcagaggaggaccattggaacctgattcagcctgcacagctgctttcatttct
ggccatgtatatccatgggctgcaggtgcaggaagtagcccttgctcctgggaagctccatagggacactgccagctgaaagcccca
>IBE-C5741_niethammeri_A
ggctggggaagcacaggttccaaatgtgatgatgttagaggccgggtttcgtccagcagaggaggaccattggaacctgattcagcctgcacagctgctttcatttct
ggccatgtatatccatgggctgcaggtgcaggaagtagcccttgctcctgggaagctccatagggacactgccagctgaaagcccca

>IBE-C5741_niethammeri_B
ggctggggaagcacaggttccaaatgtgatgatgttagaggccgggtttcgtccagcagaggaggaccattggaacctgattcagcctgcacagctgctttcatttct
ggccatgtatatccatgggctgcaggtgcaggaagtagcccttgctcctgggaagctccatagggacactgccagctgaaagcccca
>UAM-24769_fodiens_A
ggctggggaagcacaggttccaaatgtgatgatgttagaggccgggtttcgtccagcagaggaggaccattggaacctgattcagcctgcacagctgctttcatttct
ggccatgtatatccatgggctgcaggtgcaggaagtagcccttgctcctgggaagctccatagggacactgccagctgaaagcccca
>UAM-24769_fodiens_B
ggctggggaagcacaggttccaaatgtgatgatgttagaggccgggtttcgtccagcagaggaggaccattggaacctgattcagcctgcacagctgctttcatttct
ggccatgtatatccatgggctgcaggtgcaggaagtagcccttgctcctgggaagctccatagggacactgccagctgaaagcccca
>UAM-24770_fodiens_A
ggctggggaagcacaggttccaaatgtgatgatgttagaggccgggtttcgtccagcagaggaggaccattggaacctgattcagcctgcacagctgctttcatttct
ggccatgtatatccatgggctgcaggtgcaggaagtagcccttgctcctgggaagctccatagggacactgccagctgaaagcccca
>UAM-24770_fodiens_B
ggctggggaagcacaggttccaaatgtgatgatgttagaggccgggtttcgtccagcagaggaggaccattggaacctgattcagcctgcacagctgctttcatttct
ggccatgtatatccatgggctgcaggtgcaggaagtagcccttgctcctgggaagctccatagggacactgccagctgaaagcccca
>IBE-C5372_niethammeri_A
ggctggggaagcacaggttccaaatgtgatgatgttagaggccgggtttcgtccagcagaggaggaccattggaacctgattcagcctgcacagctgctttcatttct
ggccatgtatatccatgggctgcaggtgcaggaagtagcccttgctcctgggaagctccatagggacactgccagctgaaagcccca
>IBE-C5372_niethammeri_B
ggctggggaagcacaggttccaaatgtgatgatgttagaggccgggtttcgtccagcagaggaggaccattggaacctgattcagcctgcacagctgctttcatttct
ggccatgtatatccatgggctgcaggtgcaggaagtagcccttgctcctgggaagctccatagggacactgccagctgaaagcccca
>IBE-C5374_fodiens_A
ggctggggaagcacaggttccaaatgtgatgatgttagaggccgggtttcgtccagcagaggaggaccattggaacctgattcagcctgcacagctgctttcatttct
ggccatgtatatccatgggctgcaggtgcaggaagtagcccttgctcctgggaagctccatagggacactgccagctgaaagcccca
>IBE-C5374_fodiens_B
ggctggggaagcacaggttccaaatgtgatgatgttagaggccgggtttcgtccagcagaggaggaccattggaacctgattcagcctgcacagctgctttcatttct
ggccatgtatatccatgggctgcaggtgcaggaagtagcccttgctcctgggaagctccatagggacactgccagctgaaagcccca
>IBE-C5376_fodiens_A
ggctggggaagcacaggttccaaatgtgatgatgttagaggccgggtttcgtccagcagaggaggaccattggaacctgattcagcctgcacagctgctttcatttct
ggccatgtatatccatgggctgcaggtgcaggaagtagcccttgctcctgggaagctccatagggacactgccagctgaaagcccca
>IBE-C5376_fodiens_B
ggctggggaagcacaggttccaaatgtgatgatgttagaggccgggtttcgtccagcagaggaggaccattggaacctgattcagcctgcacagctgctttcatttct
ggccatgtatatccatgggctgcaggtgcaggaagtagcccttgctcctgggaagctccatagggacactgccagctgaaagcccca
>IBE-C5726_anomalus_A
ggctggggaagcacaggttccaaatgtgatgatgttagaggccaggtttcgtccagcagaggaggaccattggaacctgattcagcctgcacagctgctttcatttct
ggccatgtacatccatgggctgcaggtgcaggaagtagcccttgctcctgggaagctccatagggacactgccatctgaaagcccca
>IBE-C5726_anomalus_B
ggctggggaagcacaggttccaaatgtgatgatgttagaggccaggtttcgtccagcagaggaggaccattggaacctgattcagcctgcacagctgctttcatttct
ggccatgtacatccatgggctgcaggtgcaggaagtagcccttgctcctgggaagctccatagggacactgccatctgaaagcccca
>IBE-C5742_fodiens_A
ggctggggaagcacaggttccaaatgtgatgatgttagaggccgggtttcgtccagcagaggaggaccattggaacctgattcagcctgcacagctgctttcatttct
ggccatgtatatccatgggctgcaggtgcaggaagtagcccttgctcctgggaagctccatagggacactgccagctgaaagcccca
>IBE-C5742_fodiens_B
ggctggggaagcacaggttccaaatgtgatgatgttagaggccgggtttcgtccagcagaggaggaccattggaacctgattcagcctgcacagctgctttcatttct
ggccatgtatatccatgggctgcaggtgcaggaagtagcccttgctcctgggaagctccatagggacactgccagctgaaagcccca
>IBE-C5744_fodiens_A
ggctggggaagcacaggttccaaatgtgatgatgttagaggccgggtttcgtccagcagaggaggaccattggaacctgattcagcctgcacagctgctttcatttct
ggccatgtatatccatgggctgcaggtgcaggaagtagcccttgctcctgggaagctccatagggacactgccagctgaaagcccca
>IBE-C5744_fodiens_B
ggctggggaagcacaggttccaaatgtgatgatgttagaggccgggtttcgtccagcagaggaggaccattggaacctgattcagcctgcacagctgctttcatttct
ggccatgtatatccatgggctgcaggtgcaggaagtagcccttgctcctgggaagctccatagggacactgccagctgaaagcccca
>IBE-C6063_fodiens_A
ggctggggaagcacaggttccaaatgtgatgatgttagaggccgggtttcgtccagcagaggaggaccattggaacctgattcagcctgcacagctgctttcatttct
ggccatgtatatccatgggctgcaggtgcaggaagtagcccttgctcctgggaagctccatagggacactgccagctgaaagcccca
>IBE-C6063_fodiens_B
ggctggggaagcacaggttccaaatgtgatgatgttagaggccgggtttcgtccagcagaggaggaccattggaacctgattcagcctgcacagctgctttcatttct
ggccatgtatatccatgggctgcaggtgcaggaagtagcccttgctcctgggaagctccatagggacactgccagctgaaagcccca
>IBE-C6064_fodiens_A
ggctggggaagcacaggttccaaatgtgatgatgttagaggccgggtttcgtccagcagaggaggaccattggaacctgattcagcctgcacagctgctttcatttct
ggccatgtatatccatgggctgcaggtgcaggaagtagcccttgctcctgggaagctccatagggacactgccagctgaaagcccca
>IBE-C6064_fodiens_B
ggctggggaagcacaggttccaaatgtgatgatgttagaggccgggtttcgtccagcagaggaggaccattggaacctgattcagcctgcacagctgctttcatttct
ggccatgtatatccatgggctgcaggtgcaggaagtagcccttgctcctgggaagctccatagggacactgccagctgaaagcccca
>IBE-C6065_anomalus_A
ggctggggaagcacaggttccaaatgtgatgatgttagaggccaggtttcgtccagcagaggaggaccattggaacctgattcagcctgcacagctgctttcatttct
ggccatgtacatccatgggctgcaggtgcaggaagtagcccttgctcctgggaagctccatagggacactgccatctgaaagccccg
>IBE-C6065_anomalus_B
ggctggggaagcacaggttccaaatgtgatgatgttagaggccaggtttcgtccagcagaggaggaccattggaacctgattcagcctgcacagctgctttcatttct
ggccatgtacatccatgggctgcaggtgcaggaagtagcccttgctcctgggaagctccatagggacactgccatctgaaagccccg
>IBE-C6066_fodiens_A
ggctggggaagcacaggttccaaatgtgatgatgttagaggccgggtttcgtccagcagaggaggaccattggaacctgattcagcctgcacagctgctttcatttct
ggccatgtatatccatgggctgcaggtgcaggaagtagcccttgctcctgggaagctccatagggacactgccagctgaaagcccca
>IBE-C6066_fodiens_B

ggctggggaagcacaggttccaaatgtgatgatgttagaggccgggtttcgtccagcagaggaggaccattggaacctgattcagcctgcacagctgctttcatttct
ggccatgtatatccatgggctgcaggtgcaggaagtagcccttgctcctgggaagctccatagggacactgccagctgaaagcccca
>IBE-C6069_fodiens_A
ggctggggaagcacaggttccaaatgtgatgatgttagaggccgggtttcgtccagcagaggaggaccattggaacctgattcagcctgcacagctgctttcatttct
ggccatgtatatccatgggctgcaggtgcaggaagtagcccttgctcctgggaagctccatagggacactgccagctgaaagcccca
>IBE-C6069_fodiens_B
ggctggggaagcacaggttccaaatgtgatgatgttagaggccgggtttcgtccagcagaggaggaccattggaacctgattcagcctgcacagctgctttcatttct
ggccatgtatatccatgggctgcaggtgcaggaagtagcccttgctcctgggaagctccatagggacactgccagctgaaagcccca
>IBE-C4117_fodiens_A
ggctggggaagcacaggttccaaatgtgatgatgttagaggccgggtttcgtccagcagaggaggaccattggaacctgattcagcctgcacagctgctttcatttct
ggccatgtatatccatgggctgcaggtgcaggaagtagcccttgctcctgggaagctccatagggacactgccagctgaaagcccca
>IBE-C4117_fodiens_B
ggctggggaagcacaggttccaaatgtgatgatgttagaggccgggtttcgtccagcagaggaggaccattggaacctgattcagcctgcacagctgctttcatttct
ggccatgtatatccatgggctgcaggtgcaggaagtagcccttgctcctgggaagctccatagggacactgccagctgaaagcccca
>IBE-C6105_niethammeri_A
ggctggggaagcacaggttccaaatgtgatgatgttagaggccgggtttcgtccagcagaggaggaccattggaacctgattcagcctgcacagctgctttcatttct
ggccatgtatatccatgggctgcaggtgcaggaagtagcccttgctcctgggaagctccatagggacactgccagctgaaagcccca
>IBE-C6105_niethammeri_B
ggctggggaagcacaggttccaaatgtgatgatgttagaggccgggtttcgtccagcagaggaggaccattggaacctgattcagcctgcacagctgctttcatttct
ggccatgtatatccatgggctgcaggtgcaggaagtagcccttgctcctgggaagctccatagggacactgccagctgaaagcccca
>IBE-C6108_niethammeri_A
ggctggggaagcacaggttccaaatgtgatgatgttagaggccgggtttcgtccagcagaggaggaccattggaacctgattcagcctgcacagctgctttcatttct
ggccatgtatatccatgggctgcaggtgcaggaagtagcccttgctcctgggaagctccatagggacactgccagctgaaagcccca
>IBE-C6108_niethammeri_B
ggctggggaagcacaggttccaaatgtgatgatgttagaggccgggtttcgtccagcagaggaggaccattggaacctgattcagcctgcacagctgctttcatttct
ggccatgtatatccatgggctgcaggtgcaggaagtagcccttgctcctgggaagctccatagggacactgccagctgaaagcccca
>IBE-C6118_anomalus_A
ggctggggaagcacaggttccaaatgtgatgatgttagaggccaggtttcgtccagcagaggaggaccattggaacctgattcagcctgcacagctgctttcatttct
ggccatgtacatccatgggctgcaggtgcaggaagtagcccttgctcctgggaagctccatagggacactgccatctgaaagccccg
>IBE-C6118_anomalus_B
ggctggggaagcacaggttccaaatgtgatgatgttagaggccaggtttcgtccagcagaggaggaccattggaacctgattcagcctgcacagctgctttcatttct
ggccatgtacatccatgggctgcaggtgcaggaagtagcccttgctcctgggaagctccatagggacactgccatctgaaagccccg
>IBE-C6121_anomalus_A
ggctggggaagcacaggttccaaatgtgatgatgttagaggccaggtttcgtccagcagaggaggaccattggaacctgattcagcctgcacagctgctttcatttct
ggccatgtacatccatgggctgcaggtgcaggaagtagcccttgctcctgggaagctccatagggacactgccatctgaaagccccg
>IBE-C6121_anomalus_B
ggctggggaagcacaggttccaaatgtgatgatgttagaggccaggtttcgtccagcagaggaggaccattggaacctgattcagcctgcacagctgctttcatttct
ggccatgtacatccatgggctgcaggtgcaggaagtagcccttgctcctgggaagctccatagggacactgccatctgaaagcccca
>IBE-C6110_niethammeri_A
ggctggggaagcacaggttccaaatgtgatgatgttagaggccgggtttcgtccagcagaggaggaccattggaacctgattcagcctgcacagctgctttcatttct
ggccatgtatatccatgggctgcaggtgcaggaagtagcccttgctcctgggaagctccatagggacactgccagctgaaagcccca
>IBE-C6110_niethammeri_B
ggctggggaagcacaggttccaaatgtgatgatgttagaggccgggtttcgtccagcagaggaggaccattggaacctgattcagcctgcacagctgctttcatttct
ggccatgtatatccatgggctgcaggtgcaggaagtagcccttgctcctgggaagctccatagggacactgccagctgaaagcccca
>IBE-C6116_niethammeri_A
ggctggggaagcacaggttccaaatgtgatgatgttagaggccgggtttcgtccagcagaggaggaccattggaacctgattcagcctgcacagctgctttcatttct
ggccatgtatatccatgggctgcaggtgcaggaagtagcccttgctcctgggaagctccatagggacactgccagctgaaagcccca
>IBE-C6116_niethammeri_B
ggctggggaagcacaggttccaaatgtgatgatgttagaggccgggtttcgtccagcagaggaggaccattggaacctgattcagcctgcacagctgctttcatttct
ggccatgtatatccatgggctgcaggtgcaggaagtagcccttgctcctgggaagctccatagggacactgccagctgaaagcccca
>IBE-C6124_anomalus_A
ggctggggaagcacaggttccaaatgtgatgatgttagaggccaggtttcgtccagcagaggaggaccattggaacctgattcagcctgcacagctgctttcatttct
ggccatgtacatccatgggctgcaggtgcaggaagtagcccttgctcctgggaagctccatagggacactgccatctgaaagccccg
>IBE-C6124_anomalus_B
ggctggggaagcacaggttccaaatgtgatgatgttagaggccaggtttcgtccagcagaggaggaccattggaacctgattcagcctgcacagctgctttcatttct
ggccatgtacatccatgggctgcaggtgcaggaagtagcccttgctcctgggaagctccatagggacactgccatctgaaagccccg
>IBE-C5359_niethammeri_A
ggctggggaagcacaggttccaaatgtgatgatgttagaggccgggtttcgtccagcagaggaggaccattggaacctgattcagcctgcacagctgctttcatttct
ggccatgtatatccatgggctgcaggtgcaggaagtagcccttgctcctgggaagctccatagggacactgccagctgaaagcccca
>IBE-C5359_niethammeri_B
ggctggggaagcacaggttccaaatgtgatgatgttagaggccgggtttcgtccagcagaggaggaccattggaacctgattcagcctgcacagctgctttcatttct
ggccatgtatatccatgggctgcaggtgcaggaagtagcccttgctcctgggaagctccatagggacactgccagctgaaagcccca
>IBE-C5371_niethammeri_A
ggctggggaagcacaggttccaaatgtgatgatgttagaggccgggtttcgtccagcagaggaggaccattggaacctgattcagcctgcacagctgctttcatttct
ggccatgtatatccatgggctgcaggtgcaggaagtagcccttgctcctgggaagctccatagggacactgccagctgaaagcccca
>IBE-C5371_niethammeri_B
ggctggggaagcacaggttccaaatgtgatgatgttagaggccgggtttcgtccagcagaggaggaccattggaacctgattcagcctgcacagctgctttcatttct
ggccatgtatatccatgggctgcaggtgcaggaagtagcccttgctcctgggaagctccatagggacactgccagctgaaagcccca
>MSB-158495_fodiens_A
ggctggggaagcacaggttccaaatgtgatgatgttagaggccgggtttcgtccagcagaggaggaccattggaacctgattcagcctgcacagctgctttcatttct
ggccatgtatatccatgggctgcaggtgcaggaagtagcccttgctcctgggaagctccatagggacactgccagctgaaagcccca
>MSB-158495_fodiens_B
ggctggggaagcacaggttccaaatgtgatgatgttagaggccgggtttcgtccagcagaggaggaccattggaacctgattcagcctgcacagctgctttcatttct
ggccatgtatatccatgggctgcaggtgcaggaagtagcccttgctcctgggaagctccatagggacactgccagctgaaagcccca

>MSB-158621_fodiens_A
ggctggggaagcacaggttccaaatgtgatgatgttagaggccgggtttcgtccagcagaggaggaccattggaacctgattcagcctgcacagctgctttcatttct
ggccatgtatatccatgggctgcaggtgcaggaagtagcccttgctcctgggaagctccatagggacactgccagctgaaagcccca
>MSB-158621_fodiens_B
ggctggggaagcacaggttccaaatgtgatgatgttagaggccgggtttcgtccagcagaggaggaccattggaacctgattcagcctgcacagctgctttcatttct
ggccatgtatatccatgggctgcaggtgcaggaagtagcccttgctcctgggaagctccatagggacactgccagctgaaagcccca
>MSB-288577_fodiens_A
ggctggggaagcacaggttccaaatgtgatgatgttagaggccgggtttcgtccagcagaggaggaccattggaacctgattcagcctgcacagctgctttcatttct
ggccatgtatatccatgggctgcaggtgcaggaagtagcccttgctcctgggaagctccatagggacactgccagctgaaagcccca
>MSB-288577_fodiens_B
ggctggggaagcacaggttccaaatgtgatgatgttagaggccgggtttcgtccagcagaggaggaccattggaacctgattcagcctgcacagctgctttcatttct
ggccatgtatatccatgggctgcaggtgcaggaagtagcccttgctcctgggaagctccatagggacactgccagctgaaagcccca
>MSB-288698_fodiens_A
ggctggggaagcacaggttccaaatgtgatgatgttagaggccgggtttcgtccagcagaggaggaccattggaacctgattcagcctgcacagctgctttcatttct
ggccatgtatatccatgggctgcaggtgcaggaagtagcccttgctcctgggaagctccatagggacactgccagctgaaagcccca
>MSB-288698_fodiens_B
ggctggggaagcacaggttccaaatgtgatgatgttagaggccgggtttcgtccagcagaggaggaccattggaacctgattcagcctgcacagctgctttcatttct
ggccatgtatatccatgggctgcaggtgcaggaagtagcccttgctcctgggaagctccatagggacactgccagctgaaagcccca
>MSB-293526_fodiens_A
ggctggggaagcacaggttccaaatgtgatgatgttagaggccgggtttcgtccagcagaggaggaccattggaacctgattcagcctgcacagctgctttcatttct
ggccatgtatatccatgggctgcaggtgcaggaagtagcccttgctcctgggaagctccatagggacactgccagctgaaagcccca
>MSB-293526_fodiens_B
ggctggggaagcacaggttccaaatgtgatgatgttagaggccgggtttcgtccagcagaggaggaccattggaacctgattcagcctgcacagctgctttcatttct
ggccatgtatatccatgggctgcaggtgcaggaagtagcccttgctcctgggaagctccatagggacactgccagctgaaagcccca
>MSB-95472_fodiens_A
ggctggggaagcacaggtttcaaatgtgatgatgttagaggccgggtttcgtccagcagaggaggaccattggaacctgattcagcctgcacagctgctttcatttct
ggccatgtatatccatgggctgcaggtgcaggaagtagcccttgctcctgggaagctccatagggacactgccagctgaaagcccca
>MSB-95472_fodiens_B
ggctggggaagcacaggtttcaaatgtgatgatgttagaggccgggtttcgtccagcagaggaggaccattggaacctgattcagcctgcacagctgctttcatttct
ggccatgtatatccatgggctgcaggtgcaggaagtagcccttgctcctgggaagctccatagggacactgccagctgaaagcccca
>IBE-C4515_anomalus_A
ggctggggaagcacaggttccaaatgtgatgatgttagaggccaggtttcgtccagcagaggaggaccattggaacctgattcagcctgcacagctgctttcatttct
ggccatgtacatccatgggctgcaggtgcaggaagtagcccttgctcctgggaagctccatagggacactgccatctgaaagccccg
>IBE-C4515_anomalus_B
ggctggggaagcacaggttccaaatgtgatgatgttagaggccaggtttcgtccagcagaggaggaccattggaacctgattcagcctgcacagctgctttcatttct
ggccatgtacatccatgggctgcaggtgcaggaagtagcccttgctcctgggaagctccatagggacactgccatctgaaagccccg
